# A family of NADPH/NADP^+^ biosensors reveals in vivo dynamics of central redox metabolism across eukaryotes

**DOI:** 10.1038/s41467-024-55302-x

**Published:** 2024-12-19

**Authors:** Marie Scherschel, Jan-Ole Niemeier, Lianne J. H. C. Jacobs, Markus D. A. Hoffmann, Anika Diederich, Christopher Bell, Pascal Höhne, Sonja Raetz, Johanna B. Kroll, Janina Steinbeck, Sophie Lichtenauer, Jan Multhoff, Jannik Zimmermann, Tanmay Sadhanasatish, R. Alexander Rothemann, Carsten Grashoff, Joris Messens, Emmanuel Ampofo, Matthias W. Laschke, Jan Riemer, Leticia Prates Roma, Markus Schwarzländer, Bruce Morgan

**Affiliations:** 1 Institute of Biochemistry, Center for Human and Molecular Biology (ZHMB), Saarland University, Saarbrücken, Germany; 2 Institute of Plant Biology and Biotechnology, University of Münster, Schlossplatz 8, Münster, Germany; 3 Redox Metabolism, Institute for Biochemistry, University of Cologne, Cologne, Germany; 4 Department of Biophysics, Center for Human and Molecular Biology (ZHMB), Saarland University, Homburg, Germany; 5 Institute of Integrative Cell Biology and Physiology, University of Münster, Schlossplatz 5, Münster, Germany; 6 VIB-VUB Center for Structural Biology, Vlaams Instituut voor Biotechnologie, Brussels, Belgium; 7 Brussels Center for Redox Biology, Vrije Universiteit Brussel, Brussels, Belgium; 8 Structural Biology Brussels, Vrije Universiteit Brussel, Brussels, Belgium; 9 Institute for Clinical & Experimental Surgery, Saarland University, Homburg, Germany; 10 Cologne Excellence Cluster on Cellular Stress Responses in Aging-associated Diseases (CECAD), University of Cologne, Cologne, Germany

**Keywords:** Fluorescent proteins, Oxidoreductases, Metabolic pathways

## Abstract

The NADPH/NADP^+^ redox couple is central to metabolism and redox signalling. NADP redox state is differentially regulated by distinct enzymatic machineries at the subcellular compartment level. Nonetheless, a detailed understanding of subcellular NADP redox dynamics is limited by the availability of appropriate tools. Here, we introduce NAPstars, a family of genetically encoded, fluorescent protein-based NADP redox state biosensors. NAPstars offer real-time, specific measurements, across a broad-range of NADP redox states, with subcellular resolution. NAPstar measurements in yeast, plants, and mammalian cell models, reveal a conserved robustness of cytosolic NADP redox homoeostasis. NAPstars uncover cell cycle-linked NADP redox oscillations in yeast and illumination- and hypoxia-dependent NADP redox changes in plant leaves. By applying NAPstars in combination with selective impairment of the glutathione and thioredoxin antioxidative pathways under acute oxidative challenge, we find an unexpected and conserved role for the glutathione system as the primary mediator of antioxidative electron flux.

## Abstract (web summary)

This article presents NAPstars, a family of genetically-encoded biosensors that enable real-time monitoring of NADP redox dynamics across species. The sensors reveal robust NADP redox regulation, cell-cycle-linked NADP oscillations, and glutathione as the major conduit for anti-oxidative electron flux.

## Introduction

Nicotinamide adenine dinucleotide phosphate (NADP), in its reduced (NADPH) and oxidised (NADP^+^) states, constitutes a central metabolic redox couple, which is found in all living organisms. NADPH plays a crucial role as an electron donor in numerous, typically anabolic, pathways, for example fatty acid and cholesterol synthesis and photosynthetic carbon assimilation, and for the enzymatic reduction of certain reactive oxygen species, including H_2_O_2_. NADPH also serves as the source of electrons for NADPH oxidases. This family of enzymes is central to the respiratory burst in immune responses and the generation of H_2_O_2_ as a second messenger in cellular signalling, development, and environmental sensing across kingdoms^1,2^.

Since its discovery in the early 1930s^3,4^, NADP, together with its cellular counterpart, NAD, has been extensively investigated. However, our understanding of subcellular NAD(P) redox dynamics is still remarkably incomplete, mainly due to a lack of techniques allowing specific monitoring in defined subcellular compartments in vivo.

The use of novel genetically encoded sensors allowed accurate measurements of the subcellular dynamics of numerous metabolites, metals ions including free Ca^2+^, H_2_O_2_, and pH^5–8^. Such sensors have revolutionised our understanding of countless previously intractable biological processes, particularly when dealing with small subcellular compartment-specific dynamics, and have underpinned numerous novel discoveries. Genetically encoded sensors for pyridine nucleotides were relative latecomers; however, significant progress has been made over the past decade in developing sensors for NADH^9^, NAD^+^^10,11^, NADH/NAD^+^^12–14^, NADPH^15^, and NADP^+^^16,17^. Nonetheless, existing NADPH sensors face major limitations, including sensitivity to pH, a lack of responsiveness to NADP^+^, a low signal-to-noise ratio, and a lack of compatibility with alternative measurement techniques like FLIM. A recently created sensor, NERNST, was developed, which purported to be the first NADP redox state sensor, i.e. a sensor of the NADPH/NADP^+^ ratio^18^. Nonetheless, considerable concerns arise regarding the specificity of NERNST due to its dependence on a redox-sensitive green fluorescent protein (roGFP2) reporter, which is known to efficiently equilibrate with the glutathione redox couple in vivo^19–22^.

In this study, we employed a rational probe design strategy to create the NAPstar family of NADP redox state probes. The NAPstar family allows the monitoring of NADP redox states across a 5000-fold range, spanning NADPH/NADP^+^ ratios from approximately 0.001 to 5. We showed that NAPstars facilitate specific and real-time monitoring of subcellular NADP redox state dynamics, which can be measured either by monitoring changes in fluorescence excitation and emission or through fluorescence lifetime imaging. By applying NAPstars in yeast,human cells and plants, we found a surprisingly oxidised cytosolic NADP redox state, albeit with a remarkable robustness to oxidative challenges in yeast. We used NAPstars to reveal oscillations in the NADP redox state associated with cell division and metabolic cycles in yeast and to monitor NADP redox dynamics influenced by illumination and hypoxia–reoxygenation in plants. Finally, we revealed a predominant role of the glutathione system in mediating antioxidative electron flux in response to oxidative challenges that is conserved across diverse eukaryotic cells.

## Results

### Peredox mutagenesis delivers a family of NADP redox state sensors

**Fig. 1:**
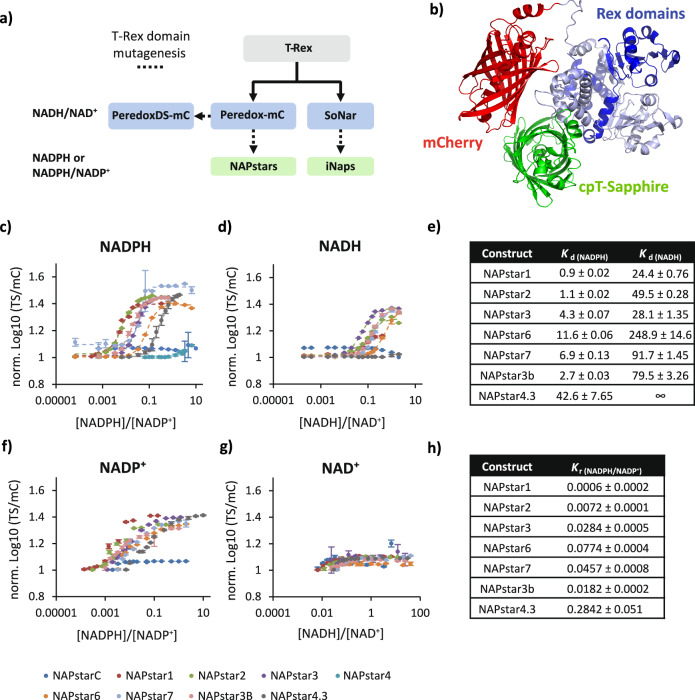
NAPstars respond specifically to changes in the NADP redox state. **a** Diagram showing the development of selected NAD and NADP sensors including NAPstars. **b** AlphaFold2 prediction of NAPstar structure. Graphs showing the normalised logarithm of the cpT-Sapphire/mCherry fluorescence ratio at **c** different NADPH/NADP^+^ and **d** NADH/NAD^+^ ratios. NADPH and NADH concentration was titrated against a fixed background of 150 µM NADP^+^ and 500 µM NAD^+^ respectively. In **c** and **d**, the dashed lines show a fitted sigmoidal function that was used to determine *K*_d(NAD(P)H)_. **e** Table summarising the determined *K*_d(NADPH)_ and *K*_d(NADH)_ values of all NAPstars. Graphs showing the normalised logarithm of the cpT-Sapphire/mCherry fluorescence ratio at **f** different NADPH/NADP^+^ and **g** NADH/NAD^+^ ratios. NADP^+^ and NAD^+^ concentration was titrated against a fixed concentration of NADPH and NADH respectively that for each probe corresponded approximately to the determined *K*_d(NADPH)_ and *K*_d(NADH)_ values. This experimental regime, by definition, only allows a maximum of approximately 50% NADPH binding and explains the difference in shape of the titration curve between panels **c** and **f**. **h** Table summarising the determined *K*_r(NADPH/NADP+)_ for all NAPstars. For panels **c**, **d**, **f**, **g**, *n* = 3 technical replicates. Data are presented as mean ± s.d. normalised to the lowest data point.

We used the NAD redox state sensor, Peredox-mCherry (henceforth referred to as Peredox)^13^, as a chassis for the development of NADP redox state probes (Fig. 1a). Peredox incorporates a circularly permuted T-Sapphire (TS) fluorescent protein nested between two copies of the NADH/NAD^+^-binding domain of the bacterial transcriptional repressor Rex^13^. Structural changes, contingent on whether the Rex domains bind to NAD^+^ or NADH, induce changes in the TS fluorescence. This fluorescence change can be normalised against the signal from a C-terminally fused mCherry (mC) fluorescent protein. Peredox offers several advantages over other NADH and NADPH sensors, including limited pH sensitivity and the high apparent brightness of TS-based probes in biological systems relative to some cpYFP-based sensors such as SoNar and the iNap family^12,15^.

To alter the binding pocket of Peredox to favour NADP binding, we introduced mutations known to switch the specificity of the Rex domain from favouring NADH binding to favouring NADPH^15^ and generated combinatorial mutants thereof. These mutations were applied equally to each of the Rex domains of Peredox, resulting in a family of constructs termed NAPstars (Supplementary Table 1). To gain insight into the potential structure of NAPstar, we used AlphaFold2 with the 919 amino acids of NAPstar3 as the input (Supplementary Information). The sequence consists of a Rex domain linked with an SAAGGH amino acid sequence to a circular permutated TS, followed by a single amino acid Thr linked to a second Rex domain, a GSGTGGNASDGGGSGG linker, and the mCherry sequence. Five models were generated, and the top-ranked model displayed a reliable structure, with an average predicted Local Distance Difference Test (pLDDT) score of 87.8% (Fig. 1b).

For in vitro characterisation, NAPstar1, 2, 3, 4, 6, 7 and NAPstarC were expressed as recombinant proteins in *E. coli*. Fluorescence spectra were recorded in the presence of 0–100 µM NADPH (Supplementary Fig. 1). NAPstars1, 2, 3, 6 and 7 exhibited pronounced NADPH-dependent changes in TS fluorescence excitation and emission spectra, with excitation and emission maxima at approximately 400 and 515 nm, respectively, and with a spectroscopic dynamic range of approximately 2.5, i.e. about 0.4 units on a log10 scale, similar to Peredox, (Fig. 1c)^23^. NAPstarC, which contains mutations preventing nucleotide binding to Rex, remained unresponsive to changes in the NADPH concentration (Supplementary Fig. 1a, b). Similarly, NAPstar4 did not show any NADPH-dependent change in the tested range and was therefore excluded from further characterisation. Changes in NADPH concentration did not affect the fluorescence excitation or emission of mC (Supplementary Fig. 1d, f, h, j, l, n). Our results thus demonstrate that NAPstars respond to changes in NADPH concentration.

To better characterise the pyridine nucleotide specificity of the NAPstar constructs, we titrated NAPstar1, 2, 3, 4, 6 and 7 with varying concentrations of NADPH, NADP^+^, NADH or NAD^+^ (Fig. 1c, d, f, g). The NADPH concentration was adjusted from 0.01–1000 µM in the presence of a constant 150 µM NADP^+^. For all constructs, except for the non-binding control, NAPstarC, and NAPstar4, we observed an NADPH concentration-dependent change in the TS/mC fluorescence emission ratio (Fig. 1c). We determined apparent dissociation constants for NADPH (*K*_d(NADPH)_) from the fluorescence changes, which ranged from 0.9 µM for NAPstar1 to 11.6 µM for NAPstar6 (Fig. 1e). All NAPstars showed some affinity to NADH, titrated in the presence of 500 µM NAD^+^, with *K*_d(NADH)_ ranging from 24.4–248.9 µM. However, the affinity for NADH is substantially weaker than that of Peredox (*K*_d(NADH)_ = 1.2 µM)^23^ and for each NAPstar is one to two orders of magnitude lower than the affinity for NADPH (Fig. 1d, e). For all NAPstars, we also observed ratiometric fluorescence responses to NADP^+^ in the opposite direction to those induced by NADPH (Fig. 1f). These changes were not observed for NAD^+^ (Fig. 1g). These results thus suggest that NAPstars report the bona fide NADP redox state rather than responding solely to the NADPH concentration. To further investigate this, we monitored the dependence of the TS/mC ratio on the NADPH/NADP^+^ ratio at different total NADP (NADPH + NADP^+^) pool sizes of 100, 300 and 500 µM respectively (Supplementary Fig. 2). The responses of NAPstar sensors remained largely stable across various pool sizes, indicating a predominant sensitivity to the NADP redox state rather than the individual concentrations of NADPH or NADP^+^. Exceptions seem to be NAPstar6 and 7, which displayed some dependence on pool size. These two variants also exhibited the highest *K*_d(NADPH)_. To reflect the fact that NAPstars measure the NADPH/NADP^+^ ratio, rather than the NADPH concentration, from now on we report *K*_ratios_ (*K*_r(NADPH/NADP+)_) for every NAPstar instead of *K*_d(NADPH)_ (Fig. 1h).

### Mixing different Rex domains to enhance the control of NAD(P)H binding affinity

NAPstars contain both Rex domains needed for NADP binding. In contrast, iNaps presumably require dimerisation to form the Rex-dimer for a functional sensor unit, adding sensor concentration (i.e. expression levels) as an additional variable^15^. We asked if we could exploit the fact that NAPstars encompass a Rex dimer within one polypeptide, by introducing a blend of two different Rex domains into one NAPstar sensor, with the aim to expand the range of accessible NADP redox states. As a first attempt, we created NAPstar3b, which features a single point mutation, V126Y, in the N-terminal NAPstar3-derived Rex domain instead of the usual V130Y mutation which was kept in the second, C-terminal, NAPstar3-derived Rex domain. NAPstar3b was found to have a slightly decreased *K*_d(NADPH)_ but an approximately 3-fold higher *K*_d(NADH)_ (Fig. 1c–h). Building on this idea, we further pushed the boundaries by combining an N-terminal Rex domain from NAPstar4, a NAPstar variant initially dismissed due to a lack of detectable affinity for NADPH (Fig. 1c), with a C-terminal Rex domain from NAPstar3. The resulting construct, NAPstar4.3, was purified as a recombinant protein for in vitro characterisation (Fig. 1c–h). Intriguingly, we found that NAPstar4.3 had a strongly decreased affinity for NADPH, *K*_d (NADPH)_ = 42.6 µM, *K*_r(NADPH/NADP+)_ = 0.28 (Fig. 1c,e), and no detectable affinity for NADH (Fig. 1d, e). This suggests that combining different mutated Rex domains within a single NAPstar construct provides enhanced control to rationally modulate NAPstar binding properties.

### The NAPstars retain the limited pH sensitivity from the Peredox chassis

Resistance of the fluorescence excitation and emission spectra to pH changes is a major achievement of the molecular engineering of the Peredox probe^13^ and there is no reason to believe that this characteristic would differ in the case of NAPstars. To verify whether the NAPstars do indeed retain the low sensitivity to pH as originally introduced into Peredox, we incubated each NAPstar probe in different buffers with pH values ranging from 6.0–9.0 (Supplementary Fig. 3a–h). Each NAPstar was incubated in the absence of NADPH and NADP^+^, in the presence of a high NADPH/NADP^+^ ratio mixture, and in the presence of a low NADPH/NADP^+^ ratio mixture. The TS/mC ratio of each NAPstar varied in a very similar manner as previously observed for Peredox-mCherry^23^ with a less than 20% change in the TS/mC ratio between pH 7.0 and 8.5. The change in TS/mC when NAPstars were incubated with high NADPH/NADP^+^ ratio mixtures was even lower, and remained close to constant between pH 7.0 and 8.5. At more acidic pH, a moderate drop in TS/mC ratio was observed, as previously shown for Peredox^13,23^. Nonetheless, the response of NAPstarC mimics the slightly larger response of the NADP^+^-bound NAPstar and thus NAPstarC represents a useful control for possible pH effects. As a note of caution, NAPstarC would not allow for control of potential pH-dependent differences in the binding affinity of NAPstars for NADP^+^ and NADPH. Nonetheless, we expect pH changes to be inconsequential for NAPstars responses in most physiological situations.

### NAPstars specifically respond to dynamic NADP redox changes in vitro

To further test the dynamic and specific responsiveness of NAPstars to changes in NADP redox status, we used recombinant NAPstar probes to monitor NADP dynamics in enzyme assays in vitro (Supplementary Fig. 4). First, we monitored the reduction of NADP^+^ to NADPH in the isocitrate dehydrogenase-catalysed reaction from isocitrate to α-ketoglutarate (Supplementary Fig. 4a). Upon the addition of isocitrate to start the reaction, the TS/mC ratio of NAPstar1, 2, and 3 rapidly increased. The response rate correlated with *K*_r(NADPH/NADP+)_, i.e. NAPstar1 and 2 responded most rapidly, followed by NAPstar3. No response was detected for NAPstarC. As a further control for the conversion of NADP^+^ to NADPH, we simultaneously detected an increase in NADPH autofluorescence after initiating the reaction.

Next, we monitored NADP oxidation, i.e. the conversion of NADPH to NADP^+^ during the glutathione reductase-catalysed reaction between NADPH and glutathione disulfide (GSSG) (Supplementary Fig. 4b). Upon the addition of GSSG, we observed a rapid decrease in the TS/mC ratio for each NAPstar construct except NAPstarC. Among the tested NAPstars, NAPstar3, which has the highest *K*_r(NADPH/NADP+)_, responded first to NADPH consumption, followed by NAPstar1 and NAPstar2.

Finally, we used NAPstars and Peredox to monitor the conversion of NADPH to NADH, which cannot be resolved using standard NAD(P)H autofluorescence (Supplementary Fig. 4c). Upon initiating the reaction with alkaline phosphatase, we observed a decrease in the TS/mC ratio, first for NAPstar3, followed by NAPstar2, and finally NAPstar1. The TS/mC of Peredox increased rapidly following the initiation of the reaction, consistent with an increase in the NADH concentration. In summary, these results demonstrate that NAPstars and Peredox allow the selective monitoring of NADP and NAD redox dynamics, respectively.

### The NAPstars are well-suited to FLIM measurements

Fluorescence lifetime imaging (FLIM) is a powerful alternative approach to monitor the status of fluorescent molecules and proteins and can be especially advantageous, for example in situations of low signal-to-noise at the fluorescence intensity level or where there are overlapping fluorescence excitation and emission spectra. We thus sought to test the suitability of NAPstar sensors for FLIM measurements (Supplementary Fig. 5). To this end, we monitored the fluorescence lifetime of NAPstar4.3 in the presence of different NADPH/NADP^+^ ratios. We observed a change in the fluorescence lifetime from 1.3 ns in an NADP^+^-saturated state to 2.3 ns in an NADPH-saturated state, which is exceptionally large for a fluorescent protein-based biosensor. Based on the FLIM data, we determined a *K*_d(NADPH)_ = 40.5 µM and *K*_r(NADPH/NADP+)_ = 0.27, which are very close to the 42.6 µM and 0.28 values determined by fluorescence intensity measurements (Fig. 1). We conclude that NAPstars have favourable characteristics for the development of FLIM-based measurement approaches.

### Cytosolic NADP redox homoeostasis is robustly maintained in yeast

**Fig. 2:**
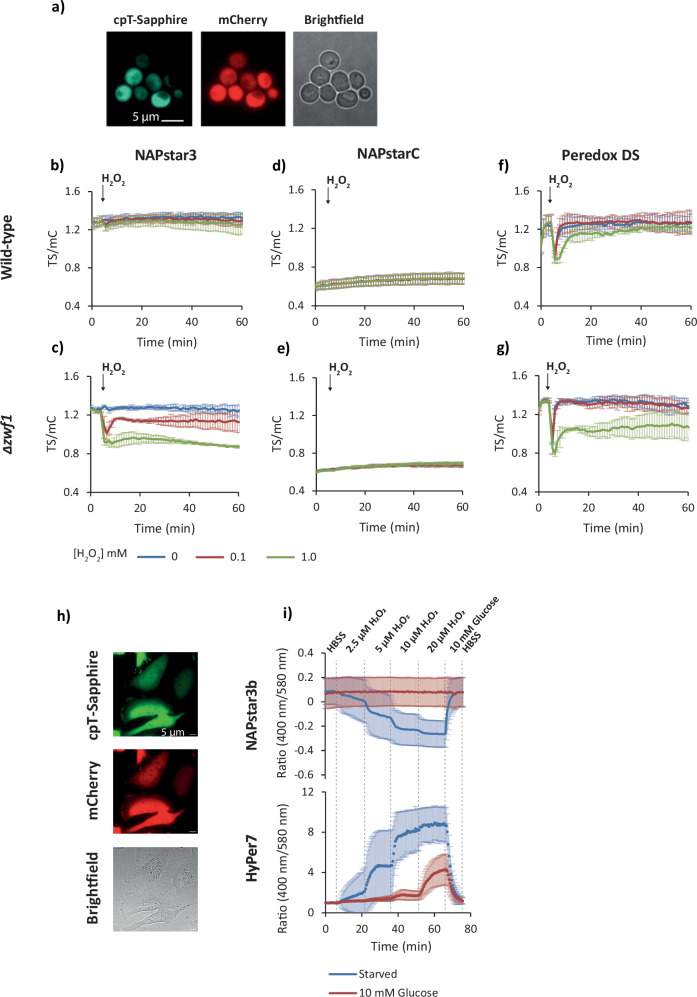
Cytosolic NADP redox homoeostasis is robustly maintained. **a** Epifluorescence microscopy images of yeast cells expressing NAPstar3, showing cpT-Sapphire and mCherry fluorescence as well as brightfield microscopy. Representative image; images were obtained from three independent yeast cultures. **b**–**g** Response of PeredoxDS, NAPstar3 and NAPstarC probes, expressed in the cytosol of wild-type and Δ*zwf1* yeast cells, to the addition of exogenous H_2_O_2_ at the indicated concentrations (*n* = 3 experimental repeats in which H_2_O_2_ responses were monitored in cells derived from independent cultures. Identical experimental settings were used for all panels allowing for direct comparison of TS/mC between datasets. **h** Epifluorescence microscopy images showing cpT-Sapphire and mCherry fluorescence of NAPstar3b expressed in the cytosol of HeLa cells. Representative image; images were obtained from four separate HeLa cell cultures. **i** Response to NAPstar3b (*n* = 148 individual cells for the starved condition and *n* = 151 individual cells for the glucose condition, monitored in both cases in the course of four experimental replicates) and HyPer7 (*n* = 144 individual cells for the starved condition and *n* = 154 individual cells for the glucose condition, monitored in both cases in the course of *n* = 3 experimental replicates) probes, expressed in the cytosol of HeLa cells cultured in a perfusion chamber, to the perfusion of buffers with stepwise increases in H_2_O_2_ concentration. Within each experimental replicate, the response was determined as the mean of the individual cell responses. Data are presented as the mean of experimental replicates with error bars representing the standard deviation between experimental replicates.

Given that NAPstars are indeed specific sensors of the NADP redox state in vitro, we next sought to test them in various eukaryotic model systems, starting with the budding yeast, *Saccharomyces cerevisiae*. We used a microplate-based assay to monitor the response of all NAPstars and PeredoxDS (an affinity variant of Peredox-mCherry for more reducing NAD redox states)^23^, expressed in the yeast cytosol, to exogenous H_2_O_2_ at initial concentrations ranging from 0–5 mM, as well as *tert*-butylperoxide (*t*-BuOOH) at 0–1 mM to avoid NADP-independent scavenging by catalase (Fig. 2 and Supplementary Figs. 6 and 7). Strikingly, even at the highest H_2_O_2_ concentration, a minimal response was detected for NAPstar1, 2 or 3 (Fig. 2c and Supplementary Fig. 6) Similar results were observed with *t*-BuOOH (Supplementary Fig. 7). NAPstar6 and 7, with a high *K*_r(NADPH/NADP+)_, showed a limited response with a rapid recovery, while the TS/mC ratio of NAPstar4.3 was close to that of NAPstarC (Supplementary Fig. 6). These observations are consistent with the relative affinities of the sensor variants determined in vitro (Fig. 1) and suggest robust and active maintenance of the cytosolic NADP redox state even under severe oxidative challenge, which is in stark contrast to previous observations of a highly volatile cytosolic NAD redox state as monitored by Peredox^13,23^. The similarity in the fluorescence ratio of NADP^+^-bound and unbound NAPstars (as for NAPstarC) is consistent with previously published crystal structures of *Thermotoga maritima* Rex (TmRex) in NADH-bound, NAD^+^-bound, and unbound (apo) states^24^. Unbound and NAD^+^-bound TmRex are structurally very similar, while NADH binding induces significant structural changes. This suggests that NAPstar4.3 is in an almost fully NADP^+^-bound state in the yeast cytosol and is unable to respond to oxidation of the NADP redox couple (Supplementary Fig. 6). The measurements allowed for an in vivo estimate for the NADPH/NADP^+^ ratio in the cultured yeast cytosol in the range of 0.03–0.3 (Figs. 1, 2 and Supplementary Fig. 6).

To distinguish between a bona fide robustness of cytosolic NADP redox maintenance and a potential lack of probe functionality in yeast, we sought to make NAPstar measurements in yeast cells with constrained cytosolic NADPH regeneration. To this end, we monitored the NAPstar responses in a Δ*zwf1* yeast strain. *ZWF1* encodes glucose 6-phosphate dehydrogenase, the first enzyme in the pentose phosphate pathway, which is the major source of cytosolic NADPH in yeast. In Δ*zwf1* cells, we observed sensitive, H_2_O_2_ and *t*-BuOOH concentration-dependent responses for all NAPstar constructs, supporting the conclusion that cytosolic NADP redox homoeostasis is robustly maintained in wild-type yeast by adjusting flux through the oxidative pentose phosphate pathway (Fig. 2 and Supplementary Fig. 6)^25–28^. This finding is reminiscent of our previous observations of the robustness of cytosolic glutathione redox homoeostasis, which largely relies on continuous NADPH supply, indicating that the robustness in NADP redox state also serves the stability of downstream redox pools^21^. NAPstarC did not respond in either WT or Δ*zwf1* cells (Fig. 2d, e) whilst Peredox responded similarly in both strains (Fig. 2f, g).

### The robustness of cytosolic NADP redox homoeostasis is conserved

We next sought to understand to what extent the principles of NADP redox maintenance apply beyond yeast. Hence, we introduced and validated NAPstars in additional eukaryotic models. To this end, we used transient transfection to introduce NAPstar3b into HeLa cells (Fig. 2h). NAPstar3b was chosen as due to its K_r(NADPH/NADP+)_ we expected it to be almost fully NADPH bound at steady state, therefore allowing for the maximum possible deflection upon NADP oxidation, and due to its particularly low NADH binding affinity. We were first interested in monitoring the response of NAPstar3b to exogenous H_2_O_2_ in cells subjected to glucose starvation, comparing them to cells continuously exposed to 10 mM glucose. In a perfusion chamber, we monitored the response of HeLa cells to stepwise increases in H_2_O_2_ concentration (Fig. 2i). In glucose-starved cells, we observed a stepwise increase in NADP oxidation (Fig. 2i). In contrast, in non-glucose starved cells, with the presence of 10 mM glucose in all perfusion buffers, no NADP oxidation was observed, even with continuous perfusion of buffer containing 20 µM H_2_O_2_ (Fig. 2i). Additionally, we used the ultra-sensitive H_2_O_2_ probe HyPer7^29^ to monitor H_2_O_2_ in the same experimental setup (Fig. 2i). Consistent with the NAPstar response, we observed almost no HyPer7 response in glucose-fed cells, except at the highest H_2_O_2_ concentration, suggesting that HeLa cells can very efficiently remove H_2_O_2_ as long as reductant can be efficiently delivered by metabolism. In contrast, in glucose-starved cells, we observed a strong HyPer7 response beginning with the lowest H_2_O_2_ concentration tested.

In summary, we found that, akin to the situation in yeast, cytosolic NADP redox homoeostasis in HeLa cells is robustly maintained, and NADPH is readily available for the reduction of exogenous H_2_O_2_. Robustly regulated cytosolic NADP redox homoeostasis hence may be a conserved physiological characteristic from yeast to human cells.

### Yeast cell cycle is accompanied by oscillations in NAD and NADP redox states

Confident that NAPstars and Peredox faithfully report changes in NADP and NAD redox states within the yeast cytosol, we tested whether we could use NAPstars and Peredox to investigate the existence of dynamic changes in pyridine nucleotide redox states during the yeast metabolic cycle (YMC). The YMC is a phenomenon observed in continuous yeast cultures under mild glucose limitation, involving synchronised metabolic and transcriptional cycles along with cell cycle synchronisation^30,31^. Previous reports have suggested that the total NAD(H) and NADP(H) levels change during the YMC^32^, or have shown changes in NAD(P)H^33^. However, measurement of changes in NADP, or NAD redox states, and the examination of specific subcellular NAD(P) pools has hitherto been impossible in this system.

**Fig. 3:**
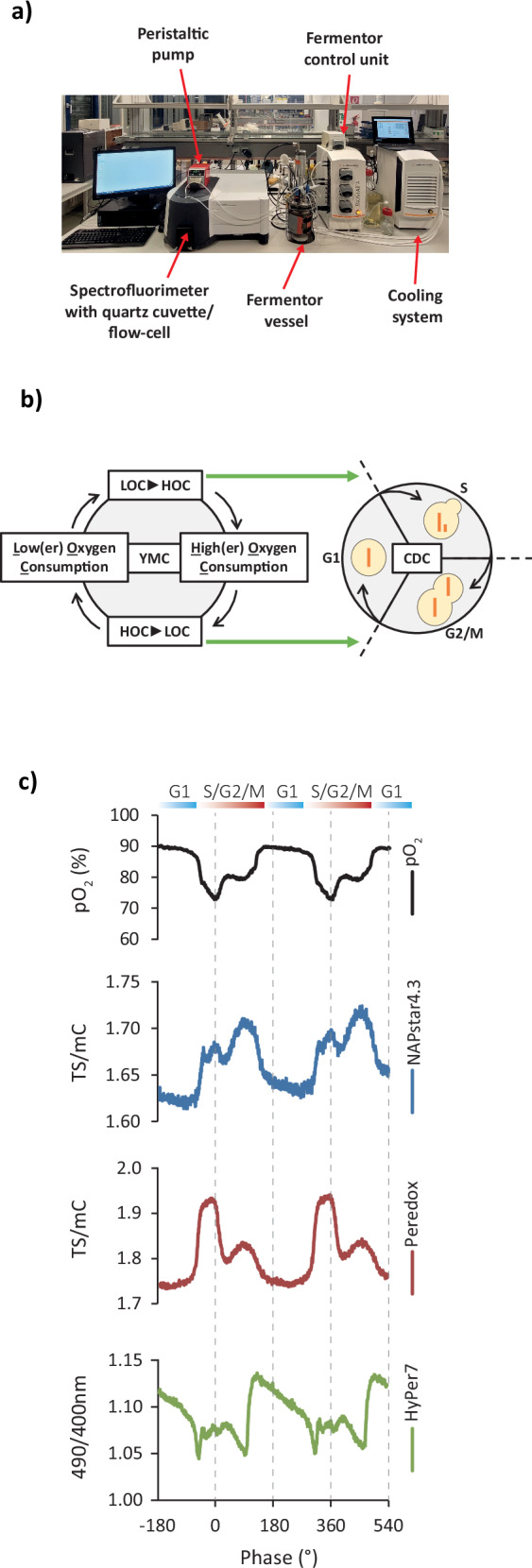
Oscillations in cytosolic NADP and NAD redox state accompany the yeast metabolic cycle (YMC). **a** Photograph of the coupled fermentor–fluorimeter setup used to monitor redox changes in YMC-synchronised cultures. **b** Diagram illustrating the coupled metabolic and cell division cycles observed during the YMC. CDC (cell division cycle), HOC (high oxygen consumption), and LOC (low oxygen consumption). **c** Representative traces showing the changes in dissolved oxygen, NAPstar4.3 (NADP redox state), Peredox (NAD redox state), and Hyper7 (H_2_O_2_) during two complete cycles of the YMC (*n* = 2, in which probe dynamics were measured for multiple YMC cycles in two independent YMC-synchronised cultures; Supplementary Fig. 7).

To address this issue, we established continuous yeast cultures^34^ with yeast cells expressing Peredox, NAPstar4.3, or HyPer7^29^. We selected NAPstar4.3 after testing different NAPstar variants for the specific conditions of the fermentor cultures, as this sensor variant showed optimal performance, particularly in terms of allowing fully dynamic measurements, unlike in more nutrient-rich batch cultures where it is almost fully NADP^+^ bound (Fig. 3 and Supplementary Fig. 8). Using a coupled fermentor–fluorimeter setup to continuously monitor probe fluorescence within the cells in the fermentor culture (Fig. 3a, b)^34^, we found periodic oscillations in both NAD and NADP redox states (Fig. 3c). These oscillations were synchronised with fluctuations in oxygen consumption, which are well understood to be coupled to cell division^31,32^. HyPer7 responses confirmed the cell cycle and metabolic cycle-associated H_2_O_2_ cycles that we previously reported, based on roGFP2-Tsa2ΔC_R_ sensor responses^34,35^. Interestingly, our measurements showed that the three different redox species respond independently of each other, are not in equilibrium, and need to be analysed separately. For example, the peak reduction of the NADP pool coincided with the highest H_2_O_2_ levels. Likewise, the reduction and oxidation phases of the NAD and NADP pools occur at different time-points. These experiments highlight the complex and highly dynamic redox landscape of the cell, where individual redox couples, even within a single subcellular compartment, often do not equilibrate with each other in vivo. Those data re-emphasise the fact that there is no such thing as a general cellular redox state and the critical need for the specific empirical measurement of individual redox couples.

### NAPstars reveal NADP redox responses in the plant cytosol to illumination and hypoxia

**Fig. 4:**
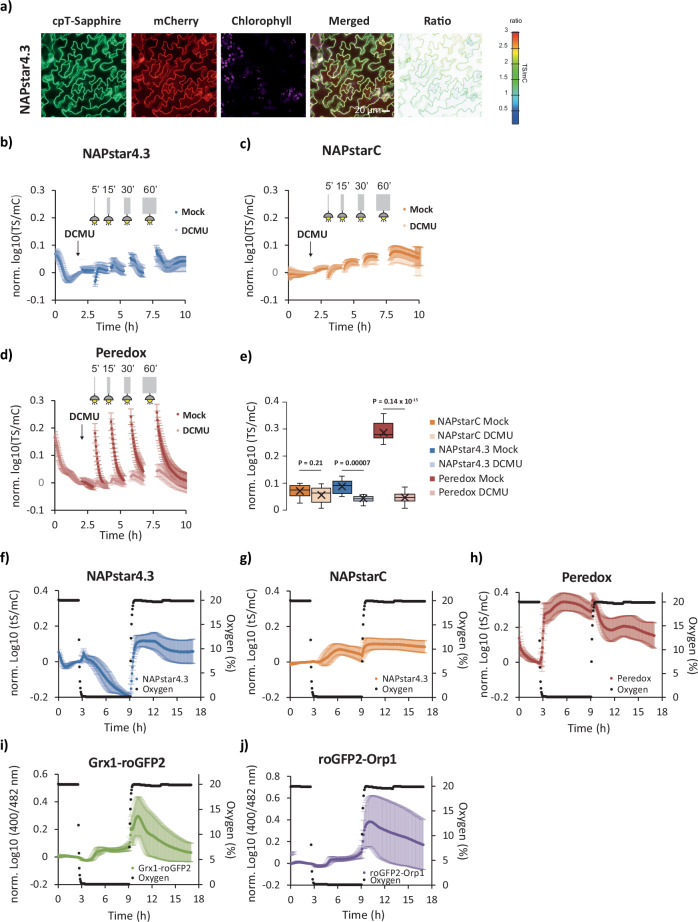
Cytosolic redox dynamics accompany illumination and hypoxia-reoxygenation in plants. **a** Confocal microscopy images of NAPstar4.3 expressed in the cytosol of *Arabidopsis thaliana* plants. Scale bar = 20 µm. Response of NAPstar4.3 (**b**), NAPstarC (**c**) and Peredox (**d**) to the indicated periods of illumination after treatment with a solvent control (Mock) or the photosynthetic inhibitor DCMU (in each panel data presented are the mean ± s.d. based on *n* = 6 leaf discs from six individual plants). **e**, Box and whisker plot, derived from the datasets in **b**–**d**, showing the change in the normalised log10 TS/mC ratio after 60 minutes of illumination. Boxes show the interquartile range, with the middle line defining the median. X, represents the mean values. Whiskers show the minimum and maximum values, excluding outliers. Dots indicate outlier values, which are defined as being 1.5 times the interquartile range above and below the third and first quartile respectively. *P*-values are derived from an unpaired two-tailed Student’s *t*-test. Response of NAPstar4.3 (**f**), NAPstarC (**g**), Peredox (**h**), Grx1-roGFP2 (**i**) and roGFP2-Orp1 (**j**) probes to 6 hours of hypoxia (0.1% oxygen) followed by restoration of normal atmospheric oxygen levels (*n* = 7 for NAPstarC, roGFP2-Orp1 and Grx1-roGFP2, *n* = 8 for Peredox and NAPstar4.3leaf discs taken from 7 or 8 individual plants). In all panels, data are presented as mean ± s.d. normalised to the average value before induction of hypoxia.

Next, we aimed to enable NAPstar-based measurements of the NADP redox state in plants, which plays a crucial role in underpinning photosynthesis and stress responses. Despite its importance in crop improvement, the specific dynamics of compartmentalised NADP redox state have been difficult to assess^36–38^. The few subcellular fractionation datasets that are available have been suggestive of only minor NADP redox changes at dark-light changes in the cytosolic fractions, which appears in stark contrast to pronounced NADP redox dynamics in the chloroplast, and indeed NAD redox dynamics in the cytosol^36–38^. Using *Arabidopsis thaliana* as a model, we generated stable transgenic NAPstar3, NAPstar4.3 and NAPstarC lines with cyto-nuclear sensor expression (Fig. 4a and Supplementary Fig. 9). NAPstar3 and 4.3 were chosen as their different *K*_r(NADPH/NADP+)_ was expected to allow us to cover a broad-range of plausible cytosolic NADP redox states. The steady-state TS/mC ratio of NAPstar3 was higher than for NAPstar4.3 reflecting the lower *K*_r(NADPH/NADP+)_ of NAPstar3. Consistent with our observations in yeast, the difference in NADPH occupancy suggested a close match of the response ranges of both sensor variants with the physiological NADPH:NADP^+^ ratio (Supplementary Fig. 9), which may be estimated at about 0.3.

To assess the in vivo responsiveness of the sensors, we illuminated leaf tissue, reasoning that activation of photosynthesis triggers the exchange of redox equivalents between the chloroplast stroma and the cytosol by different metabolite shuttles (Fig. 4)^39^. Consecutive illumination of discs from mature Arabidopsis leaves, for periods of 5, 15, 30 and 60 min, each of which was followed by fluorimetric time-lapse measurements, revealed a pronounced reduction of the cytosolic NAD pool as monitored by Peredox (Fig. 4d)^23^. The amplitude of NAD reduction increased slightly with the duration of illumination (Fig. 4d). In contrast, NAPstar4.3 did not exhibit cytosolic NADP reduction after 5 and 15 min of illumination (Fig. 4b). A slight, but reproducible sensor response beyond that of the NAPStarC control was observable only after longer illumination periods, i.e. 30 and 60 min, indicating a degree of cytosolic NADP reduction (Fig. 4b). The response was decreased, albeit not completely abolished by the photosynthetic inhibitor 3-(3,4-dichlorophenyl)-1,1-dimethylurea (DCMU) (Fig. 4b–e), indicating a contribution of photosynthetic electron transport to the response. Overall, the dynamics observed for the NAPstars were minor as compared to the response of Peredox, confirming the respective specificity of the two sensors for NADP and NAD redox status in the plant cytosol and validating previous indications from subcellular fractionation measurements that only minor NADP redox changes occur in the cytosol at the onset of illumination^36–38^. The NAPstarC control also showed changes, indicating an NADP-independent effect in illuminated leaf tissue, which we did not observe in the other models. These changes may however be corrected for by subtraction (not performed here in the interest of full data transparency) (Fig. 4b,c). Based on the observation that NAPstar4.3 allows dynamic measurements of both reductive and oxidative changes in the cytosolic NADP pool, we selected NAPstar4.3 as suitable sensor variant for further measurements in plant leaf tissue.

We next explored NADP redox dynamics in response to hypoxia stress, which causes a cellular redox crisis. Hypoxia-dependent inhibition of respiration leads to NAD reduction, as previously observed in living leaf tissue^40^. The impact of hypoxia on cytosolic NADP redox dynamics has remained less clear since lack of oxygen as an electron sink causes metabolic reduction but also boosts H_2_O_2_ production and glutathione oxidation in different phases of the hypoxic episode^41^. Lowering oxygen levels to 5%, 1% and 0.1% for 6 h before reoxygenation to ambient levels confirmed rapid and reversible NAD reduction in Arabidopsis leaf tissue at 1 and 0.1% oxygen (Fig. 4f–j and Supplementary Fig. 10). Interestingly, the NADP redox state, as monitored by NAPstar 4.3, responded oppositely to the NAD redox state. After a lag phase, the NADP pool was gradually oxidised. At the technical level this response validates the strict specificity of NAPstar4.3 for the NADP, and not NAD, redox state *in planta*. At the physiological level, it shows that the redox dynamics of the NADP and NAD pools are strictly independent in the plant cytosol, which is in line with the absence of any transhydrogenase gene homologue in the plant genome.

We next hypothesised that NADP oxidation during hypoxia may be due to NADPH consumption by the antioxidant machinery which is not matched by the rate of resupply due to limitations in flux through central metabolism. To test this hypothesis, we also measured glutathione redox potential and H_2_O_2_ dynamics using Grx1-roGFP2 and roGFP2-Orp1, respectively. Both sensors showed increased oxidation during 1% and 0.1% hypoxia, which correlated with NADP oxidation, suggesting efficient equilibration between NADP and glutathione redox status via glutathione reductase in the plant cytosol. This correlation was lost upon reoxygenation, however, as the NADP pool was rapidly reduced, while both Grx1-roGFP2 and roGFP2-Orp1 reported a burst in oxidation (Fig. 4f–j). This observation might be explained by a burst of H_2_O_2_ production during reoxygenation, while metabolic NADP reduction is efficiently restored. The reduction of the NADP pool suggests that the electron influx into the NADP pool by metabolism more than compensates for the electron efflux to the antioxidant systems, and that glutathione reductase activity, catalysing the reduction of glutathione from NADPH, is limiting under the specific conditions of reoxygenation after hypoxia.

We reasoned that the diametrically opposed redox responses of the NADP and glutathione pools to reoxygenation, i.e. NADP reduction and glutathione oxidation, represented an opportunity to benchmark the recently introduced biosensor NERNST against NAPstars and roGFP2-Grx1 probes in vivo. NERNST is based on a redox-sensitive GFP (roGFP2), which is known to respond sensitively to changes in the glutathione redox state (*E*_GSH_) in a reaction efficiently catalysed by endogenous glutaredoxins in most cell compartments, including the plant cytosol^19–22^. This raises the crucial question of whether NERNST truly reports the NADP redox state in vivo or whether its specificity is compromised by catalysed equilibration with *E*_GSH_. We thus repeated the hypoxia-reoxygenation experiments in leaves with NAPstar4.3, NAPstarC, Peredox, roGFP2-Grx1 and NERNST sensors (Supplementary Fig. 11) The NERNST response was almost indistinguishable from the roGFP2-Grx1 response (Supplementary Fig. 11c,d) consistent with NERNST responding to changes in *E*_GSH_ in the *Arabidopsis* cytosol. Since high glutaredoxin activity, through several different glutaredoxin isoforms, is common in the cytosol of plants in general, our data indicates that NERNST is not an NADP redox state sensor in plants and functions primarily as an *E*_GSH_ reporter.

### The glutathione pathway mediates high capacity NADPH-dependent antioxidative electron flux

Having established the functionality of NAPstars in yeast, mammalian cells and plants, we next sought to address a longstanding question in redox biology, namely, the role of glutathione in cellular antioxidative responses. Although glutathione is often loosely referred to as a key cellular antioxidant, its mechanistic roles within cells have been remarkably difficult to pin down and a matter of longstanding debate. Importantly, the thioredoxin pathway is considered to be the primary reductive system, with glutathione seen as having a backup or auxiliary role, particularly in yeast and mammalian cells^42–44^.

Upon exposure to exogenous oxidants, such as H_2_O_2_ or diamide, there is a strongly increased demand for NADPH. Both the thioredoxin reductase/thioredoxin and glutathione reductase/glutathione/glutaredoxin pathways (ascorbate-glutathione cycle in plants)^45^ use electrons from NADPH to reduce cellular disulfide bonds and certain reactive oxygen and reactive nitrogen species. Nonetheless, monitoring the relative flux of electrons through these two pathways in vivo under pro-oxidative conditions has proven challenging. To address the question of the relative contribution to antioxidant electron flux we made use of genetic or chemical inhibition of the glutathione and thioredoxin-dependent pathways and monitored the cytosolic NADP redox dynamics in response to exogenous oxidants in living cells and tissues.

**Fig. 5:**
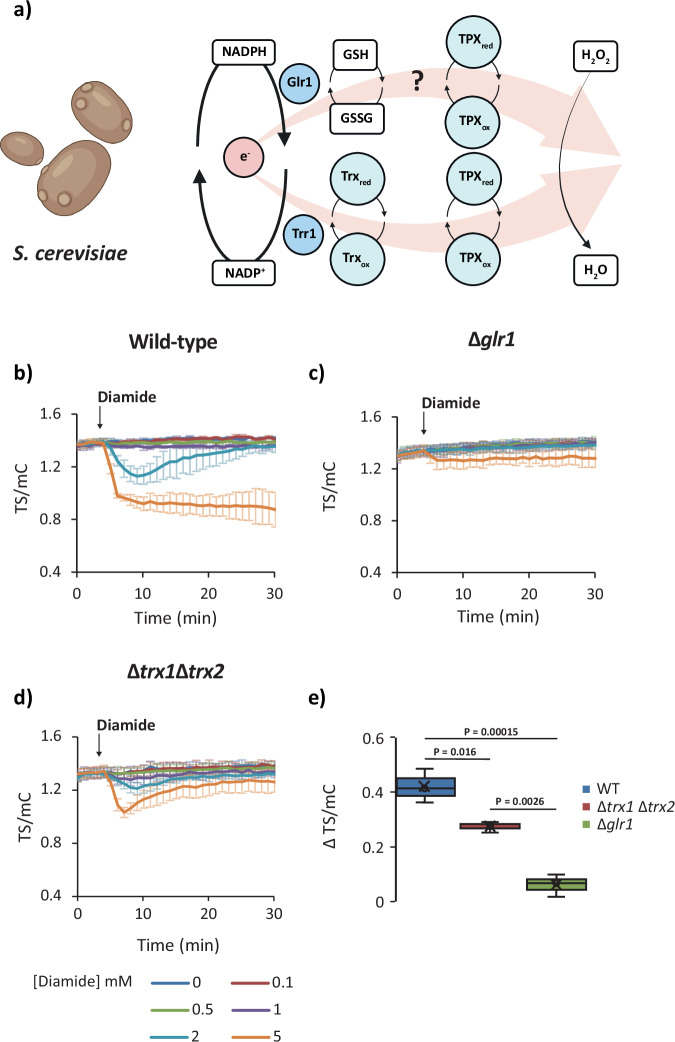
Glutathione reductase deletion protects against diamide-induced NADP oxidation in yeast. **a** Cartoon showing two possible pathways by which electrons from NADPH can be transferred to H_2_O_2_ in the yeast cytosol, GR glutathione reductase, TPX thioredoxin peroxidase, TRX thioredoxin. The ‘?’ indicates a lack of information concerning the relative importance of glutathione as a reductant for thiol peroxidases in the yeast cytosol. Created with BioRender.com. Response of a cytosolic NAPstar3 probe to exogenous diamide at the indicated concentrations in wild-type (**b**), Δ*glr1* (**c**), and Δ*trx1*Δ*trx2* (**d**) cells (*n* = 3 measurements made with cells derived from independent cultures). **e**, Box and whisker plot, derived from the datasets presented in **b**–**d**, showing the change in TS/mC before and after treatment with 5 mM diamide. Boxes show the interquartile range, with the middle line defining the median. X, represents the mean values. Whiskers show the minimum and maximum values, excluding outliers. Dots indicate outlier values, which are defined as being 1.5 times the interquartile range above and below the third and first quartile respectively. *P*-values are derived from a one-sided ANOVA test. In all panels, data are presented as mean ± s.d. Identical experimental settings were used for all panels allowing for direct comparison of TS/mC between datasets.

First, we examined yeast cells. We monitored the response of NAPstar3, NAPstarC, and PeredoxDS probes expressed in wild-type cells, in cells deleted for the *GLR1* gene (encoding glutathione reductase), and in cells deleted for *TRX1* and *TRX2* (encoding the two cytosolic thioredoxins). The probe responses in all cells were monitored in response to the addition of exogenous diamide at concentrations ranging from 0–5 mM (Fig. 5a–e). Diamide was chosen instead of H_2_O_2_ due to the extremely small deflections in cytosolic NADPH redox homoostasis detected in yeast in response to peroxides (Fig. 2 and Supplementary Figs. 6 and 7). In wild-type cells, we observed a strong oxidation of the cytosolic NADP pool upon treatment with 2 and 5 mM diamide (Fig. 5b). Intriguingly however, in Δ*glr1* cells, we observed almost no detectable NADP oxidation at all, except for a slight response of the NAPstar3 probe to 5 mM diamide (Fig. 5c,e). Deletion of both cytosolic thioredoxins also led to less severe NADP oxidation following diamide treatment in comparison to that observed in wild-type cells (Fig. 5d, e). However, the change of the NAPstar3 TS/mC ratio was much greater than that observed in Δ*glr1* cells (Fig. 5e). The response of PeredoxDS was similar in wild-type, Δ*glr1* and Δ*trx1*Δ*trx2* cells, while NAPstarC showed no response in all cases (Supplementary Fig. 12). Our results indicate that genetic impairment of both the thioredoxin and glutathione-dependent pathways can hinder the consumption of NADPH upon pro-oxidative challenges in the yeast cytosol, but the impact of blocking the glutathione pathway is more pronounced.

We again saw a chance to test the response of the NERNST sensor. The lack of coupling between the NADP redox status and *E*_GSH_ in Δ*glr1* cells, provides a powerful genetic system to separate NADP and *E*_GSH_-dependent responses. During acute oxidative challenge, *E*_GSH_ is expected to respond more strongly (and recover less quickly) in Δ*glr1* cells, whille NADP redox status is expected to respond less strongly (and recover more quickly). Compared to wild-type, NERNST responses to both H_2_O_2_ and diamide were strongly increased in Δ*glr1* cells, suggesting that *E*_GSH_ dominates the NERNST response in the cytosol of yeast cells. Consistently, the NERNST response was very similar to roGFP2-Grx1 responses. We performed a systematic, parallelised comparison of NERNST responses with the NAPstarC, NAPstar3 and NAPstar6 sensors, as well as roGFP2 and roGFP2-Grx1, in WT, Δ*glr1*, Δ*grx1*Δ*grx2* and Δ*glr1*Δ*grx1*Δ*grx2* yeast cells, with H_2_O_2,_ diamide and *t*-BuOOH as three different oxidants (Supplementary Figs. 13–15). Under any tested condition, the NERNST responses differed markedly from those of NAPstars. Strikingly, we observed an almost exact match between NERNST and free roGFP2 responses.

**Fig. 6:**
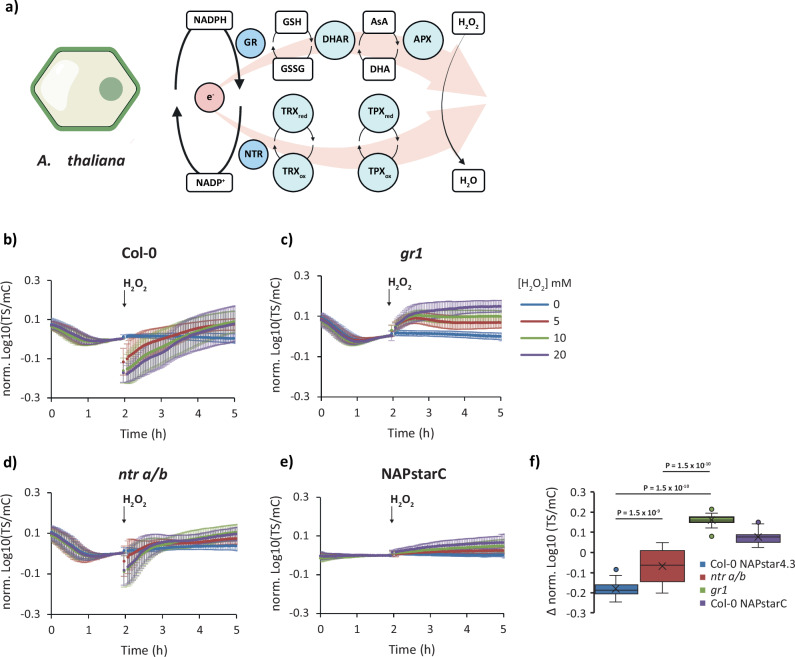
Glutathione reductase deletion protects against H_2_O_2_-induced NADP oxidation in plants. **a** Cartoon showing two possible pathways by which electrons from NADPH can be transferred to H_2_O_2_ in the plant cytosol, AsA ascorbic acid, APX ascorbate peroxidase, DHAR dihydroascorbate reductase, GR glutathione reductase, TPX thiol peroxidase, TRX thioredoxin. Created with BioRender.com. Response of a cytosolic NAPstar4.3 probe to exogenous H_2_O_2_ at the indicated concentrations in wild-type (Col-0) plants (**b**), *gr1* (**c**), *ntr a/b* (**d**) plants, and the control construct NAPstarC in wild-type plants (**e**) (*n* = 6, except for NAPstar4.3 with 10 mM H_2_O_2_ in *ntr a*/*b* where *n* = 5, leaf discs from 6 (or 5) individual plants). **f**, Box and whisker plot, derived from the datasets presented in **b–d**, showing the change in TS/mC before and after treatment with 20 mM H_2_O_2_. Boxes show the interquartile range, with the middle line defining the median. ‘X’, represents the mean values. Whiskers show the minimum and maximum values, excluding outliers. Dots indicate outlier values, which are defined as being 1.5 times the interquartile range above and below the third and first quartile respectively. *P*-values are derived from a one-sided ANOVA test. In all panels, data are presented as mean ± s.d. normalised to the average value before addition of H_2_O_2_.

We next asked whether the different impacts of impairing the glutathione and thioredoxin pathways are specific to yeast or also hold true in other eukaryotic systems. Therefore, we monitored the response of NAPstar4.3 in wild-type *A. thaliana* plants, as well as in plants lacking the two NADPH-dependent thioredoxin reductases NTR A and NTR B (*ntr a/b*) or glutathione reductase GR1 (*gr1*) (Fig. 6 and Supplementary Fig. 16). We observed an H_2_O_2_ concentration-dependent NADPH consumption in leaf discs from wild-type plants, which was similar to that in *ntr a/b* plants, but absent in *gr1* plants. The dominant role of the glutathione system over the thioredoxin system in mediating antioxidant electron flux *in planta* is consistent with our observations in yeast, and the concept of the ascorbate-glutathione cycle as the dominant route of NADP-dependent antioxidant electron flux in plants.

**Fig. 7:**
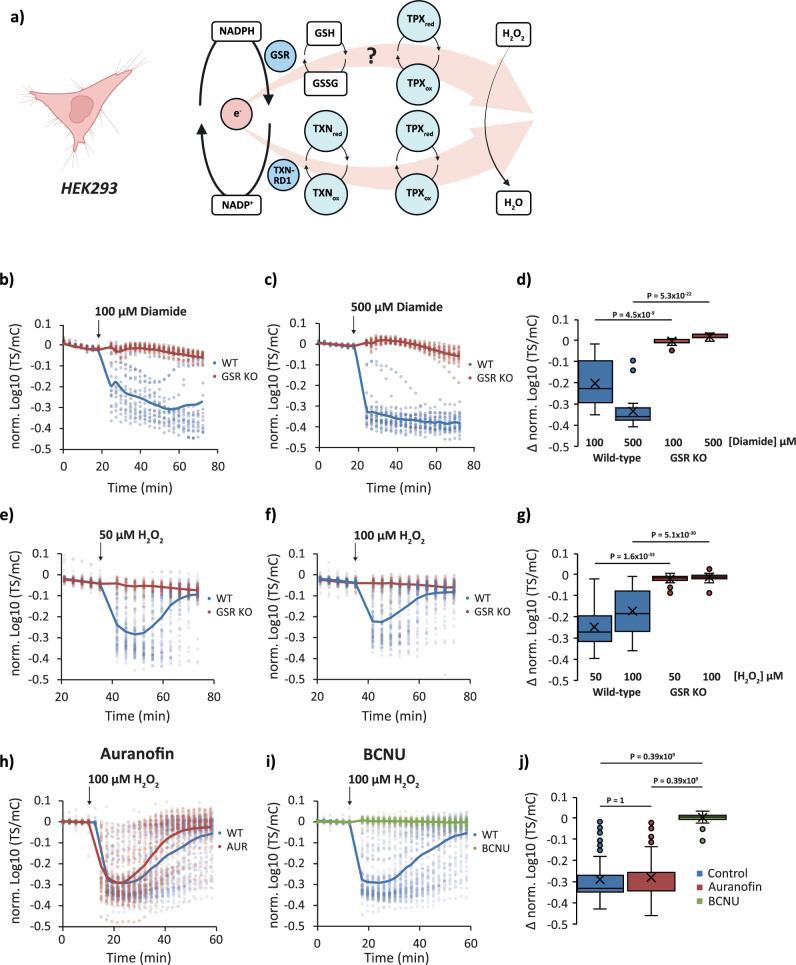
Glutathione reductase deletion protects against H_2_O_2_-induced NADP oxidation in HEK293 cells. **a** Cartoon showing two possible pathways by which electrons from NADPH can be transferred to H_2_O_2_ in the mammalian cell cytosol, GSR glutathione reductase, TPX thioredoxin peroxidase, TRX thioredoxin, TXNRD1 thioredoxin reductase. The ‘?’ indicates a lack of information concerning the relative importance of glutathione as a reductant for thiol peroxidases in the mammalian cell cytosol. Created with BioRender.com. Response of NAPstar3b in the cytosol of wild-type and glutathione reductase deleted (GSR KO) cells to 100 µM diamide (**b**) (*n* = 17 or 18 individual cells for wild-type and GSR KO, respectively, measured in two separate experimental repeats) or 500 µM diamide (**c**) (*n* = 22 or 19 individual cells for wild-type and GSR KO, respectively, measured in two separate experimental repeats. **d** Box and whisker plot showing the change in TS/mC after treatments in **b** and **c.** Response of NAPstar3b expressed in the cytosol of wild-type and GSR KO cells to 50 µM H_2_O_2_ (**e**) (*n* = 103 or 46 individual cells for wild-type and GSR KO respectively, measured in four and three separate experimental repeats) or 100 µM H_2_O_2_ (**f**) (*n* = 98 or 76 individual cells for wild-type and GSR KO, respectively, measured in four and five separate experimental repeats. **g** Box and whisker plot showing the change in TS/mC after treatments in **e** and **f.** Response of NAPstar3b in the cytosol of HEK293 cells, treated either with a vehicle control, the thioredoxin reductase inhibitor auranofin (**h**) or the glutathione reductase inhibitor 1,3-bis(2-chloroethyl)−1-nitrosourea (BCNU) (**i**) (*n* = 85, 97, 79 individual cells for control, auranofin and BCNU treatments respectively, monitored in the course of 3 experimental repeats). **j** Box and whisker plot showing the change in TS/mC after treatments in **h** and **i**. Data were normalised to the average value at the beginning of the measurement. For all box and whisker plots, boxes show the interquartile range, with the middle line defining the median. ‘X’, represents the mean values. Whiskers show the minimum and maximum values, excluding outliers. Dots indicate outlier values, which are defined as being 1.5 times the interquartile range above and below the third and first quartile respectively. *P*-values in panels **d**. and **g**, were determined by unpaired two-tailed Student’s *t*-tests. *P*-values in panel **j**. were determined by a one-sided ANOVA test.

Finally, we asked whether this principle may be generalised and tested the impact of glutathione reductase deletion on NADP redox responses in mammalian cells lines (Fig. 7 and Supplementary Fig. 17). To this end, we monitored the response of NAPstar3b in HEK293 cells and in HEK293 cells with a CRISPR-Cas9-mediated disruption of GSR (encoding glutathione reductase). The response of NAPstar3b in several hundred individual cells was imaged using a plate-reader-based fluorescence microscopy setup. Cells were treated with diamide at 100 or 500 µM (Fig. 7b–d) or with H_2_O_2_ at 50 or 100 µM (Fig. 7e–g). Consistent with our observations in yeast and plants, we observed cytosolic NADP oxidation in control cells in response to both H_2_O_2_ and diamide. We observed no detectable NAPstar3b response to either oxidant in GSR KO cells. Finally, we tested the impact of chemical inhibition of glutathione reductase and thioredoxin reductase using 1,3-bis-(2-chlorethyl)-l-nitroso-urea (BCNU) and auranofin, respectively (Fig. 7h–j). In HeLa cells treated with auranofin for 60 min prior to the addition of 100 µM H_2_O_2_, we observed no difference in NAPstar3b response compared to untreated control cells. In contrast, in cells pretreated for 60 min with BCNU prior to H_2_O_2_ addition, we observed no NAPstar3b response at all, consistent with our observations of genetic disruption of glutathione reductase in yeast, plants, and HEK293 cells. In summary, we conclude that the glutathione system is the dominant mediator of antioxidative electron flux under pronounced pro-oxidative conditions across different eukaryotic kingdoms.

## Discussion

Here we report the development of the NAPstar family of genetically encoded fluorescent biosensors for in-cell monitoring of the NADP redox state. The NAPstars are functional in yeast, plants and mammalian cells. Importantly, unlike other currently available NADP sensors, including iNaps and Apollo-NADP^+^^15,16^, NAPstars respond to changes in both NADPH and NADP^+^ and are bona fide probes for the NADP redox state (Fig. 1**;** Supplementary Figs. 1–4). NAPstars exhibit several other advantages over currently available NADP sensors including a limted pH sensitivity (Supplementary Fig. 3), which affords the possibility to monitor more reliably in situations where pH can respond readily to environmental changes, for example in the yeast cytosol^46^. This advantage is shared with iNap-mCherry variants for monitoring NADPH concentration changes^47,48^. NAPstars afford readily accessible monitoring by fluorescence spectroscopy, fluorescence microscopy and fluorescence lifetime imaging. Furthermore, NAPstars contain both Rex domains necessary for NADPH or NADP^+^ binding within one probe molecule. NAPstars are thus self-contained probes and unlike iNap sensors, they do not rely on probe dimerisation to function^15^. This greatly increases the possibility for engineering probe properties such as NADPH or NADP^+^ binding affinity and nucleotide specificity by creating NAPstar probes composed of two differently mutated Rex domains. The mixed Rex domain NAPstar4.3 for example has no detectable binding affinity for NADH and has a *K*_dNADPH_ ≈ 43 µM and a *K*_r(NADPH/NADP+)_ ≈ 0.28, which is approximately one order of magnitude lower than that of other NAPstar sensors (Fig. 1) and allows fully dynamic measurement of NADP oxidation and reduction in the yeast cytosol during the yeast metabolic cycle and in the plant cytosol during illumination and hypoxia (Figs. 3 and 4). The self-contained nature of NAPstars also allows them to function independently of their expression level whereas probes that rely on dimerisation may potentially have issues at lower expression level. We also demonstrated that NAPstars are amenable to FLIM measurement, with a large change in fluorescence lifetime between the NADPH and NADP^+^-bound states, as exemplified by NAPstar4.3 (Supplementary Fig. 5). Finally, in common with other NAD(P) probes, but unlike genetically encoded probes for redox species such as H_2_O_2_^6,29,35,49^ or glutathione^19^, NAPstars *bind* their target redox species fully reversibly but do not *react* with it. In vivo, NAPstars are one amongst many other endogenous NADP-binding proteins decreasing the likelihood that NAPstars perturb the NADP pool to any considerable extent.

NERNST is a recently published sensor for measuring the NADP redox state^18^. NERNST is based on a genetic fusion between redox-sensitive green fluorescent protein 2 (roGFP2)^18^ and NADPH-thioredoxin reductase C (NTRC) from rice (*Oryza sativa*). Although this is an elegant concept, there are legitimate reasons to question the specificity of NERNST in many biological contexts. RoGFP2 has been shown to rapidly equilibrate with the glutathione redox couple in a reaction catalysed by endogenous redox-active glutaredoxins^19,20,22^. An interaction with glutathione/glutaredoxin has been observed in all roGFP-based sensors generated to date^19,35,49–53^ and it is unclear why this interaction should not also occur in the context of the NERNST probe. Here, we employed two different experimental settings in which glutathione and NADP redox responses are diametrically opposed, to test whether NERNST responds predominantly to NADP or glutathione redox changes. In both experiments, i.e. the differential response of the glutathione and NADP redox states to oxidative challenge in Δ*glr1* and wild-type yeast and during reoxygenation in plants, the behaviour of NERNST closely mirrored that of the well-established *E*_GSH_ sensor, roGFP2-Grx1, and particularly that of a free roGFP2, which can equilibrate with the glutathione redox couple via the action of endogenous glutaredoxins, and was opposite to that of the NAPstars. We thus conclude that NERNST predominantly responds to changes in *E*_GSH_ in vivo. Therefore, NERNST is not an NADP redox state sensor in the cellular contexts assessed here, and this conclusion can be plausibly extrapolated to most cellular systems, where endogenous cytosolic glutaredoxin activity is typically high.

As with every methodological approach, NAPstars also have limitations of which it is important the user is aware. Firstly, like most genetically encoded sensors, NAPstars are well suited for monitoring relative changes in NADP redox state, but typically does not allow absolute quantification of NADP redox state or changes thereof. Furthermore, the TS/mC ratio will be affected by factors including measurement equipment and optical settings, cannot be directly correlated with NADP redox state in vivo, and cannot be directly compared between different experiments unless identical measurement conditions are employed. Quantitative measurements may be possible in the future if robust in vivo calibration procedures can be developed. As with any genetically encoded probe, NAPstars require genetic manipulation of the organism in which the probe is intended to be used. Moreover, NAPstars are relatively large constructs, which may limit their targetability or usability in some subcellular locations, such as experienced for targeting Peredox to the mitochondrial matrix. Finally, it is important to note that NAPstars monitor the soluble pools of NADPH and NADP^+^, they do not ‘see’ protein-bound NADP, which may constitute a significant fraction of total NADP in some situations^36^.

The cytosolic NADP redox state is typically considered to be highly reduced, with NADPH/NADP^+^ ratios of 50–100:1 being standard textbook values^54,55^. However, a very wide-range of values for cytosolic NADPH/NADP^+^ has been reported in the literature^36,56–58^. In plants for example, whole tissue NADPH:NADP^+^ ratios are typically reported to be in the range of 1:1 to 2:1, which is of a similar magnitude as that reported by NAPstar sensors for the cytosol NADPH:NADP^+^ ratio, please also see our recent review for a summary of the situation in plants^36^. In other organisms, including yeast and mammalian cells, literature values for whole cell or tissue lysate NADPH:NADP^+^ values vary widely. For example, Pollak and colleagues^59^ reported an NADPH:NADP^+^ ratio of ~1:4 using an HPLC-based approach on HEK293 cell lysate which would be fully consistent with the NAPstar measurements. In contrast, Sallin and co-workers, recently reported a NADPH:NADP^+^ of ~75:1 in the ‘cytosol’ of HeLa cells using a TCSPS-FLIM approach^60^, whilst a value of 244:1 was reported in the liver of mouse cells^61^. Therefore, reported ratios differ by almost three orders of magnitude. This raises questions regarding the actual cytosolic NADP redox state and how variable it might be under different conditions or in different organisms. The NAPstars have *K*_r(NADPH/NADP+)_ values ranging from 0.0006–0.28. This means that they can respond to changes in NADPH/NADP^+^ ranging from about 1:1000 to approximately 5:1. The fact that NAPstars3, 3b, and 4.3 allow for dynamic measurements in vivo, i.e. their binding status can shift towards more NADP^+^ but also towards more NADPH, supports the conclusion that the cytosolic NADP redox state lies within the sensitivity range of those sensor variants (Fig. 1C) and thus is more oxidised than often considered. As NAPstar4.3 was best suited for fully dynamic measurements in plant leaves, and in glucose-limited continuous yeast cultures, we can conclude that cytosolic NADPH/NADP^+^ in these systems is in the range of approximately 1:10 to approximately 5:1, which would give cytosolic *E*_NADP_ ranging from −290 mV to –340 mV. This is intriguing as numerous measurements of the cytosolic *E*_GSH_ report similar values, typically in the range of −300 mV to –320 mV^62^. The apparent overlap in redox potentials could suggest that the cytosolic glutathione and NADP redox couples are able to rapidly equilibrate with each other, for example through the action of glutathione reductase. Supporting this hypothesis, we observed that despite the more oxidised than expected cytosolic NADP redox state, it seems to be coupled with a remarkable robustness to perturbation by exogenous oxidants in both yeast and mammalian cells. This robustness was abolished in the Δ*zwf1* yeast background where the oxidative pentose phosphate pathway as the major NADPH source of the cytosol is impaired and cannot be regulated and in mammalian cells starved for glucose. Those results demonstrate that regulation of central metabolism is responsible for the observed robustness of cytosolic NADP redox status, which is itself a prerequisite for the previously-described robustness of the glutathione redox status. These findings are strikingly similar to our previous observations on the robustness of cytosolic glutathione redox homoeostasis^21,63^ and raise the exciting possibility that glutathione reductase may facilitate the rapid equilibration between the glutathione and NADP redox couples. It will be fascinating to explore the in vivo crosstalk of those key redox couples further in the future, and to understand situations in which the glutathione and NADP redox couples are clearly not coupled, including those documented in this work for re-oxygenation after hypoxia in Arabidopsis tissue (Fig. 4f–j). While under hypoxia both NADP and glutathione showed oxidation with matching dynamics, re-oxygenation caused pronounced further oxidation of glutathione while NADP recovered to a more reduced state. Both behaviours can be accounted for individually, i.e. glutathione is likely to be oxidised by the burst of ROS released from over-reduced electron transport components and metabolic NADP reduction can re-commence due to oxygen-dependent metabolic flux through the relevant enzyme systems such as those of the cytosolic oxidative pentose phosphate pathway. However, the inverse redox dynamics of the two pools indicate that the coupling between the NADP redox state and *E*_GSH_ is disrupted and equilibration via GR1 is limiting. Such a limitation during the ROS burst may either be accounted for by a very large demand for antioxidant electron flux, or by inhibition of GR1 during the ROS burst. Since the capacity of GR1 in the Arabidopsis cytosol is large and far from limiting under normal growth conditions^63^, inhibition of GR1 by (hyper)oxidation of catalytic or regulatory cysteine residues provide a plausible hypothesis^64^ either as a result of oxidative damage or as a mechanism to safeguard NADP redox status, similar to what has previously been observed for peroxiredoxins^50,65^.

In the yeast and mammalian cytosol, the thioredoxin pathway is considered to be the major reductive pathway with the glutaredoxin/glutathione system having only a backup or auxiliary role^42,44^. In the plant cytosol there is evidence for a major antioxidant electron flux through the glutathione system, specifically via the ascorbate-glutathione cycle^45,66^. Furthermore, a recent elegant in vitro reconstitution approach supported a major role for glutathione in Arabidopsis cytosolic peroxiredoxin reduction^67^. Nonetheless, even in plants, conclusive direct in vivo evidence of the partitioning of NADPH-dependent reductive flux through the thioredoxin and glutathione systems is lacking.

It was proposed that the only essential function of glutathione is in Fe-S cluster biogenesis^42^. Nonetheless, only micromolar amounts of glutathione are required for Fe-S cluster biogenesis and the biological reason for the millimolar concentrations of glutathione present in cells across the eukaryotic kingdom remains unclear^68^. Furthermore, indirect evidence from several studies supports the important role of glutathione as a reductant, especially in response to acute oxidant challenges. For example, GSSG is readily produced in cells in response to exogenous oxidants, supporting the concept that GSH acts as a reductant in these situations^21,66^. Cellular GSSG accumulation is limited by the fact that cells employ multiple redundant reductive pathways to reduce cytosolic GSSG or to actively export GSSG to the extracellular environment, for example by MRP1 in mammalian cells or to alternative subcellular compartments, for example by Ycf1 in yeast^21,69^. Moreover, GSH has been shown to reduce typical 2-Cys peroxiredoxins, including human PRDX2^70^ and Arabidopsis PRXIIB, C, and D^67^. The relative importance of the glutathione system compared to the more important thioredoxin system may be further amplified under acute oxidative challenge as thioredoxins or thioredoxin reductases may readily become limiting and/or oxidised and thus inactive^71^, which would strongly limit the capacity of the thioredoxin pathway to act as a conduit of antioxidative electron flux.

Here we used NAPstar sensors, in yeast, plants, and mammalian cells in combination with genetic and chemical inhibition to re-visit the question of the relative importance of the glutathione and thioredoxin systems as mediators of antioxidative electron flux during acute oxidative challenges. In yeast and plants, we observed almost no NADPH consumption upon oxidative challenge with H_2_O_2_ or diamide in the absence of a cytosolic glutathione reductase (Figs. 5 and 6). Similarly, in mammalian cells, deletion of glutathione reductase or chemical inhibition with BCNU, led to almost complete loss of NADPH consumption with both H_2_O_2_ and diamide (Fig. 7). In contrast, genetic or chemical ablation of the thioredoxin pathway, had a much smaller effect in all three organisms. We interpret these results to mean that the glutathione system acts throughout the eukaryotic kingdom as the major mediator of antioxidative electron flux in response to acute oxidative challenge. It will be exciting in the future to utilise NAPstar sensors to investigate the relative importance of the glutathione and thioredoxin pathways under conditions of endogenously generated oxidative challenge, such as frequently occurring stress situations.

In conclusion, we have developed and extensively characterised the NAPstar family, which are bona fide biosensors of the NADP redox state. NAPstars are bright and specific NADP redox state sensors that can be measured either by standard fluorescence spectroscopy-based approaches or by FLIM. We have used NAPstars to explore NADP biology in yeast, plant and mammalian systems, finding an unexpected dominance of the glutathione system as a mediator of antioxidative electron flux that appears to be conserved in eukaryotic evolution. We are confident that NAPstars will drive the discovery and understanding of a broad range of novel NADP biology and its synthetic rewiring in the future.

## Methods

### Plasmid construction

Amino acid mutations to switch Rex-domain specificity from NAD to NADP were selected based on a previous screen^15^. All constructs and gene sequences used in this study were either commercially synthesised, generated by standard molecular cloning approaches or were generated in previous studies (Supplementary Information; Supplementary Tables 1–5). Primer sequences are provided in Supplementary Table 6. All NAPstar amino acid sequences are listed in the Supplementary Information. All sequences were confirmed by commercial sequencing (Eurofins Genomics, Ebersberg, Germany).

#### Cloning and site-directed mutagenesis

NAPstar sequences for plant expression were commercially synthesised with codons optimised for plant expression (GenScript Biotech, Rijswijk, Netherlands) and inserted into pDONR207 (LIFE Technologies, Carlsbad, CA, United States) (Supplementary Table 1). The NAPstar3b variant was generated by site-directed mutagenesis on the pDONR207 NAPstar3 plasmid using the primer pair Pr1/Pr2 (Supplementary Table 6). For plant expression all constructs were subcloned into pSS02 (derivative of pMDC32^23,72^;) using gateway cloning (Supplementary Table 2). Plant NAPstar sequences exhibit a pre-existing *HindIII* restriction site within the cpT-Sapphire (TS) domain. Using this restriction site, together with an *ApaI* restriction site in the backbone of pDONR207, the N-terminal Rex domain of NAPstar4 was fused to the second, C-terminal, Rex domain of NAPstar3 to generate NAPstar4.3. For bacterial expression, all plant NAPstar sequences were subcloned by gateway cloning^73^ into pETG10a (Invitrogen, Carlsbad, CA, United States) (Supplementary Table 3).

All coding sequences for NAPstars, Peredox, HyPer7 and NERNST were codon optimised for expression in *Saccharomyces cerevisiae*, synthesised and delivered in a pUC57 plasmid (GenScript Biotech, Rijswijk, Netherlands). For yeast expression, all coding sequences were subcloned into an empty p413TEF plasmid^74^ using *XbaI* and *XhoI* restriction sites (Supplementary Table 5). A pre-existing *ClaI* restriction site in the TS sequence was utilised to fuse the first, N-terminal, Rex domain of NAPstar4 to the second, C-terminal, Rex domain of NAPstar3 to generate the NAPstar4.3 construct. The p413TEF roGFP2-Grx1 plasmid was generated by subcloning from a p415TEF roGFP2-Grx1 plasmid. The PeredoxDS construct was obtained after two rounds of mutagenesis on the p413TEF Peredox plasmid using primer pairs Pr3/Pr4 and Pr5/Pr6 (Supplementary Table 6). Site-directed mutagenesis was performed using a standard PCR-based protocol with S7 Fusion Polymerase (Biozym Scientific GmbH, Hessisch Oldendor, Germany). Methylated template DNA was digested using *DpnI* (NEB, Ipswich, MA, United States), before heat-shock transformation into chemically competent *E. coli* Top10 cells.

Codon optimised sequences of NAPstar3b and HyPer7 for expression in mammalian cells were commercially synthesised (Supplementary Table 4) (GenScript Biotech, Rijswijk, Netherlands) and delivered in pcDNA3.1(+) plasmids.

### AlphaFold2 structure prediction of monomeric NapStar

Structural prediction was performed with AlphaFold2 with ColabFold. The following script was used for running the structural prediction process: colabfold_batch --model-type AlphaFold2--num-recycle 48 --amber --use-gpu-relax)^75^ using the amino acid sequence of NAPstar3 as input (Supplementary List 1). In the prediction process, 48 recycling steps were employed, serving as iterations where the model fine-tuned its predictions to enhance accuracy. The refinement stage utilised AMBER (Assisted Model Building with Energy Refinement), a force field commonly used in molecular dynamics simulations. Additionally, a relaxation process was implemented, optimising the predicted structures further to attain more realistic and energetically favourable conformations. Visualisation was performed using PyMol software (https://pymol.org).

### Expression and purification of recombinant proteins

Protein expression and purification were performed as previously described^23,76^ with the following modifications. Proteins were isolated from either an *E. coli* BL21 (DE3) ArcticExpress strain or from an *E. coli* Rosetta 2 (DE3) strain expressing pETG10a NAPstar or pETG10a Peredox. Isopropyl β-D-1-thiogalaytopyranoside (IPTG) was added at a concentration of 0.5 mM overnight induction at 20 °C. Bacterial pellets were obtained by centrifugation for 15 min at 3220 *g* and pellets were resuspended in 50 mM Tris-HCl, 100 mM NaCl, 0.5 mM MgCl_2_ (pH 7.5). Cell lysates were centrifuged at 12,100 *g* for 15 min at 4 °C. Clarified lysates were incubated with Ni-NTA beads. Beads were washed with 20 mM and 40 mM imidazole steps and proteins were eluted using 250 mM imidazole in 50 mM Tris-HCl, 100 mM NaCl, 0.5 mM MgCl_2_ (pH 7.5).

### In vitro characterisation of NAPstar sensors

In vitro assays were performed in 50 mM Tris-HCl, 100 mM NaCl, 0.5 mM MgCl_2_ at pH 7.5, except for pH titration and enzyme-coupled assays experiments. For all in vitro assays the NAPstar sensor protein was quantified by Bradford assay^77^ and the sensor protein was used at a final concentration of 240–480 nM.

In vitro assays were performed using NUNC 96-well plates (VWR International GMBH, Darmstadt, Germany) and a CLARIOstar plate reader (BMG Labtech, Ortenberg, Germany). Emission spectra were measured in 2 nm steps between 425 nm and 601 nm for TS and between 570 nm and 650 nm for mCherry, after excitation at 400 ± 5 nm and 540 ± 10 nm, respectively. The excitation spectra were measured in 2 nm steps between 350 nm and 494 nm for TS and between 400 nm and 588 nm for mCherry at a fixed emission detection of 520 ± 5 nm and 615 ± 9 nm, respectively. For measurements over time, the NAPstar biosensors were excited at 400 ± 5 nm and 540 ± 10 nm with detection of the emission at 520 ± 5 nm and 615 ± 9 nm for TS and mCherry respectively. NAD(P)H autofluorescence was measured with excitation at 340 ± 7.5 nm and the emission was detected at 445 ± 10 nm.

The pH titration experiments were performed in 100 mM HEPES, 150 mM NaCl_2_, 0.5 mM MgCl_2_ and pH between 6 and 9 adjusted by KOH, with the actual pH measured post-hoc. For enzyme-coupled assays, 50 µU alkaline phosphatase FastAP (EF0651, Thermo Fisher Scientific, Waltham, Massachusetts, United States), 5–15 µU glutathione reductase (G3664-100UN, Sigma Aldrich, St. Louis, MO, United States)) and 1.07 ng/µL^−1^ isocitrate dehydrogenase (I2411-10UG, Sigma Aldrich, St. Louis, MO, United States) were used. The alkaline phosphatase assay was performed in 10 mM Tris-HCl, 5 mM MgCl_2_, 100 mM KCl, 0.02% (v/v) Triton-X-100 and 0.1 µg/µL BSA, pH 8.0. The glutathione reductase assay was performed in 50 mM Tris-HCl, 100 mM NaCl, 0.5 mM MgCl_2_, and 1 mM EDTA, pH 7.5. The isocitrate dehydrogenase assay was performed in 50 mM Tris-HCl, 100 mM NaCl, 5 mM MgCl_2_, 2.4 mM KCl and 0.65 µg/µL BSA, pH 8.0.

### Growth of yeast strains

The *S. cerevisiae* BY4742 (MATα *his3*Δ1 *leu2*Δ0 *lys2*Δ0 *ura3*Δ0) strain background^78^ was used for all fluorescence plate-reader experiments. For all fermentor experiments a CEN.PK113-1A yeast strain was used^34^. All yeast strains used in this study (Supplementary Table 7) were grown as previously described in Hartwell’s Complete (HC) medium supplemented with 2% (w/v) glucose as carbon source^79^ and lacking histidine for plasmid selection unless stated otherwise.

### Construction of yeast strains and transformation

Yeast gene deletions were generated by PCR-based homologous recombination^80^ (Supplementary Table 7). An antibiotic resistance cassette was PCR-amplified from either pFA6α *natNT2* or pFA6α *kanMX4* plasmids using primers designed to have 40–50 base-pairs of homology directly up- and down-stream of the gene to be deleted. Deletion of *ZWF1, TRX1* and *TRX2* in a BY4742 background was achieved using the primer pairs Pr7/Pr8, Pr9/Pr10, Pr11/Pr12 respectively. Deletion of *HIS3* in a CEN.PK113-1A background was achieved by using the primer pair Pr13/Pr14 (Supplementary Table 6).

The PCR products of these reactions were transformed into yeast cells using a standard lithium acetate-based approach. Briefly, cells were grown in YPD until logarithmic phase, harvested by centrifugation at 1000 *g* for 3 min at room temperature, and resuspended in 200 µl of ‘One-step-transformation’ buffer containing 40% polyethylene glycol 3350 (Sigma Aldrich, St. Louis, MO, United States), 0.2 M lithium acetate (Sigma Aldrich, St. Louis, MO, United States) and 0.1 M dithiothreitol (DTT; AppliChem GmbH, Darmstadt, Germany). Following the addition of 10 µl of salmon testes single-stranded DNA and the PCR product, the cells were incubated with continuous shaking for 30 min at 45 °C. Subsequently, the cells were transferred to fresh YPD and grown overnight before being plated onto YPD plates containing the appropriate antibiotic. After two rounds of selection, gene deletions were confirmed by PCR on genomic DNA using primers binding ~400 base pairs up- and down-stream of the gene of interest. DNA was extracted by heating yeast cells at 96 °C in 0.2% (w/v) SDS for 10 min. Subsequently cells were vortexed thoroughly and spun down at 11,000 *g* for 1 min. 1 µL of genomic DNA-containing supernatant was used as a template for the PCR reaction.

### Plate reader-based fluorescence measurements in *S. cerevisiae*

Yeast strains were either transformed with empty p413TEF plasmids for fluorescence background subtraction or p413TEF plasmids containing the indicated genetically encoded sensor. For plasmid transformation into yeast cells, a standard lithium acetate-based approach was used as described above. Yeast cells were harvested and resuspended in 100 µl of ‘One-step-transformation’ buffer before 5 µl of salmon testes single-stranded DNA and ~200 ng of plasmid DNA were added. The cells were then incubated with continuous shaking at 45 °C for 30 min, before being plated on HC medium supplemented with 2% (w/v) glucose as a carbon source and lacking histidine to ensure plasmid retention. The plates were then incubated for 2 days at 30 °C. The fluorescence of the NAPstar and Peredox constructs was measured using excitation at 399 ± 10 and 510 ± 10 nm with emission at 578 ± 15 and 619 ± 15 nm for the TS and mCherry domains respectively. For data calculation background fluorescence was subtracted before intensiometric TS fluorescence signals were divided by mCherry signals to allow for a ratiometric, probe expression-independent readout. For all roGFP2-Grx1 and NERNST measurements, roGFP2 was excited at either 400 ± 15 nm or 480 ± 15 nm with emission at 520 ± 20 nm. Calculation of the degree of oxidation (OxD roGFP2) was performed according to Eq. 1:

1 $$	{{{\rm{Ox}}}}{{{{\rm{D}}}}}_{{{{\rm{roGFP}}}}2}= \\ 	 \frac{(I400{{{\rm{sample}}}}*I480{{{\rm{red}}}})-(I400{{{\rm{red}}}}*I480{{{\rm{sample}}}})\,}{(I400{{{\rm{sample}}}}*I480{{{\rm{red}}}}-I400{{{\rm{sample}}}}*I480{{{\rm{ox}}}})+(I400{{{\rm{ox}}}}*I480{{{\rm{sample}}}}-I400{{{\rm{red}}}}*I480{{{\rm{sample}}}})\,}$$

For plate-reader experiments, yeast cultures were grown to late logarithmic phase (OD_600_ = 3–4) in liquid HC medium lacking histidine for plasmid retention. The cells were then harvested by centrifugation at 1000 *g* for 3 min at room temperature and resuspended in 100 mM MES/Tris buffer, pH 6, to an OD_600_ of 7.5. 200 µL aliquots were then transferred to the wells of a NUNC flat-bottomed 96-well microtiter plate (VWR International GMBH, Darmstadt, Germany). Subsequently, the plate was centrifuged at 30 *g* for 5 min at room temperature, so that cells form loose pellets at the bottom of each well. The measurement was initiated by addition of the indicated treatment and fluorescence changes of each probe were monitored for 60 min using a CLARIOstar (BMG Labtech, Ortenberg, Germany) fluorescence plate-reader. All experiments were performed at least three times with cells from independent cultures.

### Online monitoring of metabolite dynamics in continuous fermentor cultures

A Biostat A fermentor (Sartorius Stedim Systems GmbH, Guxhagen, Germany) was used to measure population-synchronised metabolic oscillations in continuous cultures with respect to the yeast metabolic cycle^34^. For all fermentor-based experiments a CEN.PK113-1A strain background was used. A CEN.PK113-1A ∆*his3* strain was generated to allow for retention of *HIS3*-containing p413TEF plasmids. This strain was transformed with p413TEF plasmids with the indicated genetically encoded probes. Culture media consisted of 10 g/L glucose, 1 g/L yeast extract, 5 g/L (NH_4_)_2_SO_4_, 2 g/L KH_2_PO_4_, 0.5 g/L MgSO_4_·7H_2_O, 0.1 g/L CaCl_2_·2H_2_O, 0.02 g/L FeSO_4_·7H_2_O, 0.01 g/L ZnSO_4_·7H_2_O, 0.005 g/L CuSO_4_·5H_2_O, 0.001 g/L MnCl_2_·4H_2_O, 2.5 mL of 70% H_2_SO_4_ and 0.5% (v/v) Antifoam 204 (Sigma Aldrich, St. Louis, MO, United States). Fermentor runs were initiated by the addition of a 20 ml starter culture grown at 30 °C to stationary phase in HC medium lacking histidine for plasmid retention. The fermentor was run with a working volume of 800 ml at 30 °C with constant aeration of 1 L.min^-1^ and stirring at 530 rpm. The automated addition of 10% (w/v) NaOH maintained a constant pH of 3.4. The culture was initially run in batch-culture mode until ~6 h after the exhaustion of the carbon source as determined by continuous and automated monitoring of the oxygen saturation within the culture vessel every 10 s. A continuous culture was subsequently initiated by addition of fresh medium at a dilution rate of 0.05 h^-1^. To enable the continuous ‘online’ monitoring of genetically-encoded probes, an in-house coupled fermentor-fluorimeter system was previously developed^34^. A peristaltic pump was used to continuously pump culture from the fermentor through a flow cell (Type 71-F, Starna GmbH, Pfungstadt, Germany), which was inserted into a JASCO FP-6500 spectrofluorimeter (JASCO, Oklahoma City, OK, United States) before media returned to the fermentor vessel. The fluorescence of the PeredoxDS and NAPstar probes was measured at fixed excitation and emission wavelengths of 399 ± 10 and 510 ± 10 nm or 578 ± 10 and 619 ± 10 nm for the TS and mCherry domains respectively. The fluorescence of HyPer7 was measured at fixed excitation wavelengths of 400 ± 10 nm and 490 ± 10 nm with emission monitored at 520 ± 10 nm. A slit width of 10 nm was used, and fluorescence measurements were continuously performed and recorded every 10 s.

### Generation and cultivation of Arabidopsis plants

Transformation of *Arabidopsis thaliana* Col-0 plants for stable expression of NAPstar constructs was performed by floral dip^81^. Transformants were selected based on hygromycin resistance and sensor protein fluorescence. We isolated between 5–12 independent lines for each of the constructs. At least three lines with bright fluorescence were taken forward to homozygosity. The sensor fluorescence in the leaf epidermis was validated for all selected lines to show the characteristic cyto-nuclear pattern, a high signal-to-noise ratio when compared to the wild-type control, and clear separation of the biosensor fluorescence from chlorophyll autofluorescence. Other plant biosensor lines were used as reported previously; Peredox^23^, Grx1-roGFP2^63^, roGFP1-Orp1^82^ and NERNST^18^. Plants were cultivated on soil made up of 50% (v/v) VMV800 Vermehrungserde and 50% (v/v) EDE800 Einheitserde (Balster Einheitserden, Frödenberg, Germany) under long-day conditions (16 h light, 8 h dark; light period: 100 to 150 µmol photons m^-2^ s^-1^ by OSRAM HO 54 W/840 LUMILUX Cool White tubes; 22 °C during light and 18 °C during darkness; 65% humidity). After sowing, the seeds were stratified at 4 °C for 2–3 days before the pots were moved into the cultivation chambers. Pots were watered from the bottom every 3–4 days.

### *In planta* biosensor measurements

The multiparametric real-time measurements were conducted with a CLARIOstar multiwell plate-reader equipped with an atmospheric control unit (BMG Labtech, Ortenberg, Germany). Leaf disc samples were cut from approximately five-week-old Arabidopsis rosettes with a leaf disc cork borer as described previously^23^. The leaf discs were transferred to the wells of a NUNC 96-well plate (VWR International GMBH, Darmstadt, Germany) each filled with 200 µL of 10 mM MES, 10 mM MgCl_2_, 10 mM CaCl_2_, and 5 mM KCl, pH 5.8. The experiments were performed at 25 °C in the early afternoon. The dark-light transition experiments were carried out as described previously^23^. The plate was removed from the plate reader for the duration of the illumination and placed back immediately after. An intensity of 600 µE was used for white light illumination with ELRO LED 1 × 7 W IP44 (ELRO Europe, Amsterdam, Netherlands).

The hypoxia assays were performed as described previously^40,41,76,83^ with several adjustments. The sensor fluorescence response was measured over a time course and an oxygen ramping script mode together with the ACU was used to apply an oxygen gradient by flushing the measurement chamber with nitrogen gas (99.998 % v/v, Westfalen AG, Münster, Germany). The script mode was used to set a standardised measurement regime of 2.5 h under normoxic conditions, followed by 0.5 h of oxygen depletion to a defined hypoxic O_2_ concentration and a hypoxic phase of 6 h, followed by reoxygenation with ambient air within 0.5 h. The fluorescence was then recorded for another 7.5 h. The fluorescence readout of the sensors was recorded using the following excitation and emission bands: Peredox, NAPstar4.3 and NAPstarC: TS: Ex = 400 ± 5 nm, Em = 520 ± 5 nm; mCherry: Ex = 540 ± 10 nm, Em = 615 ± 9 nm; Grx1-roGFP2/roGFP2-Orp1, Ex = 400 ± 5 nm and 482 ± 8 nm, Em = 520 ± 5 nm). The detector gain was set to allow an optimal detection range while avoiding detector overflow and was kept constant for all measurements within an experiment. The emission was recorded using the top optic detector and the orbital averaging mode (diameter: 3 mm and 30-35 light flashes).

Background and autofluorescence were corrected by subtracting the average emission from wild-type Col-0 tissue measured in parallel using the same treatments but without sensor expression. Biosensor fluorescence ratios were calculated for Peredox and NAPstar family as TS/mCherry and for the roGFP2 family as 400/482 nm. The data were then log10-transformed. The data were normalized to zero by subtracting an average of the last five intensity values before oxygen depletion or before treatment from each time point value.

### Fluorescence lifetime imaging microscopy (FLIM)

For in vitro lifetime measurements, 0.025 µg/µL NAPstar4.3 diluted in binding buffer, containing 150 µM NADP^+^ was added to imaging 8-well plates (80806; ibidi, Gräfelfing, Germany). Increasing amounts of NADPH (1–250 μM) were subsequently added to the wells and the solutions were imaged on an LSM880 confocal laser scanning microscope (Carl Zeiss AG, Oberkochen, Germany) with a 63x immersion objective (LD LCI Plan-Apochromat 63x/1.2 Imm Corr DIC M27) using a 440 nm pulsed excitation laser (LDH-D-C-440, 40 MHz repetition rate) managed by a PicoQuant FLIM module (Sepia PDL828-S, PMA Hybrid 40, MultiHarp 150 4N, Time Harp 260 PICO Dual) (PicoQuant, Berlin, Germany). Fluorescence lifetime images of 512 × 512 pixels (135.2 μm x 135.2 μm) and 2 cycles or 5 × 10^5^ photon counts at a pixel dwell time of 16.38 μs passing through a 550 ± 49 nm bandpass filter (Semrock, F37-551, IDEX Health & Science, LLC, Rochester, NY, United States) were recorded using the handshake plugin between Zen Black imaging software (Carl Zeiss AG, Oberkochen, Germany) and SymPhoTime 64 software (PicoQuant, Berlin, Germany). Ten FLIM images were recorded per treatment and measurements were repeated over three experimental days. Each set of images was analysed using the ‘Grouped FLIM’ analysis in SymPhoTime 64 with a bi-exponential fitting model. The obtained amplitude weighted averaged lifetime, τ_Av Amp_, values were averaged for the set and used to plot the K_d_ curves.

### Cell culture and transient transfection

HeLa cells (Leibnitz Institute DSMZ; ACC 57) at passage 4–25 were cultured in Dulbecco’s Modified Eagle Medium (DMEM) (Gibco, Ref: 41966029, Thermofisher Scientific,Waltham, Massachusetts, United States) supplemented with 10% foetal bovine serum (FBS) (Gibco, Ref: 10270106, Thermo Fisher Scientific, Waltham, Massachusetts, United States) in humidified air at 37 °C and 5% CO_2_. The cells were subcultured twice a week at a ratio of 1:5 to 1:8.Transient transfection of HeLa cells (1–2 × 10^5^ cells/6-well) with pcDNA3.1(+) NAPstar or pSC2 HyPer7 (Addgene; plasmid #136466, Watertown, MA, United States) plasmids was performed with the transfection reagent FuGENE® HD (Promega, Ref: E2311, Promega GmbH, Walldorf, Germany) at a 2:1 FuGENE® HD:DNA ratio according to the manufacturer’s protocol. After 48 h of incubation under regular culture conditions the cells were used for fluorescence microscopy measurements.

### Fluorescence microscopy

#### Epifluorescence microscopy

NAPstar or HyPer7 expressing HeLa cells were analysed with a Zeiss Axio Observer 7 inverted epifluorescence microscope and the ZEN3.2 blue edition software (Carl Zeiss AG, Oberkochen, Germany). NAPstar expression levels in HeLa and yeast cells were imaged using the following excitation and emission settings: Ex: 405 ± 20 nm, Em: 525 ± 50 nm and Ex: 572 ± 25 nm, Em: 629 ± 62 nm. Yeast cells were imaged using a 100x objective, HeLa cells were imaged using a 63x objective. Cells were imaged in glucose-free HBSS. Images were analysed using ImageJ software.

For perfusion assays with HeLa cells, NAPstar3b was monitored using the Ex and Em settings as above, HyPer7 was monitored using- Ex: 405 ± 20 nm, Em: 525 ± 50 nm and Ex: 470 ± 40 nm, Em: 525 ± 50 nm; Cells were preincubated in glucose-free or 10 mM glucose containing Hank’s Balanced Salt Solution (HBSS) (1.3 mM CaCl_2_, 0.8 mM MgSO_4_, 5.4 mM KCl, 0.44 mM KH_2_PO_4_, 4.2 mM NaHCO_3_, 137 mM NaCl, 0.34 mM Na_2_HPO_4_ pH 7.4) for 1 h at 37 °C and 5% (v/v) CO_2_. The measurement was performed in HBSS containing 0 mM or 10 mM glucose supplemented with H_2_O_2_ (2.5–20 µM) or menadione (1–10 µM) and fresh buffer solution were continuously provided by a peristaltic pump adjusted to a flow rate of 1 mL/min.

#### Confocal microscopy

A Zeiss LSM980 confocal microscope operated using the ZEN3.6 blue edition software (Carl Zeiss AG, Oberkochen, Germany) was used for plant cell imaging. A 40x C-Apochromat lens with 1.2 numeric aperture and water immersion was used. The pinhole was set in range of 1–2 airy units. Leaf disks used for microscopy were incubated in the dark for at least 2 h before imaging. The TS fluorophore and chlorophyll autofluorescence were excited at 405 nm and emitting light was measured between 497 nm and 542 nm (TS) and in between 654 nm and 701 nm (chlorophyll). The mCherry fluorophore was excited at 561 nm and the emission was measured between 588 nm and 632 nm.

### Generation of CRISPR KO strains

To generate a knockout of glutathione reductase (GSR) in HEK293 cells, a guide RNA sequence targeting exon 1 was cloned into the pSpCas9(BB)-2A-GFP (PX458) vector, gifted from Feng Zhang (Addgene plasmid #48138, Watertown, MA, United States)^84^. HEK flp-InTm T-RExTM-293 cells (Invitrogen, Darmstadt, Germany) were transfected with this construct using a standard polyethylenimine-based approach. 24 h after transfection, cells were sorted by FACS, based on GFP fluorescence. Cells were then seeded on a single-cell basis into 96-well plates and screened for loss of protein using western blot analysis (Proteintech, Cat. Number: 18257-1-AP, GSR Rabbit polyclonal antibody).

### Cytation measurements

High-throughput imaging measurements of HEK293 cells expressing NAPStar3b from a pcDNA3.1(+) plasmid and data analysis were performed as previously described^85^. Experiments were performed using wildtype and GSR KO HEK293 cells. In each well of a poly-L-lysine coated 96-well plate (μclear, GreinerBio, Kremsmünster, Austria), approximately 4000 cells were seeded in 100 µl Dulbecco’s Modified Eagle Medium (DMEM) containing 10% foetal calf serum (FCS) and 1% penicillin and streptomycin (P/S). 24 h after seeding, cells were transfected with the plasmid containing the indicated construct using a standard polyethylenimine-based method.

After 48 h, the measurement was started by switching from DMEM medium to preheated glucose starvation minimal medium (140 mM NaCl, 5 mM KCl, 1 mM of MgCl_2_, 2 mM of CaCl_2_, 20 mM HEPES, pH 7.4 adjusted with NaOH) with 10% FCS. Subsequently, the plate was incubated in the Cytation3 (BioTek, Winooski, VT, United States) at 37 °C with 5% (v/v) CO_2_ for 30 min. The sensor fluorescence was measured using the 10x air objective. A 405 nm and 590 nm BioTek filter cube was used for readouts of either 400 ± 40 nm or 586 ± 15 nm respectively. After the incubation step, the steady state was monitored for 30 min. For inhibition of thioredoxin reductase or glutathione reductase activity, cells were subsequently treated with 1 µM auranofin (Sigma Aldrich, Darmstadt, Germany) or 50 µM 1,3-bis(2-chloroethyl)-1-nitrosourea (BCNU) (Sigma Aldrich, Darmstadt, Germany) respectively for 1 h. Following the treated with 50 and 100 µM H_2_O_2_ or 100 and 500 µM diamide, sensor responses were monitored for 1 h. Data analysis was performed using the RRA custom Matlab analysis suite^86^ and 490 nm over 585 nm ratios were calculated.

### Statistics & reproducibility

The statistical analyses of the data were conducted using GraphPad Prism and MS Excel using the tests indicated in the legends of the individual figure panels. The presented experiments were initially optimised and then reproduced with similar results. Plate reader data were manually checked before analysis for technically invalid replicates, e.g. due to detector saturation, which were excluded. The experiments were not randomised. The investigators were not blinded.

### Reporting summary

Further information on research design is available in the Nature Portfolio Reporting Summary linked to this article.

## Supplementary information

Supplementary Information File

Reporting Summary

Transparent Peer Review file

## Source data

Source Data

## Data Availability

All data supporting the results presented in the Manuscript and the Supplementary Figs. is provided as Source Data. The original raw files from the measurements are documented by the contributing labs and are available upon request. The following Arabidopsis accessions were used as backgrounds as available from the European Arabidopsis Stock Center (NASC): *gr1* SALK_105794^63^; *ntr a x ntrb* SALK_539152 and SALK_545978^87^ referred to as *ntr* *a/b* in this manuscript. Primers and plasmid lists are provided in the Supplementary Information. Source data are provided with this paper.
