# Supplementary material for: A family of NADPH/NADP+ biosensors reveals in vivo dynamics of central redox metabolism across eukaryotes
Source: Nat Commun. 2024 Dec 19;15:10704. doi: 10.1038/s41467-024-55302-x (PMC11659435; doi:10.1038/s41467-024-55302-x)
Supplement: Supplementary file 1 — Supplementary Information File [file 41467_2024_55302_MOESM1_ESM.pdf]

Supplementary Information

A family of NADPH-NADP$^+$ biosensors reveals *in vivo* dynamics of central redox metabolism across eukaryotes

Marie Scherschel[1*], Jan-Ole Niemeier[2*], Lianne J.H.C. Jacobs[3], Markus D.A. Hoffmann[4], Anika Diederich[1], Christopher Bell[2], Pascal Höhne[1,2], Sonja Raetz[2], Johanna B. Kroll[2], Janina Steinbeck[2], Sophie Lichtenauer[2], Jan Multhoff[2], Jannik Zimmermann[1], Tanmay Sadhanasatish[5], R. Alexander Rothemann[3], Carsten Grashoff[5], Joris Messens[6,7,8], Emmanuel Ampofo[9], Matthias W. Laschke[9], Jan Riemer[3,10], Leticia Prates Roma[4], Markus Schwarzländer[2†], Bruce Morgan[1†]

Affiliations:

[1] Institute of Biochemistry, Center for Human and Molecular Biology (ZHMB), Saarland University, 66123 Saarbrücken, Germany

[2] Institute of Plant Biology and Biotechnology, University of Münster, Schlossplatz 8, 48143 Münster, Germany

[3] Redox Metabolism, Institute for Biochemistry, University of Cologne, 50674 Cologne, Germany

[4] Department of Biophysics, Center for Human and Molecular Biology (ZHMB), Saarland University, 66421 Homburg, Germany.

[5] Institute of Integrative Cell Biology and Physiology, University of Münster, Schlossplatz 5, 43149 Münster, Germany

[6] VIB-VUB Center for Structural Biology, Vlaams Instituut voor Biotechnologie, B-1050 Brussels, Belgium

[7] Brussels Center for Redox Biology, Vrije Universiteit Brussel, B-1050 Brussels, Belgium

[8] Structural Biology Brussels, Vrije Universiteit Brussel, B-1050 Brussels, Belgium

[9] Institute for Clinical & Experimental Surgery, Saarland University, 66421 Homburg, Germany

[10] Cologne Excellence Cluster on Cellular Stress Responses in Aging-associated Diseases (CECAD), University of Cologne, 50931 Cologne, Germany

[*] These authors contributed equally to this manuscript

[†] To whom correspondence should be sent:

Markus Schwarzländer: markus.schwarzlander@uni-muenster.de

Bruce Morgan: bruce.morgan@uni-saarland.de

Supplementary Figures

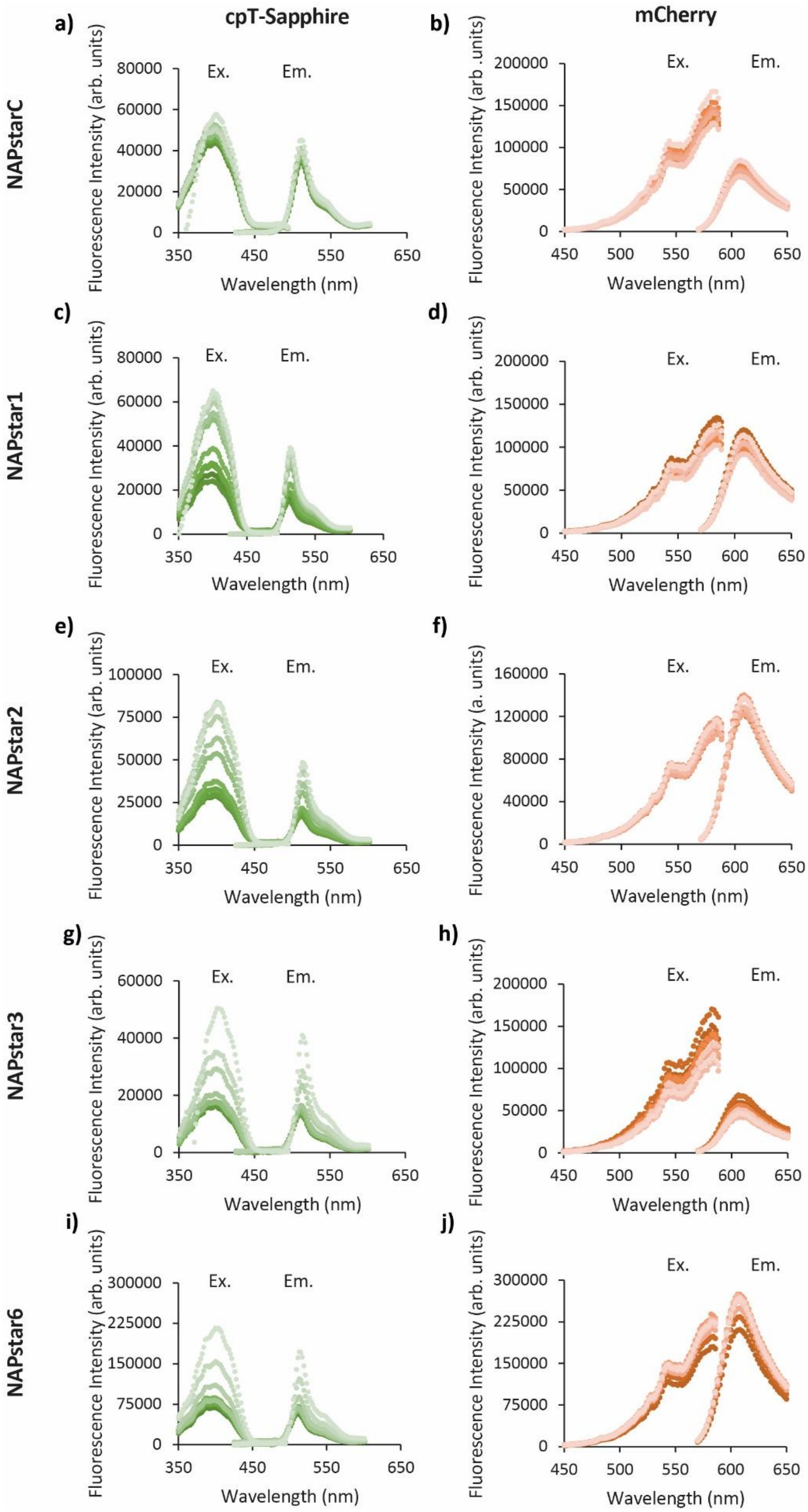

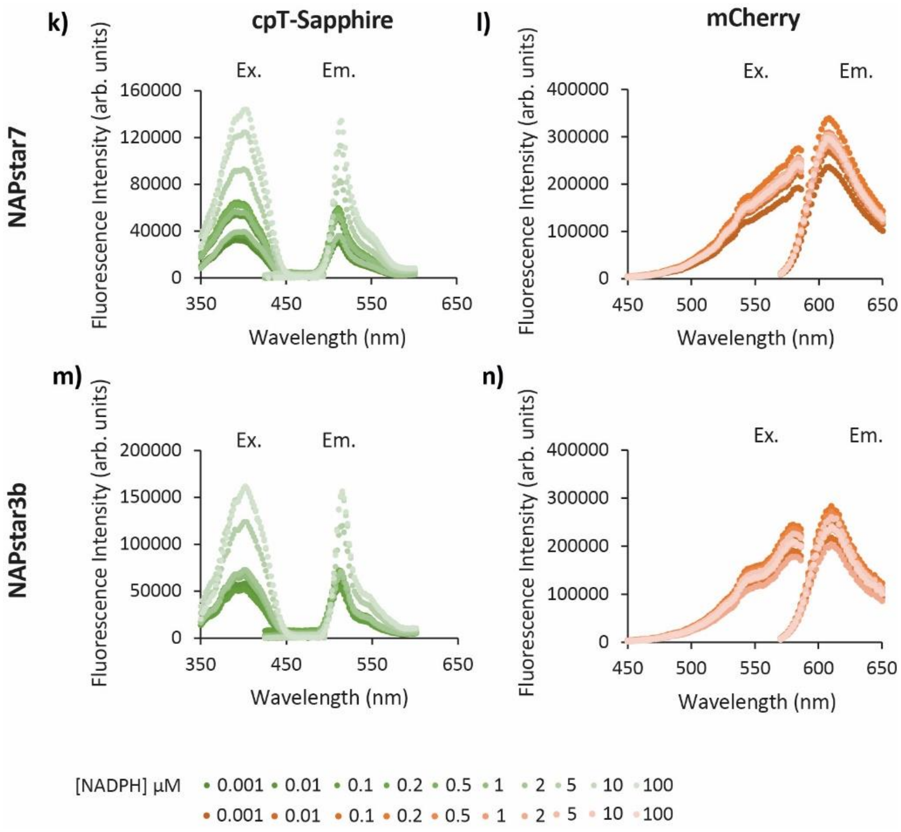

Supplementary Figure 1. Fluorescence spectra of NAPstars.

Fluorescence excitation (Ex.) and emission (Em.) intensity spectra of (**a,c,e,g,i,k,m**) the cpT-Sapphire and the mCherry (**b,d,f,h,j,l,n**) fluorophores of the different NAPstar variants, incubated in the presence of 150 µM NADP$^+$ and NADPH at the indicated concentrations ranging from 0–100 µM. Sensor protein concentrations were adjusted to 240 nM (n=3 technical replicates). Data are presented as means.

Supplementary Figure 2

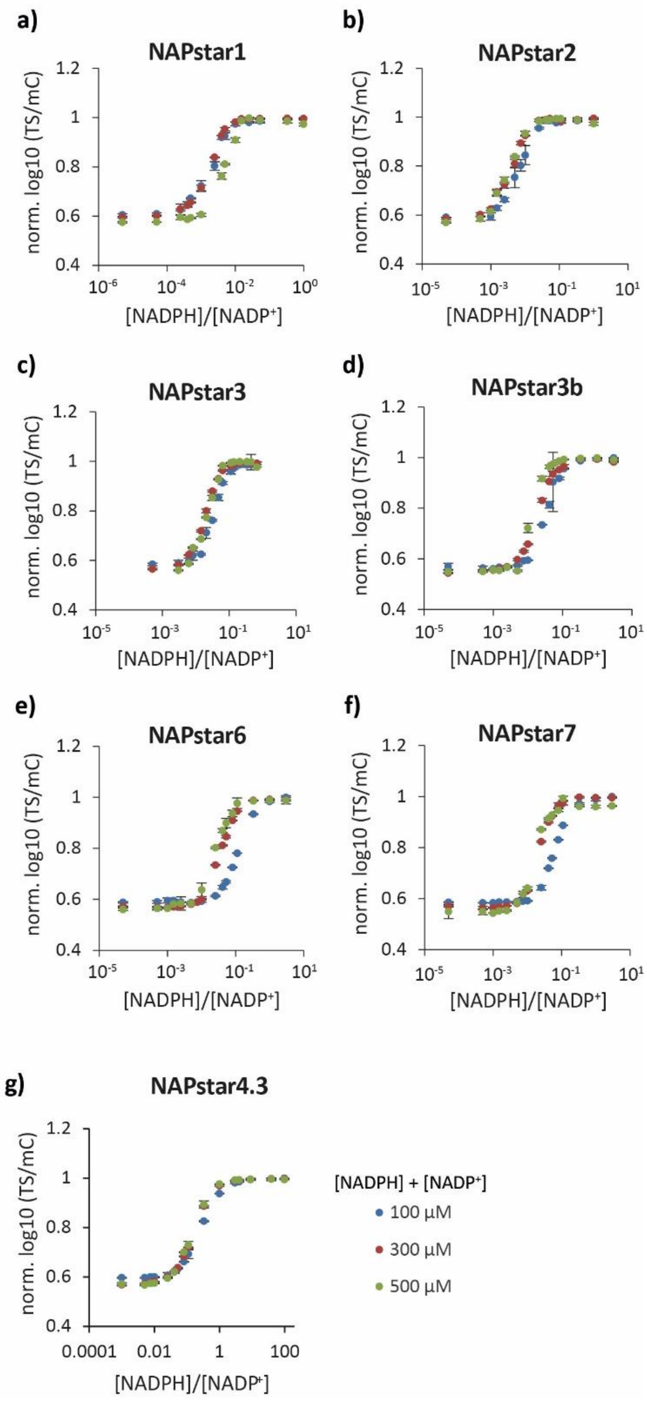

Supplementary Figure 2. NAPstars report the NADP redox state.

a–g, NAPstar variants were incubated in the presence of total NADP concentrations ([NADPH] + [NADP$^+$]) of 100 µM, 300 µM and 500 µM. Different NADP redox states were set by adjustment of the relative NADPH and NADP$^+$ concentrations. Sensor protein concentrations were adjusted to 240 nM for all measurements (n=3 technical replicates). Data are presented as mean ± s.d. normalised to the highest data point.

Supplementary Figure 3

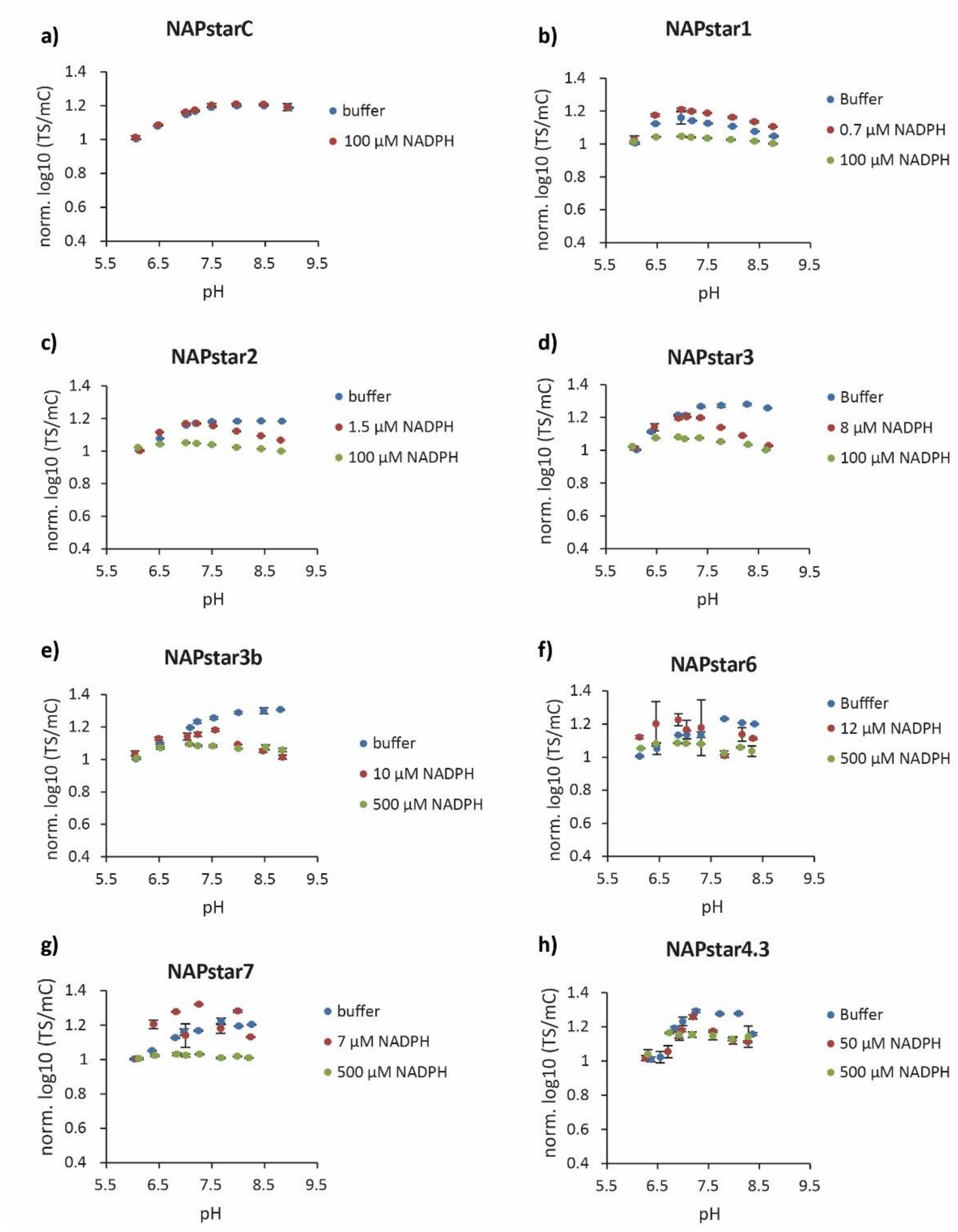

Supplementary Figure 3. NAPstar measurements are resistant to pH changes.

a–h, Individual NAPstar family proteins were incubated in buffers with different pH values spanning the physiologically relevant pH range (see Methods section). All probes were incubated in the presence of 150 µM NADP$^+$ and NADPH at the indicated concentrations to establish different NADPH or NADP$^+$ occupancies. NADPH concentrations were used to establish saturation by NADP$^+$ binding only (buffer; i.e. no NADPH; blue), approximately half occupancy by NADP$^+$ and NADPH each as informed by the the specific K_r of the respective NAPstar variant (red), and saturation by NADPH binding only (100 or 500 µM NADPH; green). An exception is NAPstarC (**a**) due to lack of substrate binding. Sensor protein concentrations were adjusted to 240 nM (n=3 technical replicates for all measurements). Data are presented as mean ± s.d. normalised to the lowest data point.

Supplementary Figure 4

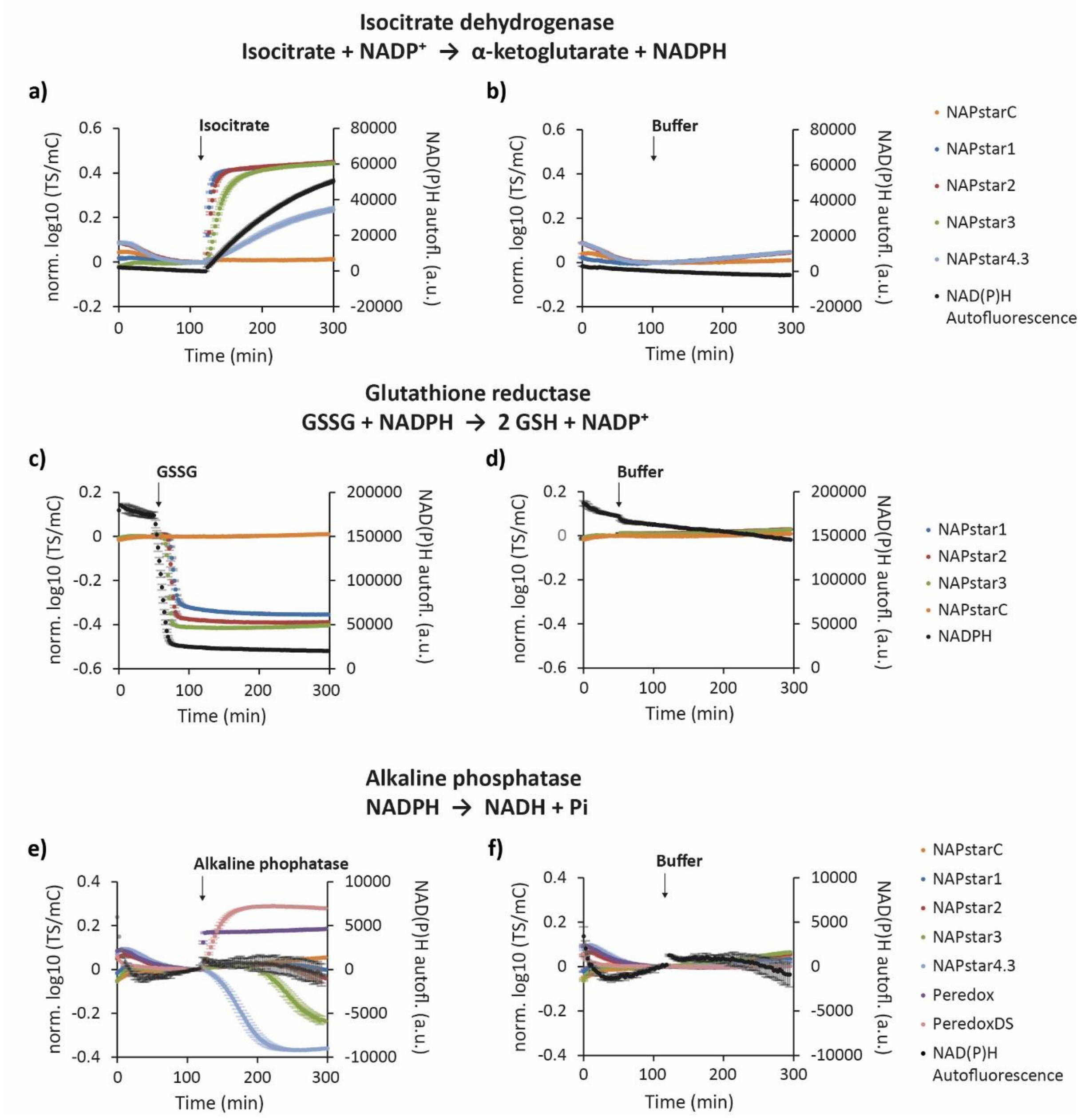

Supplementary Figure 4. NAPstar-based online monitoring of NADP redox dynamics in vitro.

a,b $NADP^+$ (initial concentration 500 µM) to NADPH conversion was initiated by the addition of 1 mM isocitrate in the presence of isocitrate dehydrogenase. **b,** buffer only addition as a control. **c,d** NADPH (initial concentration 100 µM) to $NADP^+$ conversion was initiated by the addition of 1 mM glutathione disulfide in the presence of glutathione reductase. **d,** buffer only addition as a control **e,f** NADPH (initial concentration 100 µM) to NADH conversion was initiated by the addition of alkaline phosphatase. **f,** buffer only addition as control Enzyme origin, quantities and assay buffer conditions are specified in the Methods section. Sensor protein concentrations were adjusted to 430 nM (n=3 technical replicates). Data are presented as mean ± s.d. normalised to the average before the treatment.

Supplementary Figure 5

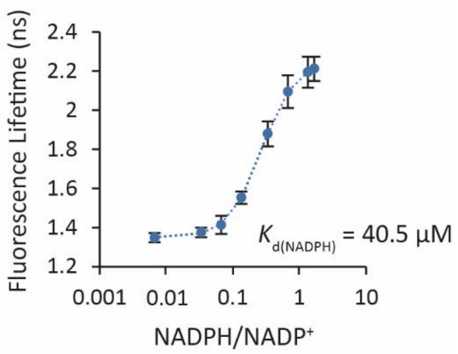

Supplementary Figure 5. NAPstars are well suited to fluorescence-lifetime measurement.

a, NAPstar4.3 was incubated in the presence of 150 µM $NADP^+$ and different NADPH/$NADP^+$ ratios were established by adjusting NADPH (1–250 µM). Fluorescence lifetime measurements of cpT-Sapphire signal were performed at 440 nm pulsed laser excitation and 550/49 nm bandpass filter emission as detailed in the Methods section. Sensor protein concentrations were adjusted to 240 nM. $K_{d(NADPH)}$ was calculated at 40.5 µM. (n=3 independent experiments, each with 10 technical replicates). Data are presented as mean ± s.d.

Supplementary Figure 6

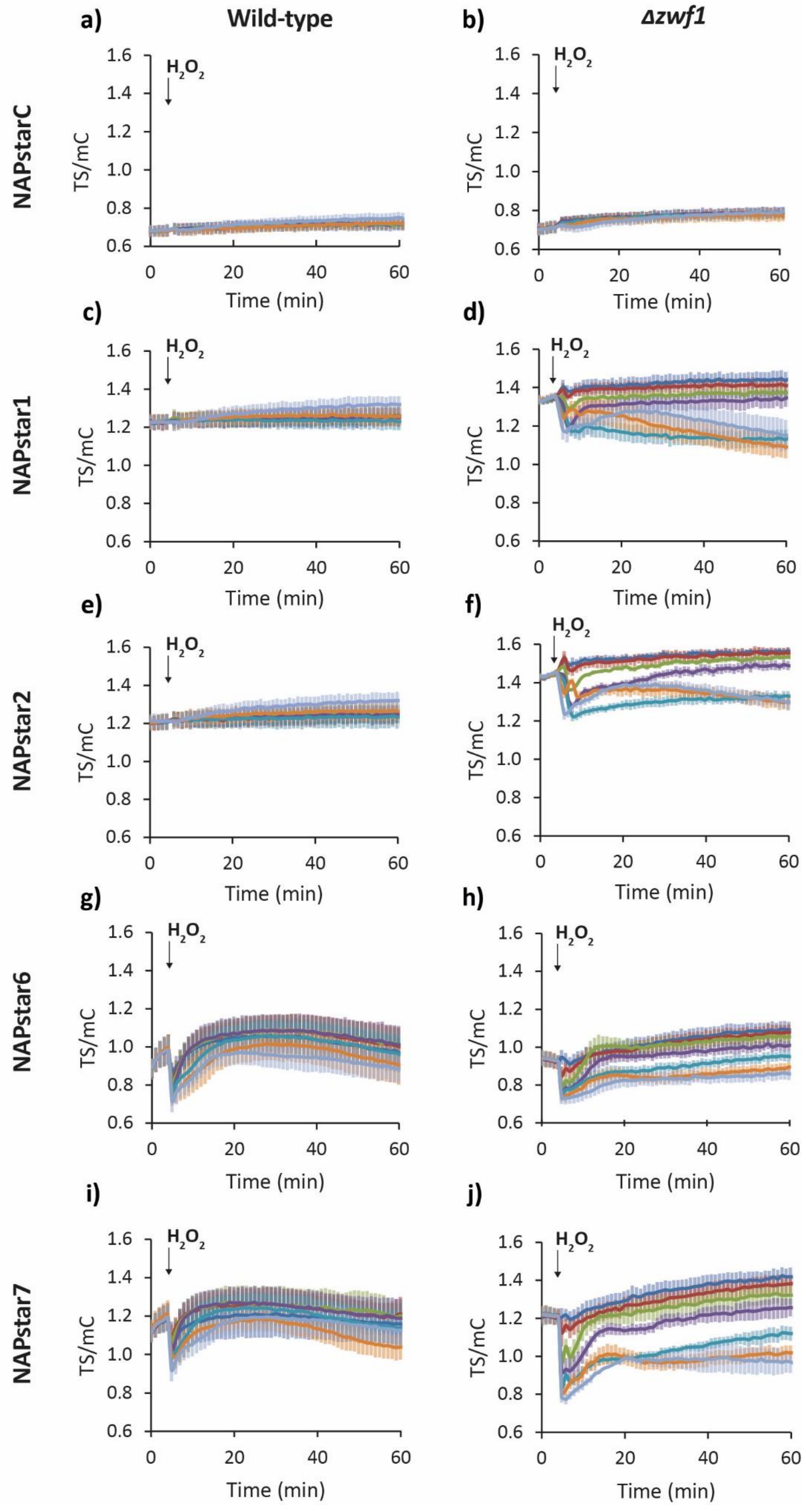

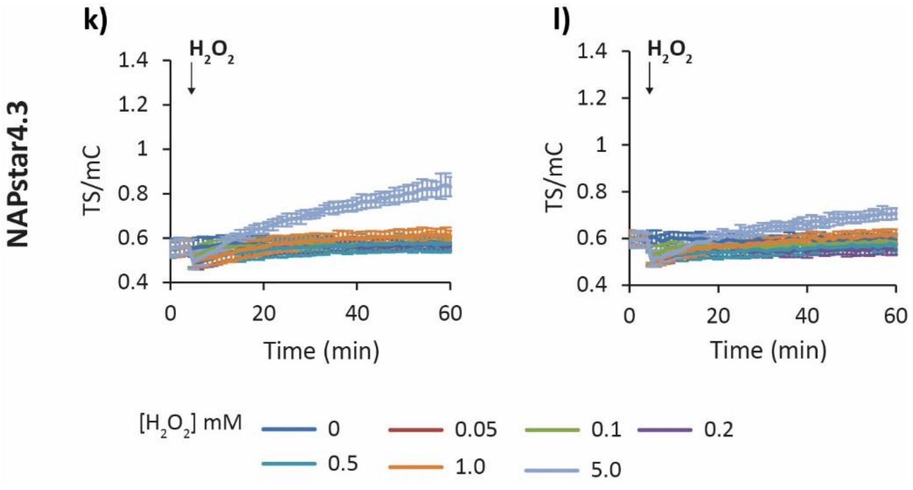

Supplementary Figure 6. NAPstar responses in the yeast cytosol to external H_2O_2.

Related to **Figure 2**. Response of wild-type (**a, c, e, g, i, k**) and *Δzwf1* (**b, d, f, h, j, l**) yeast cells expressing the indicated NAPstar variants, to treatment with H_2O_2 at the indicated concentrations (n=3 repeats with cells from independent cultures). Data are presented mean ± s.d. Identical experimental settings were used for all panels allowing for direct comparison of TS/mC between datasets.

Supplementary Figure 7

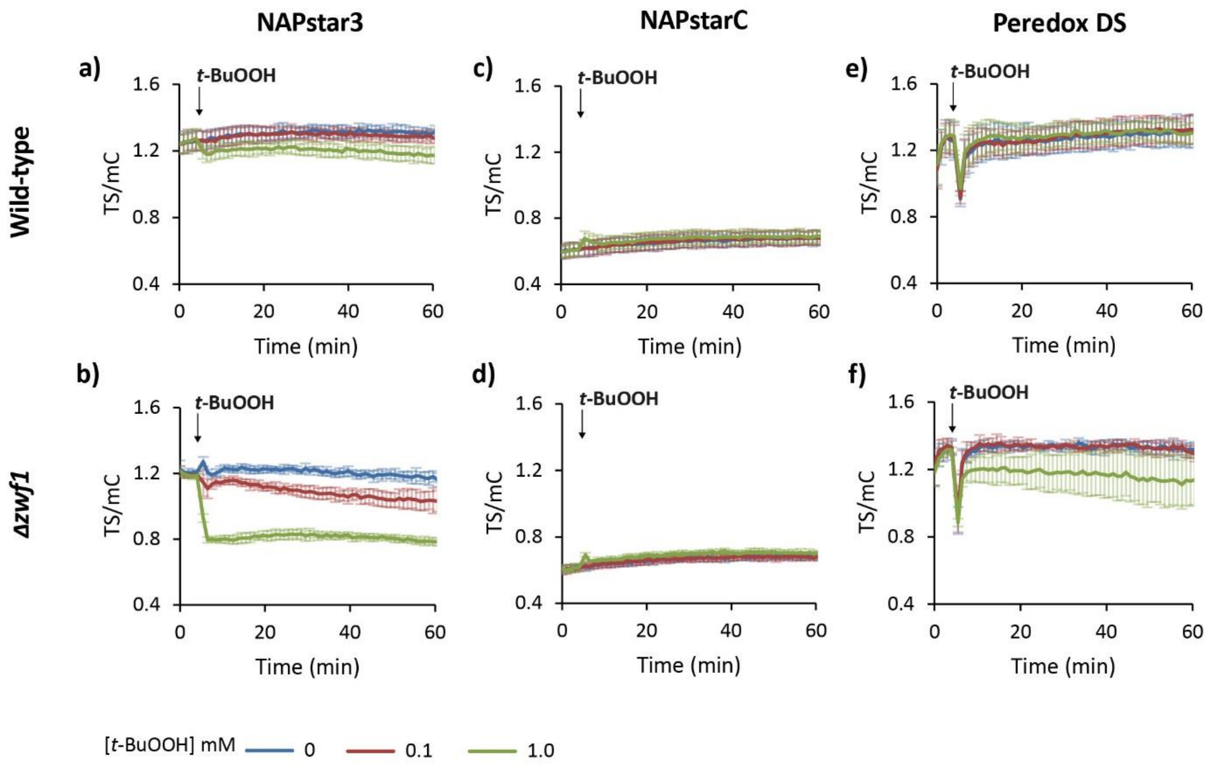

Supplementary Figure 7. Oxidative pentose pathway-derived NADPH robustly protects the cytosolic NADP pool against oxidation by *t*-BuOOH.

Related to **Fig. 2b–g**. **a–f,** Response of PeredoxDS, NAPstar3 and NAPstarC probes, expressed in the cytosol of wild-type and Δ*zwf1* yeast cells, to the addition of exogenous *t*-BuOOH at the indicated concentrations (n=3 experimental repeats in which *t*-BuOOH responses was monitored in cells derived from independent cultures). Data are presented mean ± s.d. Identical experimental settings were used for all panels allowing for direct comparison of TS/mC between datasets.

Supplementary Figure 8

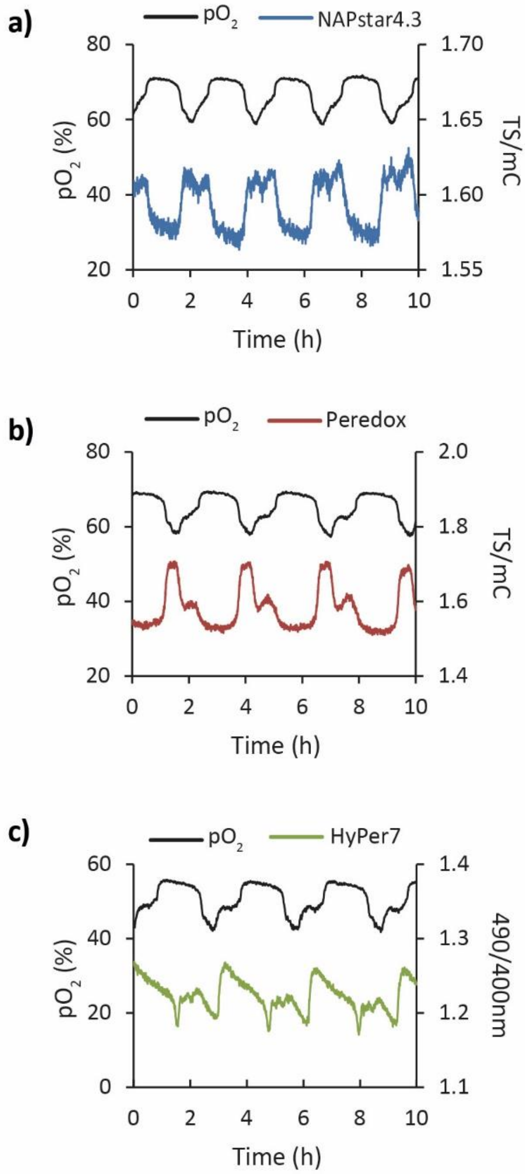

Supplementary Figure 8. Oscillations in cytosolic NADP and NAD redox state accompany the yeast metabolic cycle (YMC).

Related to **Figure 3**. **a–c,** Replicate traces supporting **Fig. 3c**, showing the changes in dissolved oxygen, NAPstar4.3 (NADP redox state), Peredox (NAD redox state) and Hyper7 (H_2O_2) during 3.5 complete cycles of the YMC.

Supplementary Figure 9

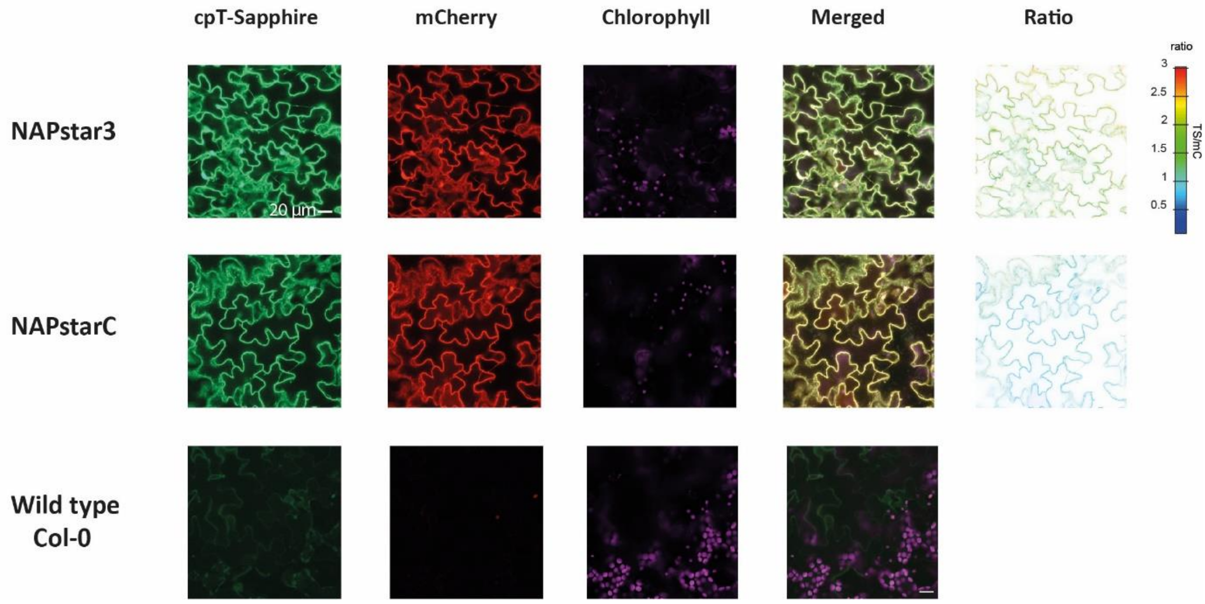

Supplementary Figure 9. NAPstar expression in Arabidopsis leaf epidermis.

Confocal microscopy images of NAPstar3, NAPstarC and the wild-type (Col-0) in the cytosol of *Arabidopsis thaliana* plants. Images show the abaxial leaf epidermal layer. Identical microscope settings were used for all lines (see Methods section). Corresponding ratio images that display TS/mC are shown on the right as calculated using the RRA custom software package (see Methods section). Scale bar = 20 µm.

Supplementary Figure 10

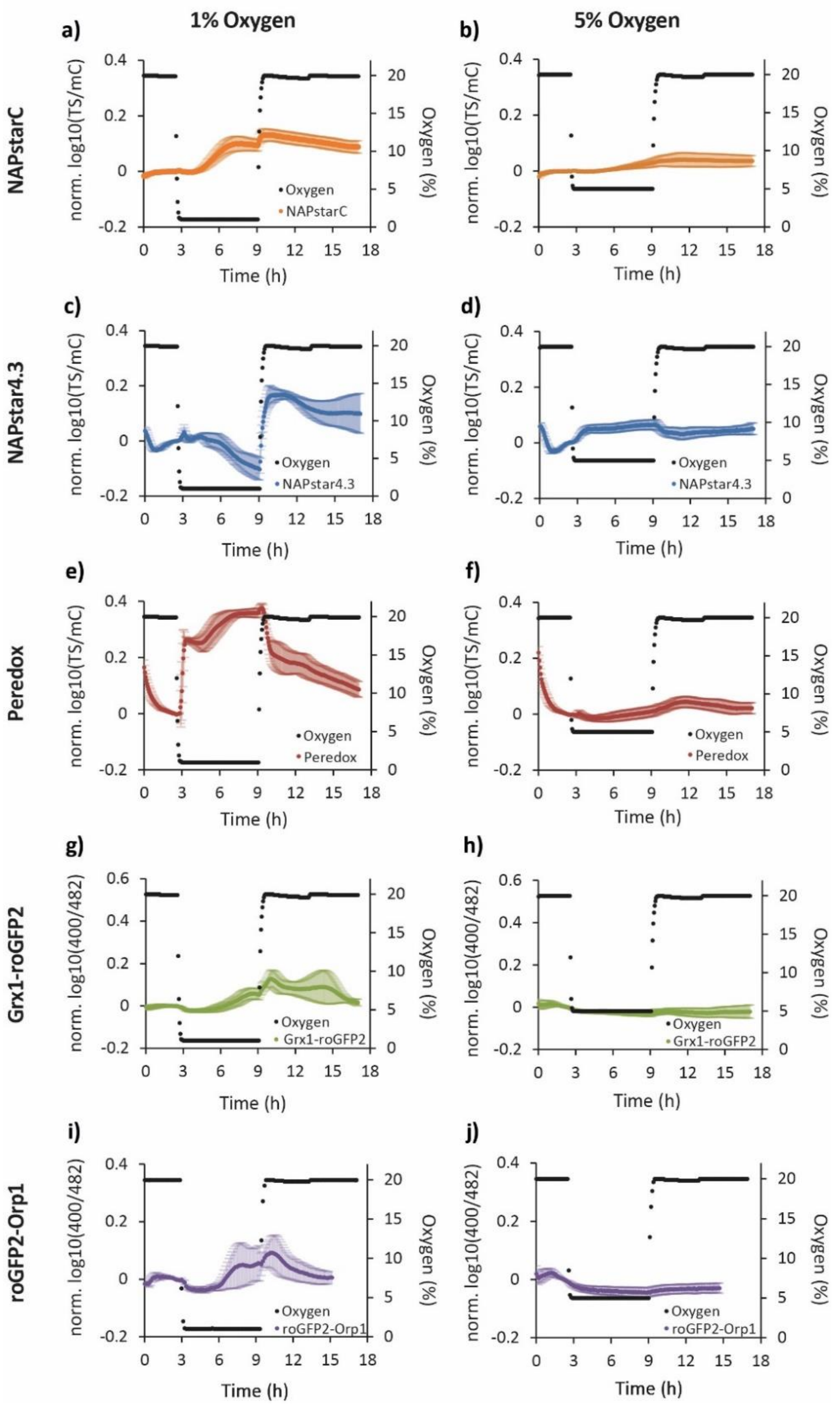

Supplementary Figure 10. Cytosolic redox dynamics accompany hypoxia-reoxygenation in plants.

Related to **Figure 4**. **a,b,** NAPstarC (control), **c,d,** NAPstar4.3 (NADP redox state), **e,f,** Peredox (NAD redox state), **g,h,** Grx1-roGFP2 (glutathione redox potential; E_{GSH}), and **i,j,** roGFP2-Orp1 (H_2O_2) dynamics in response to the indicated period of hypoxia with 1% and 5% oxygen respectively (n=7 for NAPstarC, NAPstar4.3 and Grx1-roGFP2, n=8 for Peredox at 1% oxygen; n=6 for NAPstarC, n=7 for Grx1-roGFP2, roGFP2-Orp1 and NAPstar4.3, and n=8 Peredox with 5% oxygen referring to individual leaf discs from individual plants). In all panels, data are presented as mean ± s.d. normalised to the average value before the induction of hypoxia.

Supplementary Figure 11

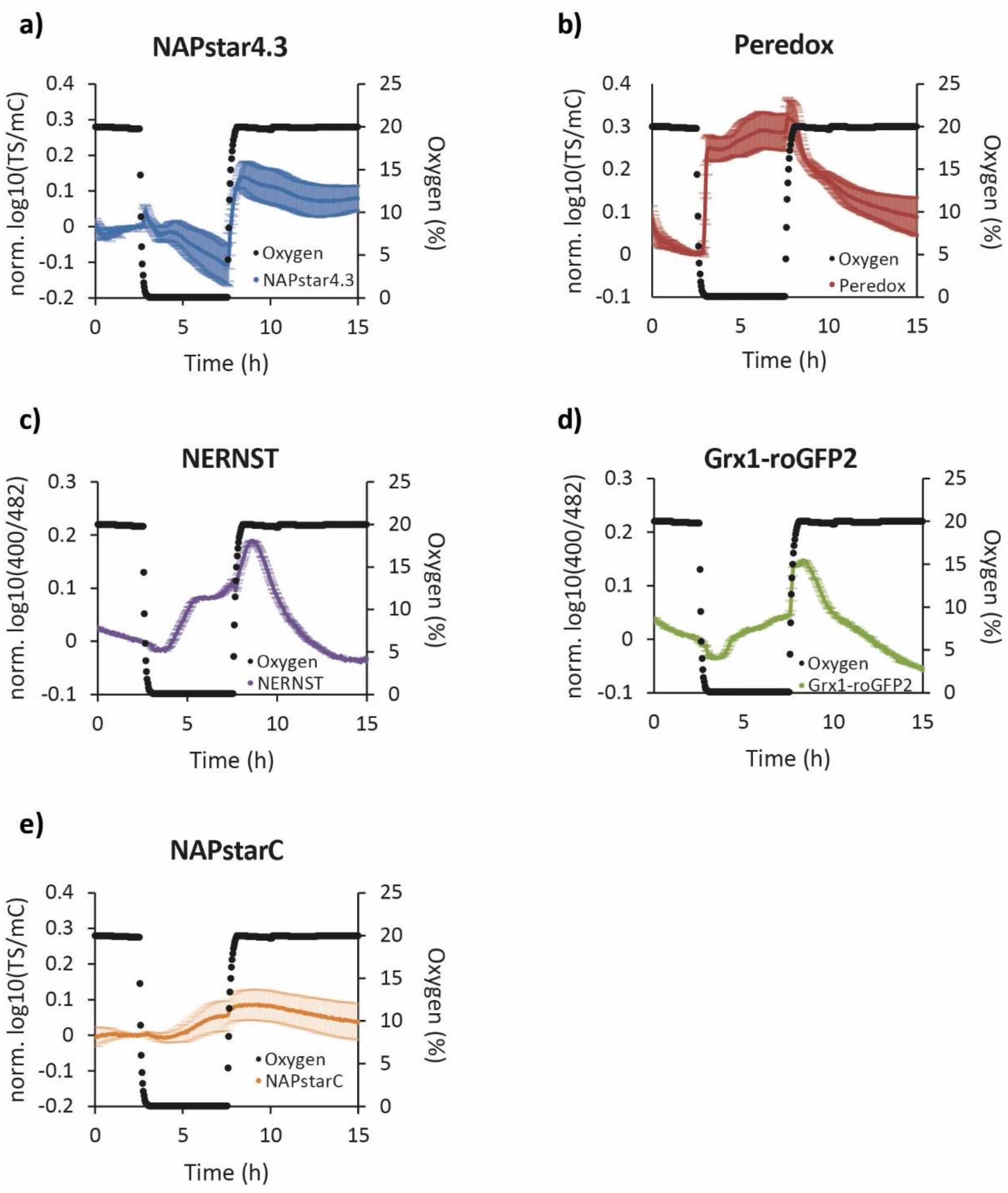

Supplementary Figure 11. NERNST resembles the dynamics of Grx1-roGFP2 but not NAPstar4.3 during hypoxia-reoxygenation in plants.

Related to **Figure 4**. **a–e,** Response of NAPstar4.3, Peredox, NERNST; Grx1-roGFP2 and NAPstarC probes to hypoxia with 0.1% oxygen (n=4 for Grx1-roGFP2 and n=6 for NERNST, NAPstarC, NAPstar4.3 and Peredox referring to individual leaf discs from individual plants). In all panels, data are presented as mean ± s.d. normalised to the average value before the induction of hypoxia.

Supplementary Figure 12

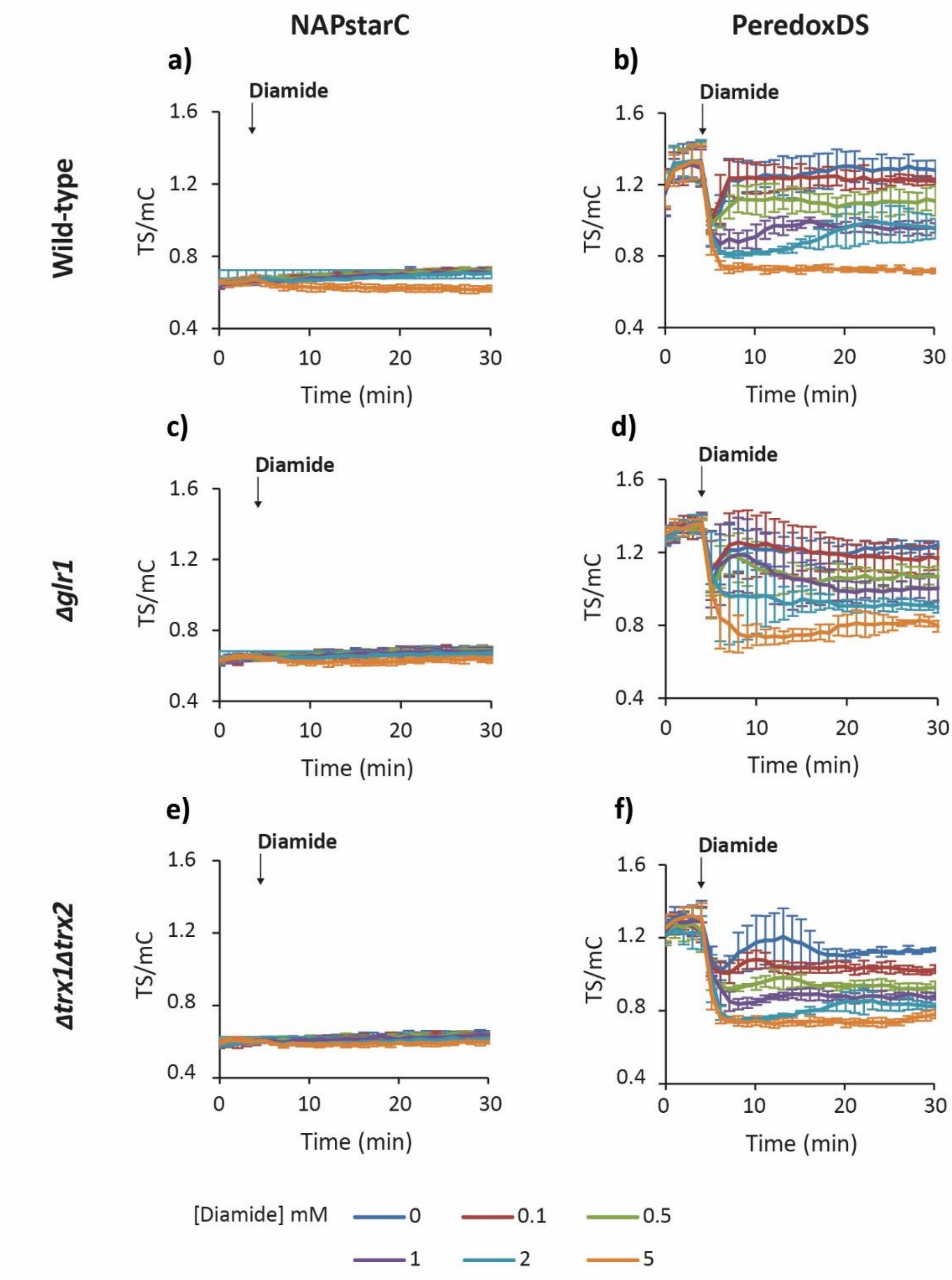

Supplementary Figure 12. Glutathione reductase deletion does not change NAPstarC and PeredoxDS responses to diamide in yeast.

Related to **Figure. 5**. Response of cytosolic NAPstarC or PeredoxDS probes to exogenous diamide at the indicated concentrations in wild-type (**a, b**), Δ*glr1* (**c, d**), and Δ*trx1*Δ*trx2* (**e, f**) cells (n=3 measurements made with cells derived from independent cultures). In all panels, data are presented as mean ± s.d. Identical experimental settings were used for all panels allowing for direct comparison of TS/mC between datasets.

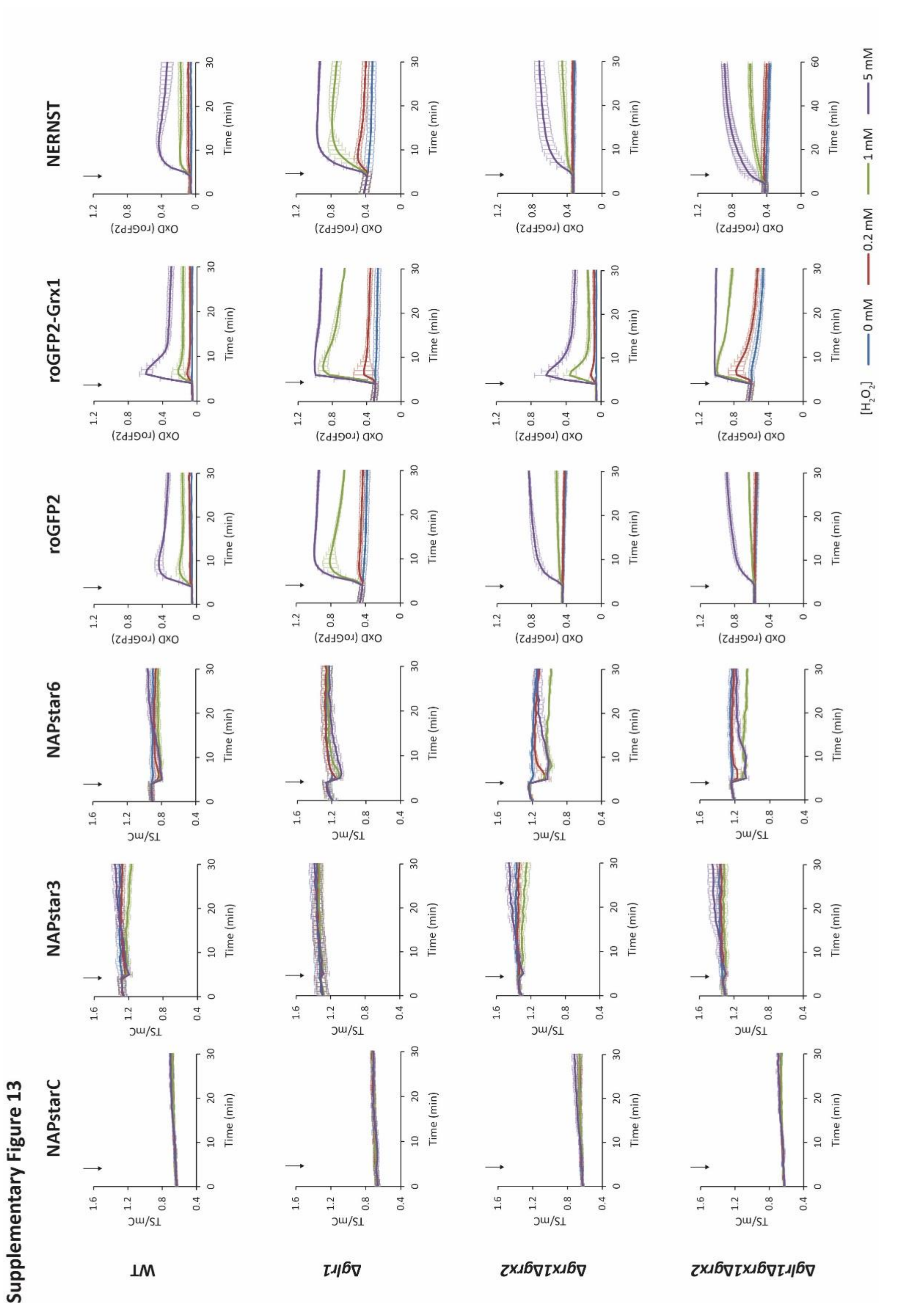

Supplementary Figure 13. NERNST resembles the dynamics of Grx1-roGFP2 in yeast when the NADP and the glutathione redox systems are uncoupled by the absence of glutathione reductase.

Related to **Figure 5**. Response of cytosolic NAPstarC, NAPstar3, NAPstar6, roGFP2, roGFP2-Grx1 and NERNST probes to exogenous H_2O_2 at the indicated concentrations in wild-type, Δ*glr1*, Δ*glr1*Δ*grx1* and Δ*glr1*Δ*grx1*Δ*grx2* cells. (n=3 measurements made with cells derived from independent cultures). In all panels, data are presented as mean ± s.d. Identical experimental settings were used for all panels allowing for direct comparison of TS/mC between datasets.

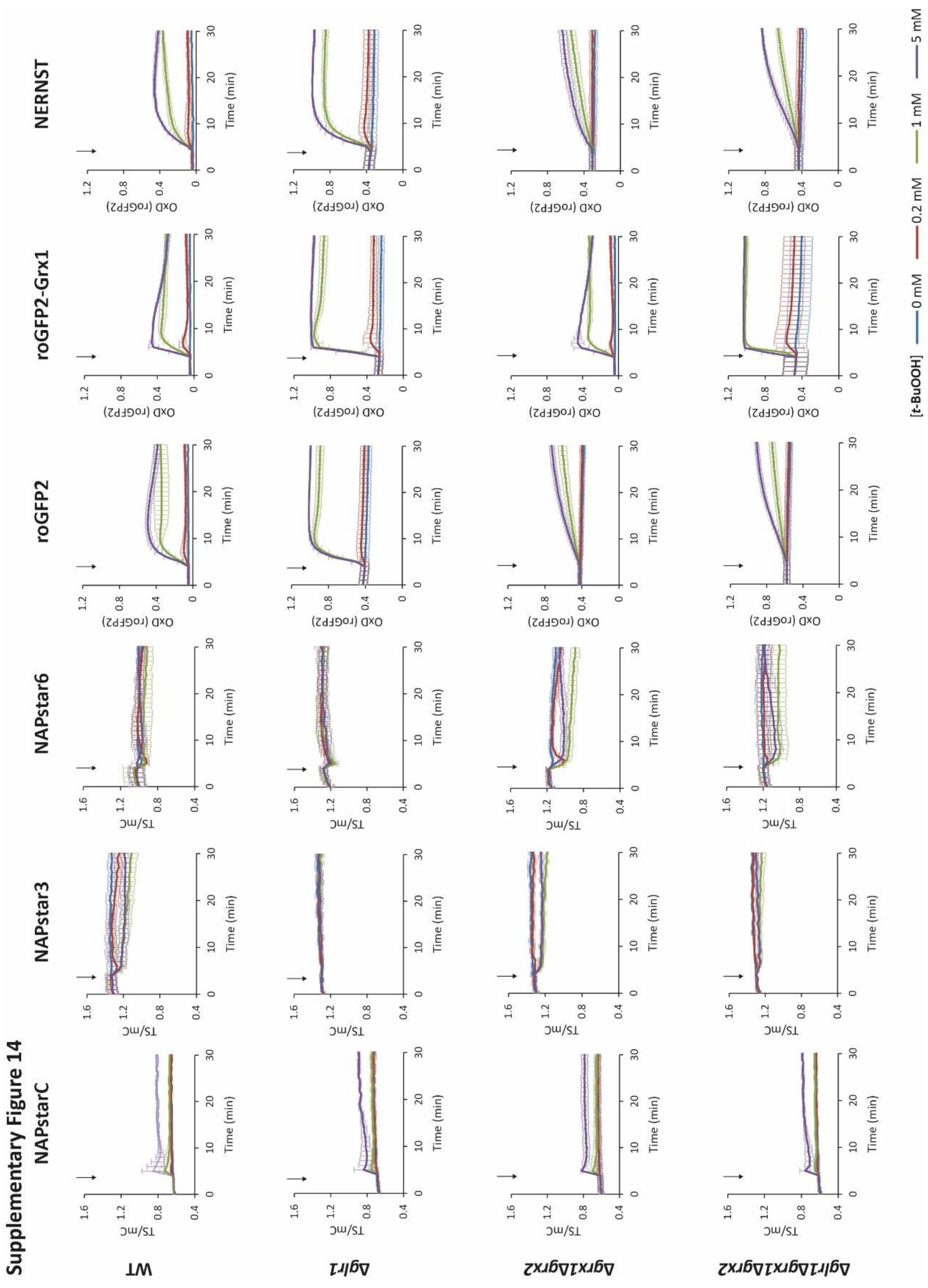

Supplementary Figure 14. NERNST resembles the dynamics of Grx1-roGFP2 in yeast when the NADP and the glutathione redox systems are uncoupled by the absence of glutathione reductase.

Related to **Figure 5**. Response of cytosolic NAPstarC, NAPstar3, NAPstar6, roGFP2, roGFP2-Grx1 and NERNST probes to exogenous *t*-BuOOH at the indicated concentrations in wild-type, Δ*glr1*, Δ*glr1*Δ*grx1* and Δ*glr1*Δ*grx1*Δ*grx2* cells. (n=3 measurements made with cells derived from independent cultures). In all panels, data are presented as mean ± s.d. Identical experimental settings were used for all panels allowing for direct comparison of TS/mC between datasets.

Supplementary Figure 15

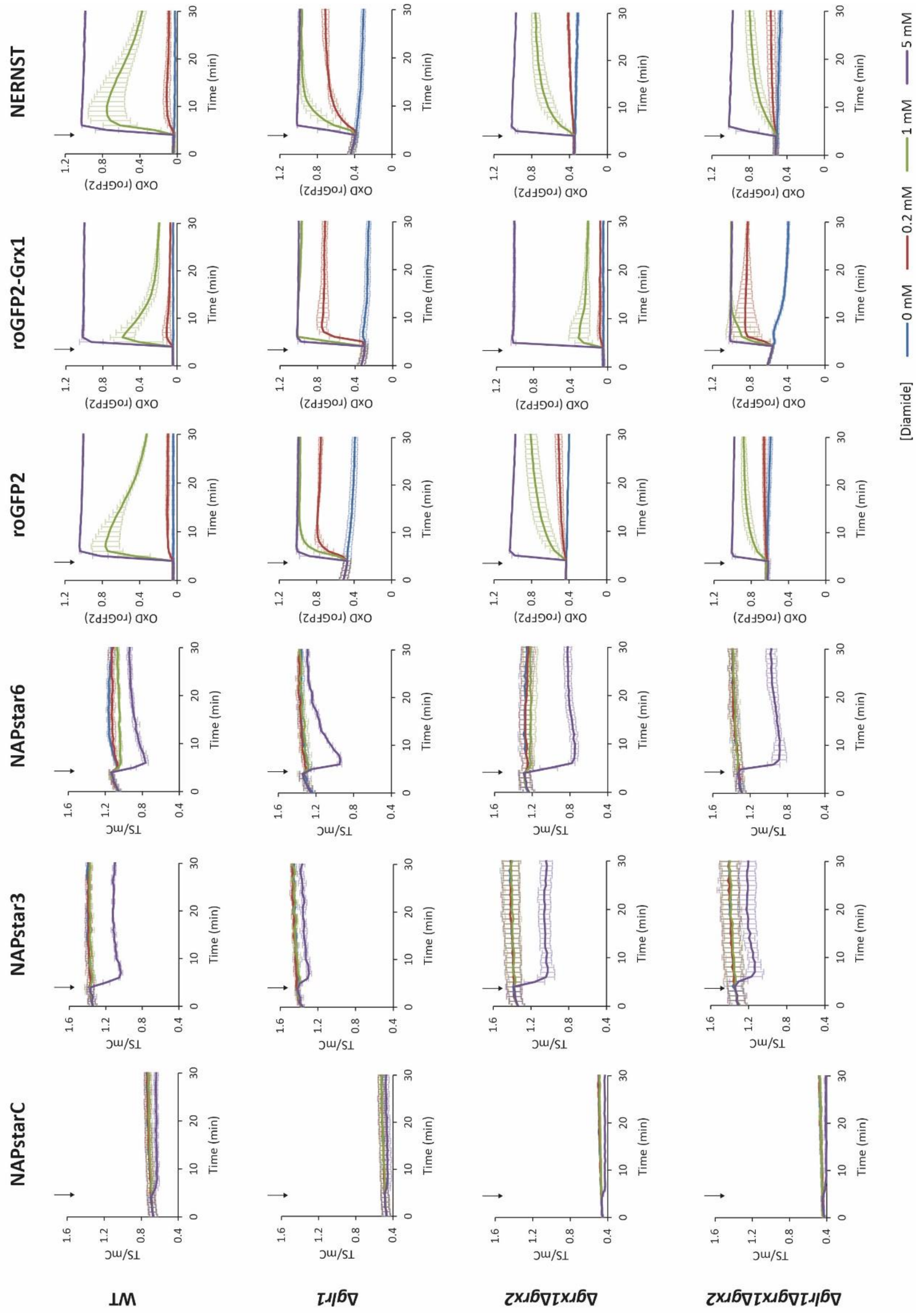

Supplementary Figure 15. NERNST resembles the dynamics of Grx1-roGFP2 in yeast when the NADP and the glutathione redox systems are uncoupled by the absence of glutathione reductase.

Related to **Figure 5**. Response of cytosolic NAPstarC, NAPstar3, NAPstar6, roGFP2, roGFP2-Grx1 and NERNST probes to exogenous diamide at the indicated concentrations in wild-type, Δ*glr1*, Δ*glr1*Δ*grx1* and Δ*glr1*Δ*grx1*Δ*grx2* cells. (n=3 measurements made with cells derived from independent cultures). In all panels, data are presented as mean ± s.d. Identical experimental settings were used for all panels allowing for direct comparison of TS/mC between datasets.

Supplementary Figure 16

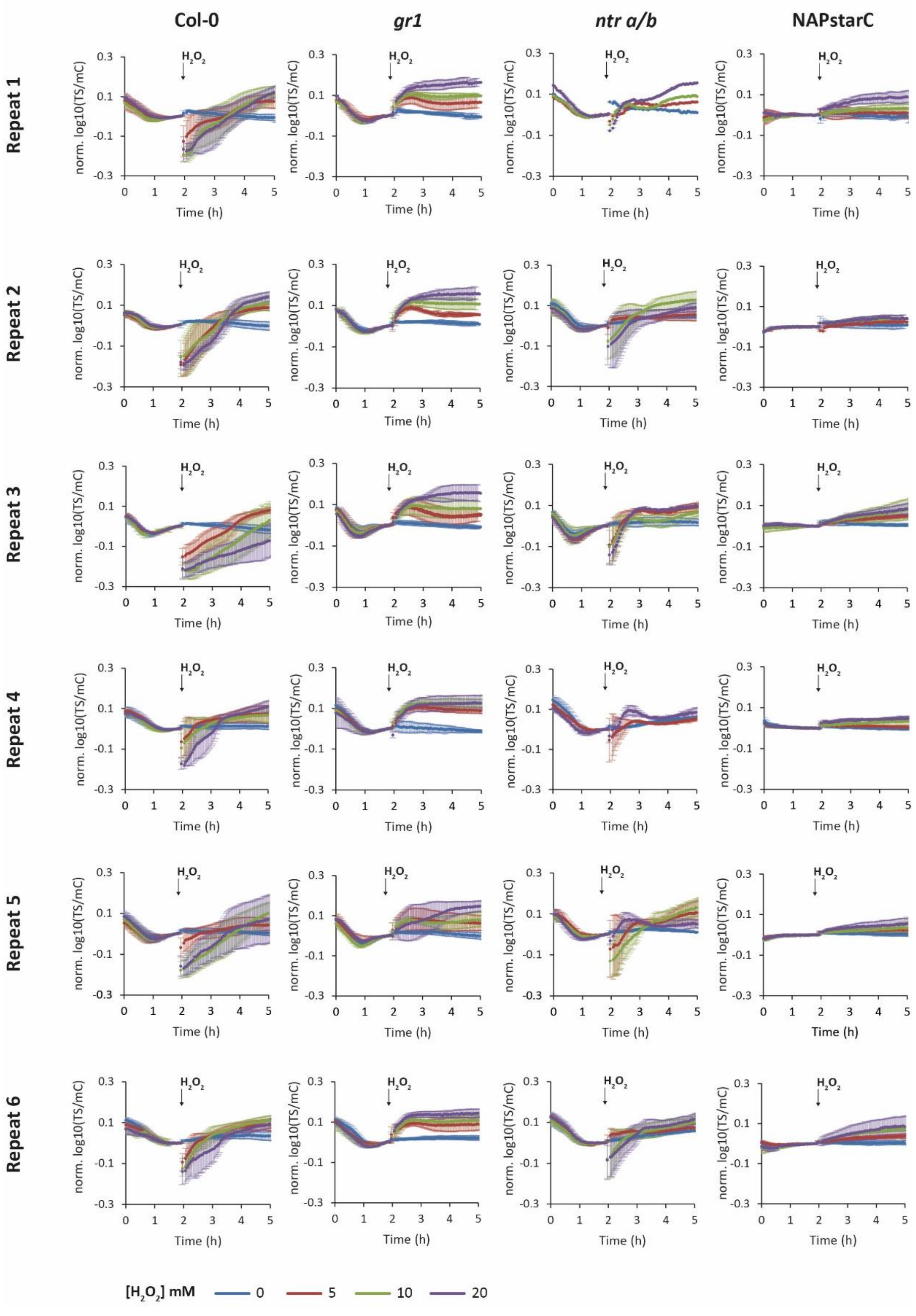

Supplementary Figure 16. Glutathione reductase deletion protects against H_2O_2-induced NADP oxidation in plants.

Related to **Figure. 6**. All six individual experimental repeats of the data in **Fig. 6b–e**. In all panels, data are presented as mean ± s.d. Data were normalised to the average before the treatment.

Supplementary Figure 17

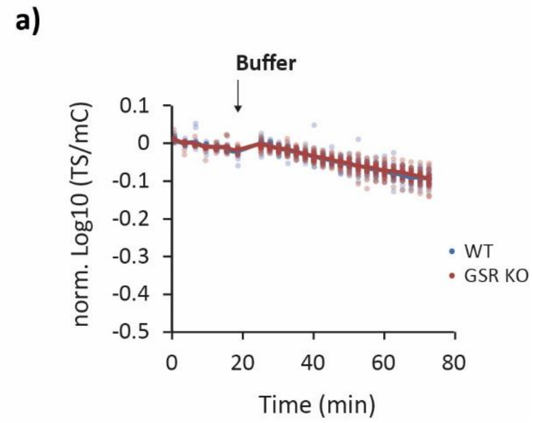

Supplementary Figure 17. Glutathione reductase deletion protects against H_2O_2-induced NADP oxidation in HEK293 cells.

Related to **Figure 7.** Response of NAPstar3b expressed in the cytosol of wild-type and glutathione reductase deleted (GSR KO) HEK293 cells to the buffer control (n=26 individual cells measured in two independent experimental repeats for both wild-type and GSR KO cells).

Supplementary Table 1: Plasmids generated for cloning.

Backbone	Sensor	Generated by
pDONR207	NAPstarC	GeneScript Biotech, Rijswijk, Netherlands
pDONR207	NAPstar1	GeneScript Biotech, Rijswijk, Netherlands
pDONR207	NAPstar2	GeneScript Biotech, Rijswijk, Netherlands
pDONR207	NAPstar3	GeneScript Biotech, Rijswijk, Netherlands
pDONR207	NAPstar4	GeneScript Biotech, Rijswijk, Netherlands
pDONR207	NAPstar3b	Site-directed mutagenesis
pDONR207	NAPstar6	GeneScript Biotech, Rijswijk, Netherlands
pDONR207	NAPstar7	GeneScript Biotech, Rijswijk, Netherlands
pDONR207	NAPstar4.3	*ApaI* and *HindIII* digest

Supplementary Table 2: Plasmids generated for plant expression.

Backbone	Sensor	Generated by
pSS02	NAPstarC	Gateway Cloning
pSS02	NAPstar1	Gateway Cloning
pSS02	NAPstar2	Gateway Cloning
pSS02	NAPstar3	Gateway Cloning
pSS02	NAPstar3b	Gateway Cloning
pSS02	NAPstar4	Gateway Cloning
pSS02	NAPstar6	Gateway Cloning
pSS02	NAPstar7	Gateway Cloning
pSS02	NAPstar4.3	Gateway Cloning

Supplementary Table 3: Plasmids generated for bacterial expression.

Backbone	Sensor	Generated by
pETG10a	NAPstarC	Gateway Cloning
pETG10a	NAPstar1	Gateway Cloning
pETG10a	NAPstar2	Gateway Cloning
pETG10a	NAPstar3	Gateway Cloning
pETG10a	NAPstar3b	Gateway Cloning
pETG10a	NAPstar4	Gateway Cloning
pETG10a	NAPstar6	Gateway Cloning
pETG10a	NAPstar7	Gateway Cloning
pETG10a	NAPstar4.3	Gateway Cloning

Supplementary Table 4: Plasmids generated for mammalian expression.

Backbone	Sensor	Generated by
pcDNA3.1(+)	HyPer7	GeneScript Biotech, Rijswijk, Netherlands
pcDNA3.1(+)	NAPstar3b	GeneScript Biotech, Rijswijk, Netherlands

Supplementary Table 5: Plasmids generated for yeast expression.

**Backbone**	**Sensor**	**Generated by**
p413TEF	NAPstarC	GeneScript Biotech, Rijswijk, Netherlands
p413TEF	NAPstar1	GeneScript Biotech, Rijswijk, Netherlands
p413TEF	NAPstar2	GeneScript Biotech, Rijswijk, Netherlands
p413TEF	NAPstar3	GeneScript Biotech, Rijswijk, Netherlands
p413TEF	NAPstar4	GeneScript Biotech, Rijswijk, Netherlands
p413TEF	NAPstar6	GeneScript Biotech, Rijswijk, Netherlands
p413TEF	NAPstar7	GeneScript Biotech, Rijswijk, Netherlands
p413TEF	NAPstar4.3	Restriction digest/Ligation
p413TEF	Peredox	GeneScript Biotech, Rijswijk, Netherlands
p413TEF	PeredoxDS	Site-directed mutagenesis
p413TEF	NERNST	GeneScript Biotech, Rijswijk, Netherlands
p413TEF	HyPer7	GeneScript Biotech, Rijswijk, Netherlands
p413TEF	roGFP2-Grx1	*XbaI* and *XhoI* digest

Supplementary Table 6: Primers used in this study.

Number	Primer	Sequence	Purpose	Company
Pr1	NAPstar3b_for	TCGCGGTGGTTATATCGAACACGTCG	Generation of plant/ in vitro NAPstar3B	Merck KGaA, Darmstadt, Germany
Pr2	NAPstar3b_rev	ACCGGCCGACCAACCTTC	Generation of plant/ in vitro NAPstar3B	Merck KGaA, Darmstadt, Germany
Pr3	Peredox_D117S_fwd	GAATTGAGAGGTTTCTTTTCCGTTGATC CAGGCATGGTTGGTAGACC	Mutagenesis for generation of PeredoxDS	Eurofins Genomics GmbH, Ebersberg, Germany
Pr4	Peredox_D117S_rev	GGTCTACCAACCATGCCTGGATCAACG GAAAAGAAACCTCTCAATTC	Mutagenesis for generation of PeredoxDS	Eurofins Genomics GmbH, Ebersberg, Germany
Pr5	Peredox_D574S_fwd	CATTTGAATTAAGAGGTTTCTTTTCCGT TGATCCAGGCATGGTCGG	Mutagenesis for generation of PeredoxDS	Eurofins Genomics GmbH, Ebersberg, Germany
Pr6	Peredox_D574S_rev	CCGACCATGCCTGGATCAACGGAAAAG AAACCTCTTAATTCAAATG	Mutagenesis for generation of PeredoxDS	Eurofins Genomics GmbH, Ebersberg, Germany
Pr7	S1_*ZWF1*_fwd	AGTAAATCCAATAGAATAGAAAACCAC ATAAGGCAAGATGcgtacgctgcaggtcgac	Deletion of *ZWF1* in BY4742	Eurofins Genomics GmbH, Ebersberg, Germany
Pr8	S2_*ZWF1*_rev	AGTGACTTAGCCGATAAATGAATGTGC TTGCATTTTTCTAatcgatgaattcgagctcg	Deletion of *ZWF1* in BY4742	Eurofins Genomics GmbH, Ebersberg, Germany
Pr9	S1_*TRX1*_fwd	TTAGTGTAATAGAAGACTAGACACCTC GATACAAATAATGcgtacgctgcaggtcgac	Deletion of *TRX1* in BY4742	Eurofins Genomics GmbH, Ebersberg, Germany
Pr10	S2_*TRX1*_rev	CAGTATAGAAACACAATATATCGGTCA TTGGGTGAGTTTAatcgatgaattcgagctcg	Deletion of *TRX1* in BY4742	Eurofins Genomics GmbH, Ebersberg, Germany
Pr11	S1_*TRX2*_fwd	ACGAGAGTCTACGATATCTTTAAATAAC ACATCAATAATGcgtacgctgcaggtcgac	Deletion of *TRX2* in BY4742	Eurofins Genomics GmbH, Ebersberg, Germany
Pr12	S2_*TRX2*_rev	ACATGATGTACTTTACGTAGCGTTAATA TACCGGCAACTAatcgatgaattcgagctcg	Deletion of *TRX2* in BY4742	Eurofins Genomics GmbH, Ebersberg, Germany
Pr13	S1_*HIS3*_fwd	ACTAAAAAATGAGCAGGCAAGATAAAC GAAGGCAAAGATGcgtacgctgcaggtcgac	Deletion of *HIS3* in CEN.PK113-1A	Merck KGaA, Darmstadt, Germany
Pr14	S2_*HIS3*_rev	ATATATCGTATGCTGCAGCTTTAAATAA TCGGTGTCACTAatcgatgaattcgagctcg	Deletion of *HIS3* in CEN.PK113-1A	Merck KGaA, Darmstadt, Germany

Supplementary Table 7: Yeast strains used in this study.

Genotype	Source
BY4742 *MATα his3Δ1 leu2Δ1 lys2Δ0 ura3Δ0*	Euroscarf
BY4742+p413TEF empty	This study
BY4742+p413TEF PeredoxDS	This study
BY4742+p413TEF NAPstar1	This study
BY4742+p413TEF NAPstar2	This study
BY4742+p413TEF NAPstar3	This study
BY4742+p413TEF NAPstar6	This study
BY4742+p413TEF NAPstar7	This study
BY4742+p413TEF NAPstarC	This study
BY4742+p413TEF roGFP2-Grx1	This study
BY4742+p413TEF NERNST	This study
BY4742 Δ*zwf1*::*natNT2*	This study
BY4742 Δ*zwf1*+p413TEF empty	This study
BY4742 Δ*zwf1*+p413TEF PeredoxDS	This study
BY4742 Δ*zwf1* +p413TEF NAPstar1	This study
BY4742 Δ*zwf1*+p413TEF NAPstar2	This study
BY4742 Δ*zwf1* +p413TEF NAPstar3	This study
BY4742 Δ*zwf1* +p413TEF NAPstar6	This study
BY4742 Δ*zwf1* +p413TEF NAPstar7	This study
BY4742 Δ*zwf1* +p413TEF NAPstarC	This study
BY4742 Δ*glr1*::*kanMX4*	Morgan et al., 2013
BY4742 Δ*glr1* +p413TEF empty	This study
BY4742 Δ*glr1* +p413TEF PeredoxDS	This study
BY4742 Δ*glr1* +p413TEF NAPstar3	This study
BY4742 Δ*glr1* +p413TEF NAPstarC	This study
BY4742 Δ*glr1* +p413TEF roGFP2-Grx1	This study
BY4742 Δ*glr1* +p413TEF NERNST	This study
BY4742 *trx1*::*kanMX4* Δ*trx2*::*natNT2*	This study
BY4742+p413TEF empty	This study
BY4742+p413TEF PeredoxDS	This study
BY4742+p413TEF NAPstar3	This study
BY4742+p413TEF NAPstarC	This study
CEN.PK113-1A	Kind gift from P. Kötter, Frankfurt
CEN.PK113-1AΔ*his3::hphNTI*	This study
CEN.PK113-1AΔ*his3* +p413TEF NAPstar4.3	This study
CEN.PK113-1AΔ*his3* +p413TEF Peredox	This study
CEN.PK113-1AΔ*his3* +p413TEF HyPer7	This study

NAPstar amino acid sequences.

Colour code in the NAPstar amino acid sequences:

Rex domain amino acids

Rex nucleotide-binding site mutations

SAAGGHG = Linker sequences between the first, N-terminal Rex domain and cpTS

T = Linker sequence between cpT-Sapphire and the second, C-terminal Rex domain

GSGTGGNASDGGGSGG = Linker sequence between the C-terminal Rex domain and mCherry

Sequence for cpT-Sapphire

Sequence for mCherry

(GS) is only present in yeast codon-optimised NAPstar constructs. It encodes a BamHI (GGATCC) restriction site arising from the cloning procedure

(VAS) amino acids only present in the bacterial and plant codon-optimised NAPstar constructs.

NAPstar1:

M(GS/VAS)KVPEAAISRLITYLRILEELEAQGVHRTASEQLGELAQVTAFQVDKDLSYFGSYGTDGVGYTVPVLKRELRHILGLNRKWGLCIVGM
GRLGSALADWPGFGESFELRGFFSRSAQKVGRPVRGGVIEHVDLLPQRVPGRIEIALLTVPREAAQKAADLLVAAGIKGILNFAPVVLEVPKEVA
VENVDILAGLTRLSFAILNPTWSAAGGHGFTAHNVYIMADKQKNGIKANFKIRHNIEDGGVQLADHYQQNTPIGDGPVLLPDNHYLSIQSKLSK
DPNEKRDHMVLLEFVTAAGITHGMDELYKGGTGGSMVSKGEELFTGVVPILVELDGDVNGHKFSVSGEGEGDATYGKLTLKFICTTGKLPVPW
PTLVTTFSYGVMVFARYPDHMKQHDFFKSAMPEGYVQERTIFFKDDGNYKTRAEVKFEGDTLVNRIELKGIDFKEDGNILGHKLEYNTKVPEAA
ISRLITYLRILEELEAQGVHRTASEQLGELAQVTAFQVDEDLSYFGSYGTDGVGYTVPVLKRELRHILGLNRKWGLCIVGMGRLGSALADWPGFG
ESFELRGFFSRSAQKVGRPVRGGVIEHVDLLPQRVPGRIEIALLTVPREAAQKAADLLVAAGIKGILNFAPVVLEVPKEVAVENVDFLAGLTRLSF
AILNPKWREEMMGSGTGGNASDGGGSGGMVSKGEEDNMAIIKEFMRFKVHMEGSVNGHEFEIEGEGEGRPYEGTQTAKLKVTKGGPLPFA
WDILSPQFMYGSKAYVKHPADIPDYLKLSFPEGFKWERVMNFEDGGVVTVTQDSSLQDGEFIYKVKLRGTNFPSDGPVMQKKTMGWEASSE
RMYPEDGALKGEIKQRLKLKDGGHYDAEVKTTYKAKKPVQLPGAYNVNIKLDITSHNEDYTIVEQYERAEGRHSTGGMDELYK*

NAPstar2:

M(GS/VAS)KVPEAAISRLITYLRILEELEAQGVHRTASEQLGELAQVTAFQVDKDLSYFGSYGTDGVGYTVPVLKRELRHILGLNRKWGLCIVGM
GRLGSALADWPGFGESFELRGFFSRSAEKVGRPVRGGVIEHTDLLPQRVPGRIEIALLTVPREAAQKAADLLVAAGIKGILNFAPVVLEVPKEVA
VENVDILAGLTRLSFAILNPTWSAAGGHGFTAHNVYIMADKQKNGIKANFKIRHNIEDGGVQLADHYQQNTPIGDGPVLLPDNHYLSIQSKLSK
DPNEKRDHMVLLEFVTAAGITHGMDELYKGGTGGSMVSKGEELFTGVVPILVELDGDVNGHKFSVSGEGEGDATYGKLTLKFICTTGKLPVPW
PTLVTTFSYGVMVFARYPDHMKQHDFFKSAMPEGYVQERTIFFKDDGNYKTRAEVKFEGDTLVNRIELKGIDFKEDGNILGHKLEYNTKVPEAA
ISRLITYLRILEELEAQGVHRTASEQLGELAQVTAFQVDEDLSYFGSYGTDGVGYTVPVLKRELRHILGLNRKWGLCIVGMGRLGSALADWPGFG
ESFELRGFFSRSAEKVGRPVRGGVIEHTDLLPQRVPGRIEIALLTVPREAAQKAADLLVAAGIKGILNFAPVVLEVPKEVAVENVDFLAGLTRLSFA
ILNPKWREEMMGSGTGGNASDGGGSGGMVSKGEEDNMAIIKEFMRFKVHMEGSVNGHEFEIEGEGEGRPYEGTQTAKLKVTKGGPLPFA
WDILSPQFMYGSKAYVKHPADIPDYLKLSFPEGFKWERVMNFEDGGVVTVTQDSSLQDGEFIYKVKLRGTNFPSDGPVMQKKTMGWEASSE
RMYPEDGALKGEIKQRLKLKDGGHYDAEVKTTYKAKKPVQLPGAYNVNIKLDITSHNEDYTIVEQYERAEGRHSTGGMDELYK*

NAPstar3:

M(GS/VAS)KVPEAAISRLITYLRILEELEAQGVHRTASEQLGELAQVTAFQVDKDLSYFGSYGTDGVGYTVPVLKRELRHILGLNRKWGLCIVGM
GRLGSALADWPGFGESFELRGFFSRKAEKVGRPVRGGVIEHYDLLPQRVPGRIEIALLTVPREAAQKAADLLVAAGIKGILNFAPVVLEVPKEVA
VENVDILAGLTRLSFAILNPTWSAAGGHGFTAHNVYIMADKQKNGIKANFKIRHNIEDGGVQLADHYQQNTPIGDGPVLLPDNHYLSIQSKLSK
DPNEKRDHMVLLEFVTAAGITHGMDELYKGGTGGSMVSKGEELFTGVVPILVELDGDVNGHKFSVSGEGEGDATYGKLTLKFICTTGKLPVPW
PTLVTTFSYGVMVFARYPDHMKQHDFFKSAMPEGYVQERTIFFKDDGNYKTRAEVKFEGDTLVNRIELKGIDFKEDGNILGHKLEYNTKVPEAA
ISRLITYLRILEELEAQGVHRTASEQLGELAQVTAFQVDEDLSYFGSYGTDGVGYTVPVLKRELRHILGLNRKWGLCIVGMGRLGSALADWPGFG
ESFELRGFFSRKAEKVGRPVRGGVIEHYDLLPQRVPGRIEIALLTVPREAAQKAADLLVAAGIKGILNFAPVVLEVPKEVAVENVDFLAGLTRLSFA
ILNPKWREEMMGSGTGGNASDGGGSGGMVSKGEEDNMAIIKEFMRFKVHMEGSVNGHEFEIEGEGEGRPYEGTQTAKLKVTKGGPLPFA
WDILSPQFMYGSKAYVKHPADIPDYLKLSFPEGFKWERVMNFEDGGVVTVTQDSSLQDGEFIYKVKLRGTNFPSDGPVMQKKTMGWEASSE
RMYPEDGALKGEIKQRLKLKDGGHYDAEVKTTYKAKKPVQLPGAYNVNIKLDITSHNEDYTIVEQYERAEGRHSTGGMDELYK*

NAPstar4:

M(GS/VAS)KVPEAAISRLITYLRILEELEAQGVHRTASEQLGELAQVTAFQVDKDLSYFGSYGTDGVGYTVPVLKRELRHILGLNRKWGLCIVGM
GDLGSALADWPGFGESFELRGFFSRSAQKVGRPVRGGVIEHVDLLPQRVPGRIEIALLTVPREAAQKAADLLVAAGIKGILNFAPVVLEVPKEVA
VENVDILAGLTRLSFAILNPTWSAAGGHGFTAHNVYIMADKQKNGIKANFKIRHNIEDGGVQLADHYQQNTPIGDGPVLLPDNHYLSIQSKLSK
DPNEKRDHMVLLEFVTAAGITHGMDELYKGGTGGSMVSKGEELFTGVVPILVELDGDVNGHKFSVSGEGEGDATYGKLTLKFICTTGKLPVPW
PTLVTTFSYGVMVFARYPDHMKQHDFFKSAMPEGYVQERTIFFKDDGNYKTRAEVKFEGDTLVNRIELKGIDFKEDGNILGHKLEYNTKVPEAA
ISRLITYLRILEELEAQGVHRTASEQLGELAQVTAFQVDEDLSYFGSYGTDGVGYTVPVLKRELRHILGLNRKWGLCIVGMGDLGSALADWPGF
GESFELRGFFSRSAQKVGRPVRGGVIEHVDLLPQRVPGRIEIALLTVPREAAQKAADLLVAAGIKGILNFAPVVLEVPKEVAVENVDFLAGLTRLS
FAILNPKWREEMMGSGTGGNASDGGGSGGMVSKGEEDNMAIIKEFMRFKVHMEGSVNGHEFEIEGEGEGRPYEGTQTAKLKVTKGGPLPF
AWDILSPQFMYGSKAYVKHPADIPDYLKLSFPEGFKWERVMNFEDGGVVTVTQDSSLQDGEFIYKVKLRGTNFPSDGPVMQKKTMGWEASS
ERMYPEDGALKGEIKQRLKLKDGGHYDAEVKTTYKAKKPVQLPGAYNVNIKLDITSHNEDYTIVEQYERAEGRHSTGGMDELYK*

NAPstar6:

M(GS/VAS)KVPEAAISRLITYLRILEELEAQGVHRTASEQLGELAQVTAFQVDKDLSYFGSYGTDGVGYTVPVLKRELRHILGLNRKWGLCIVGM
GRLGSALADWPGFGESFELRGFFSRKAEKVGRPVRGGVIEHVDLLPQRVPGRIEIALLTAPREAAQKAADLLVAAGIKGILNFAPVVLEVPKEVA
VENVDILAGLTRLSFAILNPTWSAAGGHGFTAHNVYIMADKQKNGIKANFKIRHNIEDGGVQLADHYQQNTPIGDGPVLLPDNHYLSIQSKLSK
DPNEKRDHMVLLEFVTAAGITHGMDELYKGGTGGSMVSKGEELFTGVVPILVELDGDVNGHKFSVSGEGEGDATYGKLTLKFICTTGKLPVPW
PTLVTTFSYGVMVFARYPDHMKQHDFFKSAMPEGYVQERTIFFKDDGNYKTRAEVKFEGDTLVNRIELKGIDFKEDGNILGHKLEYNTKVPEAA
ISRLITYLRILEELEAQGVHRTASEQLGELAQVTAFQVDEDLSYFGSYGTDGVGYTVPVLKRELRHILGLNRKWGLCIVGMGRLGSALADWPGFG
ESFELRGFFSRKAEKVGRPVRGGVIEHVDLLPQRVPGRIEIALLTAPREAAQKAADLLVAAGIKGILNFAPVVLEVPKEVAVENVDFLAGLTRLSFA
ILNPKWREEMMGSGTGGNASDGGGSGGMVSKGEEDNMAIIKEFMRFKVHMEGSVNGHEFEIEGEGEGRPYEGTQTAKLKVTKGGPLPFA
WDILSPQFMYGSKAYVKHPADIPDYLKLSFPEGFKWERVMNFEDGGVVTVTQDSSLQDGEFIYKVKLRGTNFPSDGPVMQKKTMGWEASSE
RMYPEDGALKGEIKQRLKLKDGGHYDAEVKTTYKAKKPVQLPGAYNVNIKLDITSHNEDYTIVEQYERAEGRHSTGGMDELYK*

NAPstar7:

M(GS/VAS)KVPEAAISRLITYLRILEELEAQGVHRTASEQLGELAQVTAFQVDKDLSYFGSYGTDGVGYTVPVLKRELRHILGLNRKWGLCIVGM
GRLGSALADWPGFGESFELRGFFSRKAEKVGRPVRGGVIEHVDLLPQRVPGRIEIALLTTPREAAQKAADLLVAAGIKGILNFAPVVLEVPKEVA
VENVDILAGLTRLSFAILNPTWSAAGGHGFTAHNVYIMADKQKNGIKANFKIRHNIEDGGVQLADHYQQNTPIGDGPVLLPDNHYLSIQSKLSK
DPNEKRDHMVLLEFVTAAGITHGMDELYKGGTGGSMVSKGEELFTGVVPILVELDGDVNGHKFSVSGEGEGDATYGKLTLKFICTTGKLPVPW
PTLVTTFSYGVMVFARYPDHMKQHDFFKSAMPEGYVQERTIFFKDDGNYKTRAEVKFEGDTLVNRIELKGIDFKEDGNILGHKLEYNTKVPEAA
ISRLITYLRILEELEAQGVHRTASEQLGELAQVTAFQVDEDLSYFGSYGTDGVGYTVPVLKRELRHILGLNRKWGLCIVGMGRLGSALADWPGFG
ESFELRGFFSRKAEKVGRPVRGGVIEHVDLLPQRVPGRIEIALLTTPREAAQKAADLLVAAGIKGILNFAPVVLEVPKEVAVENVDFLAGLTRLSFA
ILNPKWREEMMGSGTGGNASDGGGSGGMVSKGEEDNMAIIKEFMRFKVHMEGSVNGHEFEIEGEGEGRPYEGTQTAKLKVTKGGPLPFA
WDILSPQFMYGSKAYVKHPADIPDYLKLSFPEGFKWERVMNFEDGGVVTVTQDSSLQDGEFIYKVKLRGTNFPSDGPVMQKKTMGWEASSE
RMYPEDGALKGEIKQRLKLKDGGHYDAEVKTTYKAKKPVQLPGAYNVNIKLDITSHNEDYTIVEQYERAEGRHSTGGMDELYK*

NAPstar3b:

MKVPEAAISRLITYLRILEELEAQGVHRTASEQLGELAQVTAFQVDKDLSYFGSYGTDGVGYTVPVLKRELRHILGLNRKWGLCIVGMGRLGSAL
ADWPGFGESFELRGFFSRKAEKVGRPVRGGYIEHVDLLPQRVPGRIEIALLTVPREAAQKAADLLVAAGIKGILNFAPVVLEVPKEVAVENVDILA
GLTRLSFAILNPTWSAAGGHGFTAHNVYIMADKQKNGIKANFKIRHNIEDGGVQLADHYQQNTPIGDGPVLLPDNHYLSIQSKLSKDPNEKRD
HMVLLEFVTAAGITHGMDELYKGGTGGSMVSKGEELFTGVVPILVELDGDVNGHKFSVSGEGEGDATYGKLTLKFICTTGKLPVPWPTLVTTFS
YGVMVFARYPDHMKQHDFFKSAMPEGYVQERTIFFKDDGNYKTRAEVKFEGDTLVNRIELKGIDFKEDGNILGHKLEYNTKVPEAAISRLITYLR
ILEELEAQGVHRTASEQLGELAQVTAFQVDEDLSYFGSYGTDGVGYTVPVLKRELRHILGLNRKWGLCIVGMGRLGSALADWPGFGESFELRG
FFSRKAEKVGRPVRGGVIEHYDLLPQRVPGRIEIALLTVPREAAQKAADLLVAAGIKGILNFAPVVLEVPKEVAVENVDFLAGLTRLSFAILNPKW
REEMMGSGTGGNASDGGGSGGMVSKGEEDNMAIIKEFMRFKVHMEGSVNGHEFEIEGEGEGRPYEGTQTAKLKVTKGGPLPFAWDILSPQ
FMYGSKAYVKHPADIPDYLKLSFPEGFKWERVMNFEDGGVVTVTQDSSLQDGEFIYKVKLRGTNFPSDGPVMQKKTMGWEASSERMYPED
GALKGEIKQRLKLKDGGHYDAEVKTTYKAKKPVQLPGAYNVNIKLDITSHNEDYTIVEQYERAEGRHSTGGMDELYK*

NAPstar4.3:

M(GS/VAS)KVPEAAISRLITYLRILEELEAQGVHRTASEQLGELAQVTAFQVDKDLSYFGSYGTDGVGYTVPVLKRELRHILGLNRKWGLCIVGM
GDLGSALADWPGFGESFELRGFFSRSAQKVGRPVRGGVIEHVDLLPQRVPGRIEIALLTVPREAAQKAADLLVAAGIKGILNFAPVVLEVPKEVA
VENVDILAGLTRLSFAILNPTWSAAGGHGFTAHNVYIMADKQKNGIKANFKIRHNIEDGGVQLADHYQQNTPIGDGPVLLPDNHYLSIQSKLSK
DPNEKRDHMVLLEFVTAAGITHGMDELYKGGTGGSMVSKGEELFTGVVPILVELDGDVNGHKFSVSGEGEGDATYGKLTLKFICTTGKLPVPW
PTLVTTFSYGVMVFARYPDHMKQHDFFKSAMPEGYVQERTIFFKDDGNYKTRAEVKFEGDTLVNRIELKGIDFKEDGNILGHKLEYNTKVPEAA
ISRLITYLRILEELEAQGVHRTASEQLGELAQVTAFQVDEDLSYFGSYGTDGVGYTVPVLKRELRHILGLNRKWGLCIVGMGRLGSALADWPGFG
ESFELRGFFSRKAEKVGRPVRGGVIEHYDLLPQRVPGRIEIALLTVPREAAQKAADLLVAAGIKGILNFAPVVLEVPKEVAVENVDFLAGLTRLSFA
ILNPKWREEMMGSGTGGNASDGGGSGGMVSKGEEDNMAIIKEFMRFKVHMEGSVNGHEFEIEGEGEGRPYEGTQTAKLKVTKGGPLPFA
WDILSPQFMYGSKAYVKHPADIPDYLKLSFPEGFKWERVMNFEDGGVVTVTQDSSLQDGEFIYKVKLRGTNFPSDGPVMQKKTMGWEASSE
RMYPEDGALKGEIKQRLKLKDGGHYDAEVKTTYKAKKPVQLPGAYNVNIKLDITSHNEDYTIVEQYERAEGRHSTGGMDELYK*

NAPstarC:

M(GS/VAS)KVPEAAISRLITYLRILEELEAQGVHRTASEQLGELAQVTAFQVDKDLSYFGSYGTDGVGYTVPVLKRELRHILGLNRKWGLCIVGM
GDLGSALADWPGFGESFELRGFFSVSPEKVGRPVRGGVIEHVDLLPQRVPGRIEIALLTAPREAAQKAADLLVAAGIKGILNFAPVVLEVPKEVA
VENVDILAGLTRLSFAILNPTWSAAGGHGFTAHNVYIMADKQKNGIKANFKIRHNIEDGGVQLADHYQQNTPIGDGPVLLPDNHYLSIQSKLSK
DPNEKRDHMVLLEFVTAAGITHGMDELYKGGTGGSMVSKGEELFTGVVPILVELDGDVNGHKFSVSGEGEGDATYGKLTLKFICTTGKLPVPW
PTLVTTFSYGVMVFARYPDHMKQHDFFKSAMPEGYVQERTIFFKDDGNYKTRAEVKFEGDTLVNRIELKGIDFKEDGNILGHKLEYNTKVPEAA
ISRLITYLRILEELEAQGVHRTASEQLGELAQVTAFQVDEDLSYFGSYGTDGVGYTVPVLKRELRHILGLNRKWGLCIVGMGDLGSALADWPGF
GESFELRGFFSVSPEKVGRPVRGGVIEHVDLLPQRVPGRIEIALLTAPREAAQKAADLLVAAGIKGILNFAPVVLEVPKEVAVENVDFLAGLTRLSF
AILNPKWREEMMGSGTGGNASDGGGSGGMVSKGEEDNMAIIKEFMRFKVHMEGSVNGHEFEIEGEGEGRPYEGTQTAKLKVTKGGPLPFA
WDILSPQFMYGSKAYVKHPADIPDYLKLSFPEGFKWERVMNFEDGGVVTVTQDSSLQDGEFIYKVKLRGTNFPSDGPVMQKKTMGWEASSE
RMYPEDGALKGEIKQRLKLKDGGHYDAEVKTTYKAKKPVQLPGAYNVNIKLDITSHNEDYTIVEQYERAEGRHSTGGMDELYK*
