# Supplementary material for: A family of NADPH/NADP+ biosensors reveals in vivo dynamics of central redox metabolism across eukaryotes
Source: Nat Commun. 2024 Dec 19;15:10704. doi: 10.1038/s41467-024-55302-x (PMC11659435; doi:10.1038/s41467-024-55302-x)
Supplement: Supplementary file 2 — Reporting Summary [file 41467_2024_55302_MOESM2_ESM.pdf]

nature portfolio

Corresponding author(s): Markus Schwarzländer
Bruce Morgan

Last updated by author(s): Nov 21, 2024

Reporting Summary

Nature Portfolio wishes to improve the reproducibility of the work that we publish. This form provides structure for consistency and transparency in reporting. For further information on Nature Portfolio policies, see our Editorial Policies and the Editorial Policy Checklist.

Statistics

For all statistical analyses, confirm that the following items are present in the figure legend, table legend, main text, or Methods section.

n/a	Confirmed	
☐	☒	The exact sample size (*n*) for each experimental group/condition, given as a discrete number and unit of measurement
☐	☒	A statement on whether measurements were taken from distinct samples or whether the same sample was measured repeatedly
☐	☒	The statistical test(s) used AND whether they are one- or two-sided<br>*Only common tests should be described solely by name; describe more complex techniques in the Methods section.*
☐	☒	A description of all covariates tested
☐	☒	A description of any assumptions or corrections, such as tests of normality and adjustment for multiple comparisons
☐	☒	A full description of the statistical parameters including central tendency (e.g. means) or other basic estimates (e.g. regression coefficient) AND variation (e.g. standard deviation) or associated estimates of uncertainty (e.g. confidence intervals)
☐	☒	For null hypothesis testing, the test statistic (e.g. *F*, *t*, *r*) with confidence intervals, effect sizes, degrees of freedom and *P* value noted<br>*Give P values as exact values whenever suitable.*
☒	☐	For Bayesian analysis, information on the choice of priors and Markov chain Monte Carlo settings
☒	☐	For hierarchical and complex designs, identification of the appropriate level for tests and full reporting of outcomes
☒	☐	Estimates of effect sizes (e.g. Cohen's *d*, Pearson's *r*), indicating how they were calculated

Our web collection on statistics for biologists contains articles on many of the points above.

Software and code

Policy information about availability of computer code

Data collection	All data was collected as described in the Materials & methods section
Data analysis	Data analysis and statistical anaylsis was performed using MS Excel 2010 together with the ExcelSolver_Sigmoid plugin. For images analysis, RRA Version 1.3 (Fricker, 2016) was used.

For manuscripts utilizing custom algorithms or software that are central to the research but not yet described in published literature, software must be made available to editors and reviewers. We strongly encourage code deposition in a community repository (e.g. GitHub). See the Nature Portfolio guidelines for submitting code & software for further information.

Data

Policy information about availability of data

All manuscripts must include a data availability statement. This statement should provide the following information, where applicable:

- Accession codes, unique identifiers, or web links for publicly available datasets
- A description of any restrictions on data availability
- For clinical datasets or third party data, please ensure that the statement adheres to our policy

Data representations are included in the article, and the supplemental files and tables. All source and raw data is available upon request. If seen appropriate any source data can be added to the supplement of the manuscript upon editorial or reviewer request.

Research involving human participants, their data, or biological material

Policy information about studies with human participants or human data. See also policy information about sex, gender (identity/presentation), and sexual orientation and race, ethnicity and racism.

Reporting on sex and gender	*Use the terms sex (biological attribute) and gender (shaped by social and cultural circumstances) carefully in order to avoid confusing both terms. Indicate if findings apply to only one sex or gender; describe whether sex and gender were considered in study design; whether sex and/or gender was determined based on self-reporting or assigned and methods used. Provide in the source data disaggregated sex and gender data, where this information has been collected, and if consent has been obtained for sharing of individual-level data; provide overall numbers in this Reporting Summary. Please state if this information has not been collected. Report sex- and gender-based analyses where performed, justify reasons for lack of sex- and gender-based analysis.*
Reporting on race, ethnicity, or other socially relevant groupings	*Please specify the socially constructed or socially relevant categorization variable(s) used in your manuscript and explain why they were used. Please note that such variables should not be used as proxies for other socially constructed/relevant variables (for example, race or ethnicity should not be used as a proxy for socioeconomic status). Provide clear definitions of the relevant terms used, how they were provided (by the participants/respondents, the researchers, or third parties), and the method(s) used to classify people into the different categories (e.g. self-report, census or administrative data, social media data, etc.) Please provide details about how you controlled for confounding variables in your analyses.*
Population characteristics	*Describe the covariate-relevant population characteristics of the human research participants (e.g. age, genotypic information, past and current diagnosis and treatment categories). If you filled out the behavioural & social sciences study design questions and have nothing to add here, write "See above."*
Recruitment	*Describe how participants were recruited. Outline any potential self-selection bias or other biases that may be present and how these are likely to impact results.*
Ethics oversight	*Identify the organization(s) that approved the study protocol.*

Note that full information on the approval of the study protocol must also be provided in the manuscript.

Field-specific reporting

Please select the one below that is the best fit for your research. If you are not sure, read the appropriate sections before making your selection.

[x] Life sciences [ ] Behavioural & social sciences [ ] Ecological, evolutionary & environmental sciences

For a reference copy of the document with all sections, see nature.com/documents/nr-reporting-summary-flat.pdf

Life sciences study design

All studies must disclose on these points even when the disclosure is negative.

Sample size	Sample size was not statistically predetermined. For fluorescence measurements, a standard sample size of n = 3-12 was sufficient as the phenotypes were highly reproducible. For protein metabolite interaction analyses ,the sample size was as big as feasible for our experimental approaches.
Data exclusions	Shown is average data described in the Materials and Methods. Raw data and source data is available upon request and could be added as supplemetal tables if deemed appropriate.
Replication	The analyses were replicated as indicated in the figure legends.
Randomization	All plants used in the study were randomized in the growth chambers to avoid edge effects (variations in light, temperature and humidity).
Blinding	Differences in metabolic behaviour are not apparent from the gross plant phenotype.

Reporting for specific materials, systems and methods

We require information from authors about some types of materials, experimental systems and methods used in many studies. Here, indicate whether each material, system or method listed is relevant to your study. If you are not sure if a list item applies to your research, read the appropriate section before selecting a response.

Materials & experimental systems

n/a	Involved in the study
[ ]	[x] Antibodies
[ ]	[x] Eukaryotic cell lines
[x]	[ ] Palaeontology and archaeology
[x]	[ ] Animals and other organisms
[x]	[ ] Clinical data
[x]	[ ] Dual use research of concern
[ ]	[x] Plants

Methods

n/a	Involved in the study
[x]	[ ] ChIP-seq
[x]	[ ] Flow cytometry
[x]	[ ] MRI-based neuroimaging

Antibodies

Antibodies used

GSR rabbit polyclonal antibody; Proteintech, Cat. Number: 18257-1-AP.

Validation

Validation on the company website was performed by testing the antibody on HeLa cells with an si-RNA knockdown of GSR. In this study, we used the antibody to verify knockout of GSR in HEK293 cells, finding no detectable protein in contrast to HEK293 WT cells where a strong signal was detected.

Eukaryotic cell lines

Policy information about cell lines and Sex and Gender in Research

Cell line source(s)

HeLa were obtained from the DSMZ-German Collection of Microorganisms and Cell Cultures: Number (ACC57).
HEK293 Flp-In™ WT, HEK293 Flp-In™ GSR KO

Authentication

For HeLa cells, no further authentication was performed.
Authentication of the HEK293 Flp-In™ WT and GSR KO cell lines was performed with westernblot analysis, screening for loss of protein using the corresponding antibody (GSR rabbit polyclonal antibody; Proteintech, Cat. Number: 18257-1-AP).

Mycoplasma contamination

HeLa cells were screened 3 times per year for mycoplasma contamination.
HEK293 Flp-In™cell lines were not tested for mycoplasma contamination.

Commonly misidentified lines
(See ICLAC register)

No commonly misidentified cell lines were used in this study.

Plants

Seed stocks

The following seed stocks from public collections were used: gr1 (Marty et al., 2009), SALK_105794; ntra x ntrb (Reichheld et al., 2007), SALK_539152 and SALK_545978, referred to as ntra/b in this manuscript. Sensor lines were either generated as described in the manuscript or used aftre generation in prior work as referred to in the manuscript.

Novel plant genotypes

All novel plant genotypes in the Arabidopsis thaliana Col-0 background were generated by Agrobacterium-mediated transformation using the floal dip method. Details of the constructs that were used for transformation are provided in the Materials & Methods section.

Authentication

Authentification of the plant genotypes was performed by PCR with appropriate primer pairs to detect the specific T-DNA. For the expression of biosensors the fluorescent intensity and spectra were also used for validation.
