# Supplementary material for: A family of NADPH/NADP+ biosensors reveals in vivo dynamics of central redox metabolism across eukaryotes
Source: Nat Commun. 2024 Dec 19;15:10704. doi: 10.1038/s41467-024-55302-x (PMC11659435; doi:10.1038/s41467-024-55302-x)
Supplement: Supplementary file 3 — Transparent Peer Review file [file 41467_2024_55302_MOESM3_ESM.pdf]

nature portfolio

Peer Review File

A family of NADPH-NADP+ biosensors reveals in vivo dynamics of central redox metabolism across eukaryotes

Corresponding Author: Professor Bruce Morgan

This file contains all reviewer reports in order by version, followed by all author rebuttals in order by version.

Version 1:

Reviewer comments:

Reviewer #1

(Remarks to the Author)
NADPH and its oxidation state (NADPH/NADP ratio) are a central feature of metabolism driving biosynthetic reactions and as emphasised in this manuscript, the antioxidant system (as the “ultimate” reductant). In this manuscript the authors report an important advance in designing and expressing a fluorescent biosensor (NAPstar) capable of measuring NADPH/NADP with high specificity and sensitivity. The starting point was the NAD(H) sensor Peredox. In vitro studies with the recombinant NAPs tars show that the sensors have low pH sensitivity and low reactivity with the related NADH/NAD. NAPstars were expressed in the cytosol of yeast, Arabidopsis and HeLa cells to assess functionality across fungi, plants and mammalian cells. An elegant series of experiments with these transformed strains, including perturbations (oxidative stress via H2O2, diamide and hypoxia) combined with other biosensors for GSH redox state and hydrogen peroxide) mutants and inhibitors, assessed the functionality of NAPstar and revealed new information on NADP(H). The NADPH/NADP ratio in the cytosol is lower than suggested from previous measurements. Importantly, the question of the relative role of glutathione and thioredoxins in hydrogen peroxide removal could be investigated, showing that GSH has the more prominent role across kingdoms. Comparison of the response of a previously proposed NADP(H) sensor NERNST with NAPstar and Grx1-roGFP, suggested that it is too influenced by GSH redox state to be reliable for NADPH/NADP measurement. Overall, this manuscript reports an important new tool to probe metabolism and redox /antioxidant processes. It is a highly significant advance of wide interest.

I have two comments which the authors could consider.

Line 482 onwards. I found the discussion/presentation of the NADPH/NADP ratio confusing. It is suggested that NADP is less reduced than suggested by previous measurements, but the authors also show that NAPstar cannot measure ratios above 5. Perhaps this could be clarified by stating the typical ratios found in unperturbed cells more explicitly. The figures for expression in cells show the fluorescence signals and the reader needs to look back at the in vitro data to get an idea of the NADP oxidation state. It would be useful to address this presentational issue in the text or figures.

Arguably, I think it would be desirable to repeat some of the experiments to make actual NADP/NADPH measurements in extracts for comparison with NAPstar. I realise the point of the biosensor is to have specificity for subcellular compartments but, particularly for the GR mutants, it would provide a useful comparison/verification of the trend.

Reviewer #2

(Remarks to the Author)
This manuscript describes the production and application of an NAD redox state sensor, that is based on Peredox-mCherry, in which fluorescence excitation and emission spectra are resistant to pH changes. The authors clearly describe the advantages offered by NAPstars relative to other NADPH-NADP+ biosensors. The possibility of measuring real-time, specific, pH-resistant measurements, across a broad-range of NADP redox states, in different subcellular compartments is certainly an attractive proposition. The authors provide several lines of evidence from different systems using appropriate

approaches to validate the probes used and test robustness in in vitro as well as in vivo systems. However, I cannot see that the authors attempted simultaneous biochemical measurements of NAD(P)Hand NAD(P+). Such measurements would have been insightful not least to determine how the in vivo measurements differ from the classical extraction and assay methods. The proof of the response ranges for both sensor variants for NADPH/NADP+ in in vito systems, does not directly confirm that each sensor accurately measures the physiological NADP redox state.
While the data presented are convincing, only a small amount of data is presented for each system analysed and this tends to open more questions than answers. For example, a microplate-based assay was used to monitor the response of NAPstars and PeredoxDS to the addition of hydrogen peroxide (0–5 mM), when expressed in the cytosol of the budding yeast, Saccharomyces cerevisiae. Only a small response was observed in the probe, the data indicating a limited response with a rapid recovery. Although the observed dynamic changes in pyridine nucleotide redox state that were observed during the yeast metabolic cycle are readily understandable, some of the other responses such as the lack of response to external hydrogen peroxide are less easy to explain. These findings are interesting but not considered in the discussion section.
The studies in illuminated leaf tissue related photosynthesis are particularly interesting, as are the studies on hypoxia, in which there are comparable measurements of the glutathione redox potential using the Grx1-roGFP2 and roGFP2-Orp1 probes. The proposed limitation on glutathione reductase activity under re-oxygenation after hypoxia is insightful, as is the conclusion that the NERNST sensor functions well as a glutathione redox state reporter but does not operate as well for the NADP redox state. However, there is no real discussion of this conclusion or its implications. Although much of the discussion focusses on the relationships between the pyridine nucleotide, glutathione and thioredoxin pools, I struggled to find any real novelty in these findings, which appeared to be largely confirmatory.

Reviewer #3

(Remarks to the Author)
Scherschel and coworkers created a family of NADP+/NADPH redox state sensors (termed “NAPstars”) based on reverse engineering of the NAD-sensitive Peredox sensor and iNAP sensor. The sensors use two copies of the mutated NADH/NAD+-binding domain of Rex bounded by circularly permuted T-Sapphire and mCherry. After characterizing the sensitivity to redox states and pH, the authors apply the sensors in various organisms including yeast, HeLa cells, Arabidopsis thaliana plants, and HEK293 cells. Unfortunately, (1) the rational sensor design is not clearly articulated, (2) the sensor responses do not appear to solely respond to NADPH/NADP redox state and (3) the sensors appear to be pH-dependent in contrast to their conclusion.

Major issues:
(1) The authors first characterized purified sensor responses to changes in NADPH/NADP and NADH/NAD concentration (Fig. 1). Each sensor shows changes in the TS/mCherry emission ratio in response to NADPH/NADP ratio but they only respond to very high NADH/NAD+ ratio. This evidence is used to conclude that the sensors respond to the NADPH/NADP redox state with different sensitivities for eacg sensor (KD(NADPH) and KR(NADPH/NADP)). However, the sensors show very different shapes depending on holding NADPH or NADP constant (i.e., they do not show the same Kr based on comparing Fig. 1c and 1f). What accounts for this difference? Doesn’t this make the sensor response uninterpretable?

(2) The authors explore the pH sensitivity of the sensor (Supplemental Fig. 3). Based on Fig. 1c and Fig. S2, the maximal response of these sensors is around 0.4-0.6 TS/mC units. When measuring the response to varying pH, the authors state that the NAPstar varied “only ~30%” over 3 pH units concluding that the sensor is insensitive to pH (Fig. S3). However, the change across 3 pH units corresponds to ~0.3 TS/mC units or ~50-75% of the total response. Thus, the impact of pH is rather large. Furthermore, the data appear to show pH impacts NADP binding affinity and thus lowering pH would lower the overall ratio. Doesn’t this make the response uninterpretable?

(3) The authors have generated a family of NAPStar sensors but do little to convey how each sensor is designed, unique, and thus useful. They inadequately express/convey the logic behind the various mutations right from the beginning and subsequently do little to convey how each sensor was selected for each experiment. One example (but this occurs throughout) is line 292: “we generated stable transgenic NAPstar3, NAPstar4.3 and NAPstarC lines with cyto-nuclear sensor expression.” Why were these two sensors used? A one-sentence rationale should be stated to allow the reader to understand.

(4) This family of sensors appears to respond to NADPH/NADP ratio between 0.0006 and 0.2842 (Fig. 2h), which by their admission is far from the “textbook” accepted ratios of NADPH/NADP. The authors take this to reinterpret the direction of NADPH -> GSH redox biology (lines 494-500). However, an alternative interpretation is that their sensors respond to gross oxidation, but do not respond to physiologically relevant changes. In support of a limited physiological response, Fig. 2c shows the limited response to H_2O_2 in yeast and Fig. 4b shows the limited response to light of NAPStar4.3 in plants and similar to NAPStarC.

Minor issues:
(1) How are the curves normalized? The normalization appears to jump around from experiment to experiment.

(2) Supplementary Fig 4: There is no consistency in the controls. The control sensor (NAPStarC) is only found in 4a. In referencing, supplemental Fig. 4b the authors state: “Upon the addition of GSSG, we observed a rapid decrease in the TS/mC ratio for each NAPstar construct except NAPstarC.” Yet, NAPStarC is not shown.

(3) Fig. 4: The NAPStarC changes in Fig. 4 were significant from baseline. The fact that the NAPStarC response was

smaller compared to the sensor does not make it “minor”.

(4) Line 202: Supplemental figure 5 states Kd(NADPH) = 40.5 uM yet here says NADH. Which is it?

(5) Fig. 2 and supplemental Fig. 6: Do these cells express catalase which detoxifies H2O2 without using NADPH? Experiments should be redone with tert-Butyl hydroperoxide, which is not detoxified by catalase.

(6) Discussion: “NAPstars exhibit several other advantages over currently available NADP sensors including pH-resistance”. This is not true based on the discussion above. Also, other sensors have shown pH resistance e.g., mTQ2-based Apollo.

(7) Line 276: Do you mean “previously linked” to cell division as I do not see evidence that you measured cell cycle/division?

(8) Line 408: Revise: “thioredoxin reductases ntr a/b or cytosolic glutathione reductase gr1”

Version 2:

Reviewer comments:

Reviewer #1

(Remarks to the Author)
I am happy with the response of the reviewers to my comments and those of the other reviewers. They have made suitable revisions and provided a thoughtful rebuttal. In particular, it was interesting to learn about the unreliability of NADP/H assays of cell and tissue extracts.

Reviewer #2

(Remarks to the Author)
The authors have adequately addressed the points raised.

Reviewer #3

(Remarks to the Author)
Most of my concerns have been addressed.

However, one major concern remains:

Genetically encoded sensors commonly suffer from some level of pH dependence. It is appreciated that the authors provided an extensive characterization of the pH sensitivity of their sensors. However, from this evidence the NAPStar sensors cannot be described as “pH robust”.

The control sensor (NAPStarC) shows a significant response to physiological pH (e.g., 7-8) (Fig. S3). Since the control sensor shows a limited subsequent response to NADP+, the pH response is likely due to the fluorophores or to some sort of structural change in the sensors. The “buffer” responses of all the sensors show a similar pH-dependent shape to NAPStarC (Fig. S3 b-h). However, the subsequent responses to NADP+ all varied depending on the pH. For example, NADPStar3 at pH 7.5 and going from 8 to 100 uM went from ~1.2 to ~1.05 TS/mC units. In contrast, there is no change in the ratio in response to NADP at pH 8.5 (i.e., it goes from ~1 to ~1). This change is large when considering the maximal response of these sensors is around 0.4-0.6 TS/mC units. These varied responses suggest pH-dependence in the NADP and/or NADPH binding affinity, which cannot be fully controlled using the NADPStarC alone.

As stated, the authors have done an excellent job of documenting the pH dependence of these sensors and most sensors suffer from some level of this artifact.

Thus, to rectify this:
(1) The authors need to replace the use of “robust” when describing the pH dependence. For example, the sensor could be described as showing a "moderate" pH dependence that can be in part controlled for using NAPStarC.
(2) The authors also need to explicitly state that the NAPStarC is a control for the pH dependence of the fluorophores and does not fully control for the pH-dependent of the affinity in the binding domains.

Open Access This Peer Review File is licensed under a Creative Commons Attribution 4.0 International License, which permits use, sharing, adaptation, distribution and reproduction in any medium or format, as long as you give appropriate credit to the original author(s) and the source, provide a link to the Creative Commons license, and indicate if changes were made.
In cases where reviewers are anonymous, credit should be given to 'Anonymous Referee' and the source.
The images or other third party material in this Peer Review File are included in the article's Creative Commons license, unless indicated otherwise in a credit line to the material. If material is not included in the article's Creative Commons license and your intended use is not permitted by statutory regulation or exceeds the permitted use, you will need to obtain permission directly from the copyright holder.
To view a copy of this license, visit https://creativecommons.org/licenses/by/4.0/

Response to the Reviewers

We thank all three reviewers for their hugely helpful and thoughtful advice. We are particularly glad for the joint support of our manuscript overall and have done our best to address open points, where possible through additional empirical testing. We respond to the comments point-by-point. Individual responses to specific points are more extensive than usual. However, we deemed them extremely interesting and although they cannot be sufficiently discussed in their full context here, we have been aiming to provide a realistic representation of our thoughts.

REVIEWER COMMENTS

Reviewer #1 (Remarks to the Author):

NADPH and its oxidation state (NADPH/NADP ratio) are a central feature of metabolism driving biosynthetic reactions and as emphasised in this manuscript, the antioxidant system (as the "ultimate" reductant). In this manuscript the authors report an important advance in designing and expressing a fluorescent biosensor (NAPstar) capable of measuring NADPH/NADP with high specificity and sensitivity. The starting point was the NAD(H) sensor Peredox. In vitro studies with the recombinant NAPs tars show that the sensors have low pH sensitivity and low reactivity with the related NADH/NAD. NAPstars were expressed in the cytosol of yeast, Arabidopsis and HeLa cells to assess functionality across fungi, plants and mammalian cells. An elegant series of experiments with these transformed strains, including perturbations (oxidative stress via H2O2, diamide and hypoxia) combined with other biosensors for GSH redox state and hydrogen peroxide) mutants and inhibitors, assessed the functionality of NAPstar and revealed new information on NADP(H). The NADPH/NADP ratio in the cytosol is lower than suggested from previous measurements. Importantly, the question of the relative role of glutathione and thioredoxins in hydrogen peroxide removal could be investigated, showing that GSH has the more prominent role across kingdoms. Comparison of the response of a previously proposed NADP(H) sensor NERNST with NAPstar and Grx1-roGFP, suggested that it is too influenced by GSH redox state to be reliable for NADPH/NADP measurement.
Overall, this manuscript reports an important new tool to probe metabolism and redox /antioxidant processes. It is a highly significant advance of wide interest.

OUR RESPONSE: We are very grateful for this appreciative and supportive assessment.

I have two comments which the authors could consider.

Line 482 onwards. I found the discussion/presentation of the NADPH/NADP ratio confusing. It is suggested that NADP is less reduced than suggested by previous measurements, but the authors also show that NAPstar cannot measure ratios above 5. Perhaps this could be clarified by stating the typical ratios found in unperturbed cells more explicitly. The figures for expression in cells show the fluorescence signals and the reader needs to look back at the in vitro data to get an idea of the NADP oxidation state. It would be useful to address this presentational issue in the text or figures.

OUR RESPONSE: We thank the reviewer for pointing out this very important issue and hope that our response to it has now removed any potential for confusion. There are several aspects to this comment, which we address one-by-one below:

i) Values reported for 'typical' NADPH:NADP$^+$ ratios in cell and tissue lysates and extracts vary widely. In plants for example, whole tissue NADPH:NADP$^+$ ratios are typically reported to be in the range of 1:1 to 2:1, which is of a similar magnitude as that reported by NAPstar sensors for the cytosolic

$NADPH:NADP^+$ ratio. Please also see our recent review for a summary of the situation in plants (Smith et al, Trends Plant Sci., 2021). In other organisms, including yeast and mammalian cells, literature values for whole cell or tissue lysate $NADPH:NADP^+$ values vary **widely**. For example, Pollak and colleagues (Pollak et al, J. Biol. Chem., 2007) reported an $NADPH:NADP^+$ ratio of ~1:4 using an HPLC-based approach on HEK293 cell lysate which would be fully consistent with our NAPstar measurements. In contrast, Sallin and co-workers, recently reported a $NADPH:NADP^+$ of ~75:1 in the 'cytosol' of HeLa cells using a TCSPS-FLIM approach, whilst a value of 244:1 was reported in mouse liver cells (Lu et al, Antiox. Redox Signal., 2018). Therefore, reported ratios differ by almost 3 orders of magnitude. The source of this huge variation, and whether it is technical or biological in nature, is currently completely unclear but we believe urgently needs to be addressed. **We have amended the discussion to reference extra studies and expand our explanation of this topic.**

ii) The reviewer is of course completely correct in their statement that, based on all our *in vitro* characterization, NAPstars cannot measure $NADPH:NADP^+$ ratios above ~5:1. If cytosolic $NADPH/NADP^+$ ratios were more reduced than ~5:1, the NAPstar probes would not be able to measure this. However, it is important to point out that in yeast, plant and mammalian cells, we have NAPstar variants that allow fully dynamic measurements, i.e. they respond to both reduction and oxidation of the cytosolic NADP pool. That provides a robust basis for our conclusion that the $NADPH/NADP^+$ status in the cytosol of the cell systems investigated is lower than often anticipated: NAPstar 4.3 for example is able to indicate reductive shifts in NADP redox state even though it is saturated at $NADPH/NADP^+$ ratios of 5:1 or above. At higher $NADPH/NADP^+$ ratios, or with NAPstars such as NAPstar3, the sensors are non-responsive to further reduction – due to saturation with NADPH. These sensors, as we have demonstrated can readily respond to oxidation of the NADP redox couple.

iii) We understand the concern about the presentation of the ratiometric biosensor data. On the one side, the sensors respond to $NADPH/NADP^+$ ratios. On the other hand, the sensor status is displayed as fluorescence ratio (TS/mC). The absolute TS/mC ratio can only be compared directly within a given set of experiments where identical equipment and illumination settings were employed. Direct comparison between different experiments or detection techniques, where excitation light source, optical components, detector gain adjustment settings etc will differ, is not possible. **Hence the TS/mC data from the *in vitro* calibration cannot be straightforwardly used to assume $NADPH/NADP^+$ status from *in vivo* data. We have added a sentence to the discussion to clarify this point.**

This is a general characteristic of ratiometric fluorescent protein sensors, which are exceptional tools to measure relative and dynamic changes with subcellular resolution *in vivo*, but are usually limited with respect to absolute quantification. The latter requires *in vivo* calibration. Although, such regimes can be applied for some sensors (e.g., free calcium sensors or the glutathione redox sensor, Grx1-roGFP2), *in vivo* calibration generally bears a high risk of introducing artefacts. We currently have no reliable *in vivo* titration regime for predictably modulating the NADP redox status (we have attempted different procedures including permeabilization with digitonin, as well as driving the sensor to its extremes, which works for NADP oxidation – using strong oxidant – but not for NADP reduction). This is an issue of intrinsic NADP physiology rather than a specific limitation of NAPstars. Like for most other sensors, we therefore focus on the relative changes in NADP redox status, which are readily and reproducibly measurable and provide robust novel insights.

Arguably, I think it would be desirable to repeat some of the experiments to make actual NADP/NADPH measurements in extracts for comparison with NAPstar. I realise the point of the biosensor is to have specificity for subcellular compartments but, particularly for the GR mutants, it would provide a useful comparison/verification of the trend.

OUR RESPONSE: We have now performed additional assays using a commercially available kit (Amplite® Colorimetric NADP/NADPH Ratio Assay Kit, AAT Bioquest; used before e.g. in Peralta et al, Nat. Chem. Biol., 2015) to determine total NADP and $NADP^+$ (and as a result NADPH and NADPH/$NADP^+$) form cellular extracts. We performed those assays to benchmark the NAPstar measurements in Fig. 2 a-e (yeast wild type, glr, zwf1 treated with external H_2O_2) and Fig. 6 (plant wild type, *gr1* treated with H_2O_2). In short, these measurements, despite repeated attempts and optimization were wildly variable in yeast and unfortunately provided no useful information.

In plants, we encountered similar problems. The assays did not reveal any significant changes at the level of whole cell extracts and showed very high variability. Nonetheless, we were able to obtain some data, that we provide here exclusively for our reviewers, showing a high degree of variability and no significant difference between any plant line irrespective of treatment with H_2O_2 (see below).

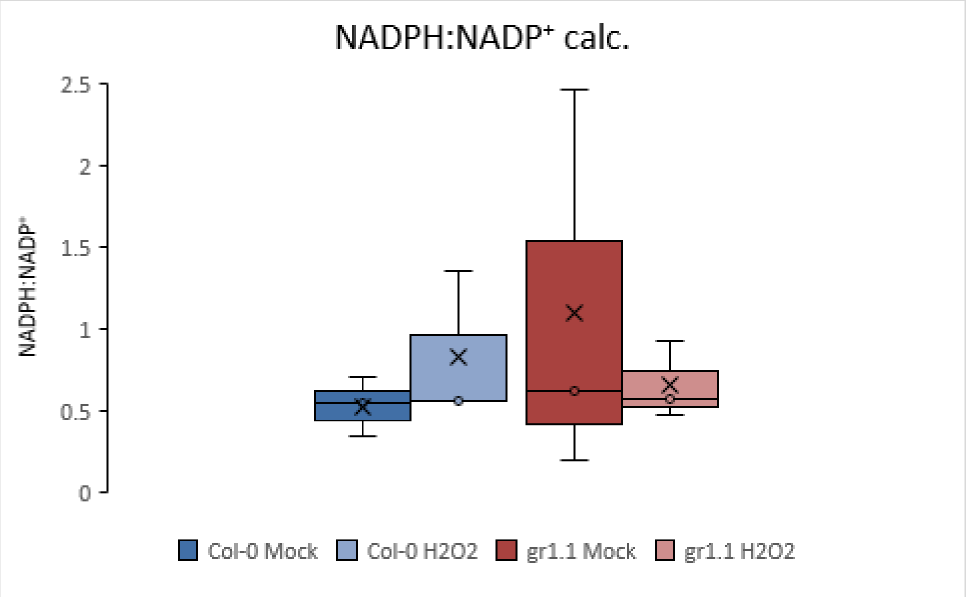

We need to add that we have major reservations about these analytical assays, even beyond the fact that they quantify cellular averages. We have used different NADPH and $NADP^+$ quantification assays relatively broadly and have to conclude that they are remarkably unreliable and provide vastly different numbers depending on the specific assay or kit.

Interestingly, during our further characterisation of the NAPstar and Peredox sensors in yeast, we observed a phenomenon that may help explain some of the issues with measurements in whole cell lysates. Briefly, we took yeast cells expressing cytosolic variants of different NAPstar sensors or Peredox-mCherry for measuring NADH/NAD^+. We then treated these cells with 0.1% digitonin to allow for a mild 'semi-permeabilisation' of the plasma membrane. We then treated the cells with NADH or NADPH at different concentrations and monitored the NAPstar or Peredox-mCherry responses. As expected, all NAPstars variants tested responded to NADPH at concentrations as low

as 10 µM. Response to NADH was only observed at concentration of 1 mM or higher, fully consistent with our previously determined Kd's for NADPH and NADH. **NAPstars thus behave as expected.**

However, intriguingly, **Peredox-mCherry responded well to both NADH and to NADPH**, completely at odds with *in vitro* measurements. This is not a Peredox-mCherry specific effect as we also observed it with SoNar. We interpret these data to mean that there is an efficient conversion of NADPH to NADH in disrupted cells. However, the underlying mechanism is completely unclear at present. This being said, we think it would be crucially important in the future to test whether similar effects can be observed in other cells types. If so, it would force a careful reevaluation of all lysate and tissue extract measurements. However, this is far beyond the scope of this paper (see below).

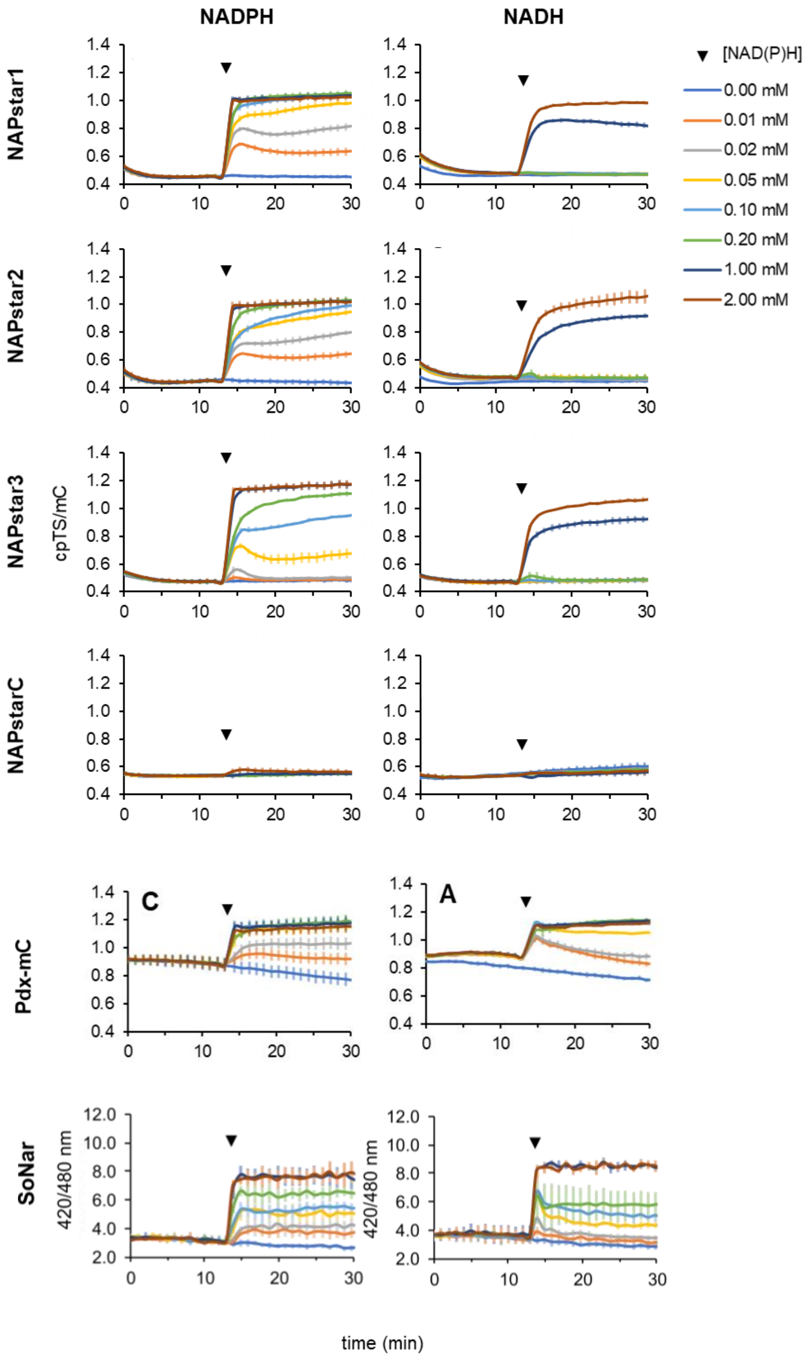

We believe that this data, whilst not yet understood, further demonstrates the huge potential for novel genetically encoded sensors, including NAPstars, to unveil a wide range of novel and previously inaccessible insights into cellular pyridine nucleotide homeostasis.

Reviewer #2 (Remarks to the Author):

This manuscript describes the production and application of an NAD redox state sensor, that is based on Peredox-mCherry, in which fluorescence excitation and emission spectra are resistant to pH changes. The authors clearly describe the advantages offered by NAPstars relative to other NADPH-NADP+ biosensors. The possibility of measuring real-time,
specific, pH-resistant measurements, across a broad-range of NADP redox states, in different subcellular compartments is certainly an attractive proposition. The authors provide several lines of evidence from different systems using appropriate approaches to validate the probes used and test robustness in in vitro as well as in vivo systems.

OUR RESPONSE: We are very grateful for this appreciative and supportive assessment.

However, I cannot see that the authors attempted simultaneous biochemical measurements of NAD(P)Hand NAD(P+). Such measurements would have been insightful not least to determine how the in vivo measurements differ from the classical extraction and assay methods. The proof of the response ranges for both sensor variants for NADPH/NADP+ in in vito systems, does not directly confirm that each sensor accurately measures the physiological NADP redox state.

OUR RESPONSE: A similar point has been raised by reviewer #1. Please see our extensive response above in which we address these concerns.

The reviewer if of course correct in their statement that data from *in vitro* characterization cannot be used to conclude with absolute certainty that the probes behave identically *in vivo*. This is true not only for <u>any</u> genetically encoded sensor but also for <u>any</u> biochemical approach. Nonetheless, such assumptions underlie the success of the reductionist approach in driving much of modern biology. Furthermore, our characterization in living cells, for example observing expected changes in NADP redox state in response to deletion of major sources of cytosolic NADPH, or in response to removal of carbon sources, strongly supports that NAPstars are readily and specifically responding to real in vivo changes in NADP redox state. Whilst it is impossible to completely rule out any other *in vivo* influence on our sensors, we have no experimental evidence to indicate that such an influence may exist.

While the data presented are convincing, only a small amount of data is presented for each system analysed and this tends to open more questions than answers. For example, a microplate-based assay was used to monitor the response of NAPstars and PeredoxDS to the addition of hydrogen peroxide (0–5 mM), when expressed in the cytosol of the budding yeast, Saccharomyces cerevisiae. Only a small response was observed in the probe, the data indicating a limited response with a rapid recovery. Although the observed dynamic changes in pyridine nucleotide redox state that were observed during the yeast metabolic cycle are readily understandable, some of the other responses such as the lack of response to external hydrogen peroxide are less easy to explain. These findings are interesting but not considered in the discussion section.

OUR RESPONSE: It is in the nature of an article that introduced a novel biosensor for broad use across different system that each of those systems can only be highlighted to some degree.

Nonetheless, we have now added extensive extra datasets, particularly in the yeast system, to further illustrate our confidence in the NAPstars and the resulting datasets. Primarily, we have now repeated all yeast experiments presented in Figure 2 with both H_2O_2 (old panels in Figure 2 have been replaced with new datasets) and *tert*-Butyl hydroperoxide (*t*-BuOOH; new Supplementary Figure 7) as oxidants. Furthermore, we now provide extensive comparative analyses of NAPstarC, NAPstar3 and NAPstar6 probes, together with roGFP2, roGFP2-Grx1 and NERNST, in four different genetic backgrounds, with three different oxidants (Supplementary Figure 13,14,15). Our data support the conclusions we drew about the impact on NADPH consumption of deleting glutathione reductase and show that NERNST behaves indistinguishably to an unfused roGFP2 moiety *in vivo*.

We have also performed three extra repeats of the plant data in Figure 6 (new datasets presented in Figure 6 and Supplementary Figure 16). Again, these new datasets support our original findings. In total, this amounts to more than 100 new figure panels.

We agree with the reviewer that the robustness of the cytosolic NADP pool to oxidation by exogenous oxidants is surprising, in the sense that it is indeed surprisingly, and remarkably, robust. However, we do not consider it to be difficult to explain. NAPstar responses in yeast cells deleted for *ZWF1*, which encodes glucose-6-phosphate dehydrogenase, the first enzyme in the pentose phosphate pathway, are strikingly more sensitive as compared to responses in wild-type cells (See datasets in Figure 2 and Supplementary Figures 6,7). This strongly suggests that the robustness against NADP oxidation is real and not a probe artefact. Likewise, in HeLa cells, no NADP oxidation is induced by external H_2O_2 when glucose is available, indeed almost no HyPer7 probe (an ultrasensitive H_2O_2 probe) response is observed in these conditions. However, strong oxidation of the NADP pool is observed in glucose starved cells and strong HyPer7 responses are seen. Again, this supports the conclusion that the robustness of the NADP pool is real, is driving efficient H_2O_2 scavenging, and is not an artefact or limitation of the NAPstar sensors. The source of the robustness in both yeast and HeLa cells seems to be a high flux through the pentose phosphate pathway providing a highly efficient replenishment of NADPH. **Following the advice of the reviewer we have now expanded the discussion to highlight this observation more clearly.**

The studies in illuminated leaf tissue related photosynthesis are particularly interesting, as are the studies on hypoxia, in which there are comparable measurements of the glutathione redox potential using the Grx1-roGFP2 and roGFP2-Orp1 probes. The proposed limitation on glutathione reductase activity under re-oxygenation after hypoxia is insightful, as is the conclusion that the NERNST sensor functions well as a glutathione redox state reporter but does not operate as well for the NADP redox state. However, there is no real discussion of this conclusion or its implications. Although much of the discussion focusses on the relationships between the pyridine nucleotide, glutathione and thioredoxin pools, I struggled to find any real novelty in these findings, which appeared to be largely confirmatory.

OUR RESPONSE: We are very grateful for this appreciative and supportive assessment. We agree with the reviewer that the insights drawn from the dark-light and hypoxia assays in plant tissues deserve more in-depth discussion, which we had omitted for conciseness in the previous version. **We now added more in-depth explanation about the rationale to the Results section and a section dedicated to the interpretation of those data to the Discussion section.**

We do not fully understand the reviewer's comment regarding the lack of novelty in the observation of the relative importance of the glutathione/glutaredoxin system compared to the thioredoxin system. In yeast and mammalian cells, it has been assumed for many years that the thioredoxin system would be the major drain of electrons from the NADP pool for reduction of H_2O_2 and protein disulfides, see for example (Kumar et al, EMBO J., 2011; Mishina et al, Antiox. Redox Signal., 2019; Hoehne et al, EMBO J., 2022). For yeast and mammalians cells were therefore consider our results to be novel and unexpected. In plants there has been evidence of a major contribution of the glutathione pool as a conduit of NADPH-dependent electron flux, with a recent elegant *in vitro* study supporting a major role of glutathione in peroxiredoxin reduction (Vogelsang et al, Redox Biol., 2024). However, conclusive, direct *in vivo* evidence has still been lacking. This is what we provide here. **We have amended the discussion to further clarify these points.**

<u>Reviewer #3 (Remarks to the Author):</u>

Scherschel and coworkers created a family of NADP+/NADPH redox state sensors (termed "NAPstars") based on reverse engineering of the NAD-sensitive Peredox sensor and iNAP sensor. The sensors use two copies of the mutated NADH/NAD+-binding domain of Rex bounded by circularly permuted T-Sapphire and mCherry. After characterizing the sensitivity to redox states and pH, the authors apply the sensors in various organisms including yeast, HeLa cells, Arabidopsis thaliana plants, and HEK293 cells. Unfortunately, (1) the rational sensor design is not clearly articulated, (2) the sensor responses do not appear to solely respond to NADPH/NADP redox state and (3) the sensors appear to be pH-dependent in contrast to their conclusion.

OUR RESPONSE: We thank the reviewer for their analysis, which helped us to clarify several points and to avoid misunderstandings which we believe are part of the reason for the criticism expressed by the reviewer. We have performed additional experiments to clarify the issues where reasonable.

Major issues:
(1) The authors first characterized purified sensor responses to changes in NADPH/NADP and NADH/NAD concentration (Fig. 1). Each sensor shows changes in the TS/mCherry emission ratio in response to NADPH/NADP ratio but they only respond to very high NADH/NAD+ ratio. This evidence is used to conclude that the sensors respond to the NADPH/NADP redox state with different sensitivities for eacg sensor (KD(NADPH) and KR(NADPH/NADP)). However, the sensors show very different shapes depending on holding NADPH or NADP constant (i.e., they do not show the same Kr based on comparing Fig. 1c and 1f). What accounts for this difference? Doesn't this make the sensor response uninterpretable?

OUR RESPONSE: We understand the confusion of the reviewer about an apparent inconsistency between panels c) and f) in Figure 1. However, this is simply a consequence of experimental design and does not reflect a limitation of probe behavior or data interpretability.

Figure 1 panels c) to h) serve to illustrate the responsiveness of NAPstars to both NADPH and $NADP^+$, as well as the preference of NAPstars for NADP over NAD. While the panels illustrate those findings unambiguously, the reviewer correctly points out the different shapes of the resulting titration curves between panels c and f.

However, this difference is entirely expected based on the biochemistry of analyte binding and the specific experimental setups and indeed validates the reliability of the response. In panel c, all NADPH titrations were performed in the presence of a fixed background of 150 µM $NADP^+$. This regime allows the NAPstars to exist in a fully $NADP^+$-bound state at very low NADPH concentrations and a fully NADPH-bound state at high NADPH concentrations. In panel f, $NADP^+$ was titrated against a fixed NADPH concentration that was different for each NAPstar and corresponded approximately to the K_{dNADPH} for each probe. This was necessary to limit the $NADP^+$ concentrations that would be necessary for a complete titration (otherwise for the lower NADPH/$NADP^+$ ratios, $NADP^+$ concentrations beyond the solubility limit would have been required). However, it also generates a situation in which, by definition, each NAPstar would never be expected to be more than 50% NADPH-bound and therefore explains the difference in the shape and range of the titration curves in panels c and f.

To compensate for the inherent limitation of the $NADP^+$ titrations in Fig. 1f, we had (and still have) included Figure S2 where we additionally titrate NADPH/$NADP^+$ ratios at three different total pool sizes. A close to perfect overlay of the three titration curves was observed for NAPstar3, 3b and 4.3.

We have now also repeated the $NADP^+$ titrations for NAPstar 4.3 against different backgrounds of fixed NADPH concentration and compared them to an NADPH titration. We provide this data only for the attention of our reviewers and do not plan to include them in the manuscript. The dataset clearly illustrates the impact of the fixed NADPH background on the shape of the titration curve. Using a higher NADPH background of 126 µM yields a titration curve that is much more similar to that of the NADPH titration and provides an almost identical K_r, i.e., 0.33 compared to 0.35 for the NADPH titration in this experiment. Both these values are slightly different to the K_r = 0.28 that we determined from the dataset presented in Figure 1 and suggest that these values all lie within the range of experimental error.

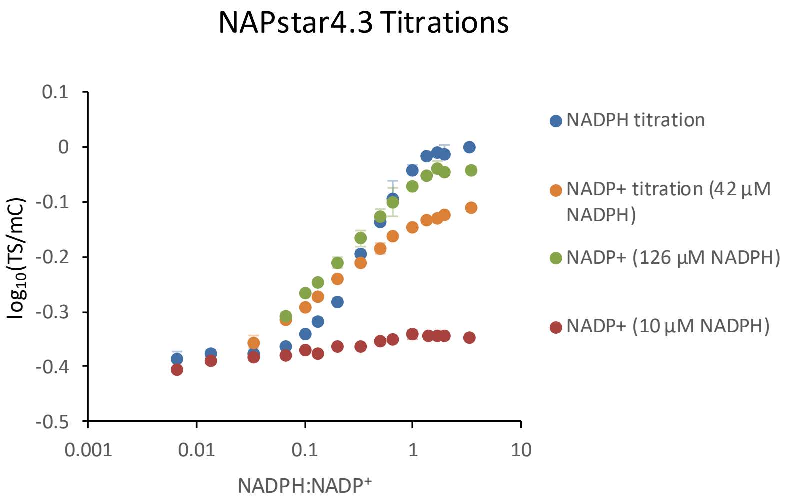

Titration	KR
NADPH (150 µM $NADP^+$ background)	0.35
$NADP^+$ (10 µM NADPH background)	0.16
$NADP^+$ (42 µM NADPH background)	0.25
$NADP^+$ (126 µM NADPH background)	0.33

We continue to transparently present the data in Fig. 1 and S2, including the apparent deviations that the reviewer picked up on, since they jointly make a biochemically conclusive case for the reliability of the response. **We have amended the figure legend to clarify the differences.**

(2) The authors explore the pH sensitivity of the sensor (Supplemental Fig. 3). Based on Fig. 1c and Fig. S2, the maximal response of these sensors is around 0.4-0.6 TS/mC units. When measuring the response to varying pH, the authors state that the NAPstar varied “only ~30%” over 3 pH units concluding that the sensor is insensitive to pH (Fig. S3). However, the change across 3 pH units corresponds to ~0.3 TS/mC units or ~50-75% of the total response. Thus, the impact of pH is rather large. Furthermore, the data appear to show pH impacts NADP binding affinity and thus lowering pH would lower the overall ratio. Doesn’t this make the response uninterpretable?

OUR RESPONSE: We understand the line of argument of the reviewer. Nonetheless, we respectfully but strongly disagree that the interpretability of the NAPstar responses is compromised by pH.

Firstly, we agree that the pH sensitivity of the NAPstars is not zero. This is why we provide the *in vitro* pH titration for full transparency. We further agree that qualitative statements like ‘insensitive to pH’ or ‘resistant to pH’ can hardly ever be quantitatively correct for any protein-based biosensor. Instead, such statements express the observation of a low impact of pH changes on the readout and, in comparison to other biosensors. For NAPstars it is evident that the empirically observed pH-dependent changes are minor as compared to those of other sensors, such as the early HyPer variants 1, 2 and 3 or SoNar, where the pH response within the physiologically meaningful range dwarfs the H_2O_2 or NAD response. Instead, the pH response of NAPstar is very similar to that observed for Peredox-mCherry (Steinbeck et al, Plant Cell, 2020; Fig. 2D), where “*pH sensitivity was eliminated by mutagenesis*” by the Yellen lab (Hung et al, Cell Metabol, 2011).

While the quantitative statements by the reviewer are in agreement with the data we present, the estimation made is hardly physiologically meaningful since the reviewer states the maximal observed pH response that would occur when shifting cytosolic pH across the entire range between ~6.2 and 8.7(!). However, like the case for Peredox-mCherry, the influence of pH is minor between pH 7.0 and 8.5, which is the range typically expected in the cytosol of most cells (up to 20 % of the maximal sensor response). This is especially true for the NADPH-bound NAPstars in which almost no influence of pH is observed between pH 6.3 and 8.5. In addition, we provide NAPstarC as a control which shows an analogous response and is hence the appropriate control in instances where pronounced pH changes are to be expected, as the case for hypoxia as illustrated in Fig. 4g,h. **We have revised the text in the results section to explain this rationale more clearly**.

Even though those two points (minor pH impact in the physiological range and NAPstarC as control in critical measurement applications) place NAPstars amongst the most reliable biosensors we are aware of with respect to realistic pH fluctuations, **we have now re-worded our account of pH-sensitivity of NAPstars throughout the manuscript to be more quantitative, referring to pH-robustness instead of resistance**. Jointly with the data we are convinced that this sets a positive precedence in the literature with respect to clarity.

Finally, we would like to point out that a similarly comprehensive pH analysis as the one provided here is not available for most biosensors. Hence, the argument that the sensor response may not be interpretable based on a transparent approach of comprehensive characterization appears somewhat unfair.

(3) The authors have generated a family of NAPStar sensors but do little to convey how each sensor is designed, unique, and thus useful. They inadequately express/convey the logic behind the various mutations right from the beginning and subsequently do little to convey how each sensor was selected for each experiment. One example (but this occurs throughout) is line 292: “we generated stable transgenic NAPstar3,

NAPstar4.3 and NAPstarC lines with cyto-nuclear sensor expression."
Why were these two sensors used? A one-sentence rationale should be stated to allow the reader to understand.

OUR RESPONSE: We provide full amino acid sequences for all NAPstar variants, which very clearly depict all mutations that were introduced into the 'parental' Peredox probe in order to switch affinity from NADH and NAD^+ to NADPH and $NADP^+$. We also devote an entire paragraph to the description of the Peredox 'parental' probe and the introduced mutations in the beginning of the Results section and provide a structural model of a NAPstar sensor based on an AlphaFold prediction. In our opinion, we have gone far beyond what has been provided for almost any other genetically encoded sensor available in terms of our openness and transparency regarding sensor design. **If the reviewer requests further clarification on specific details of probe design we would of course be happy to provide this.**

We also now point out the rationale for the selection of specific NAPstars for different experiments more clearly, which is based on their characterization as well as the empirically observed responsiveness that indicates a match with physiological NADPH/$NADP^+$ conditions.

(4) This family of sensors appears to respond to NADPH/NADP ratio between 0.0006 and 0.2842 (Fig. 2h), which by their admission is far from the "textbook" accepted ratios of NADPH/NADP. The authors take this to reinterpret the direction of NADPH -> GSH redox biology (lines 494-500). However, an alternative interpretation is that their sensors respond to gross oxidation, but do not respond to physiologically relevant changes. In support of a limited physiological response, Fig. 2c shows the limited response to H2O2 in yeast and Fig. 4b shows the limited response to light of NAPStar4.3 in plants and similar to NAPStarC.

OUR RESPONSE:
We had initially followed the hypothesis drawn up by the reviewer, which indeed proved correct for NAPstars 1 and 2. These sensor variants are indeed only responsive to 'gross oxidation' in the *in vivo* contexts investigated here, in the sense that both sensors are fully saturated with NADPH at 'steady state' and require significant oxidation of the NADP pool in order to respond. However, even for these NAPstar variants, strong increases in response are observed for example in Δ*zwf1* yeast cells and in glucose-starved mammalian cells. Although, it should be While NAPstar1 and 2 provided proof of concept for the design principle, those observations prompted us to generate additional variants for higher NADPH/$NADP^+$ ratios. For example, NAPstar6, 7 and 4.3 all allow to resolve shifts towards BOTH oxidation and reduction of the NADP pool *in vivo* in a number of different experimental settings. They do not only respond to 'gross oxidation' but rather allow fully dynamic monitoring of physiologically relevant changes in NADP redox state, for example during metabolic cycles in yeast and hypoxia-reoxygenation in plants.

The 'limited' response shown in Fig. 2C is towards oxidation, rather than reduction. This 'limitation' is clearly overcome in the delta zwf1 background, which clearly rules out a technical limitation (unresponsive sensor), and rather shows the biological robustness of NADPH/$NADP^+$ through metabolic resupply (please see also our response to reviewer 2 above). This observation is entirely compatible with what has been established for GSH/GSSG considering that it is NADPH that is required for E_{GSH} maintenance. Buffering of E_{GSH} is analogously established in plants and makes robust buffering of the cytosolic NADP pool of plants entirely parsimonious.

Minor issues:

(1) How are the curves normalized? The normalization appears to jump around from experiment to experiment.

OUR RESPONSE: We now provide a clear description about the mode of normalization in each figure legend.

(2) Supplementary Fig 4: There is no consistency in the controls. The control sensor (NAPStarC) is only found in 4a. In referencing, supplemental Fig. 4b the authors state: "Upon the addition of GSSG, we observed a rapid decrease in the TS/mC ratio for each NAPstar construct except NAPstarC." Yet, NAPStarC is not shown.

OUR RESPONSE: We thank the reviewer for picking up this error of ours.

We have now performed the experiment in multiple replicates and ended up including a dataset where NAPstarC was indeed not included. We now replaced the respective datasets. We further used the opportunity to include a full set of controls to illustrate baseline behavior for all assays.

(3) Fig. 4: The NAPStarC changes in Fig. 4 were significant from baseline. The fact that the NAPStarC response was smaller compared to the sensor does not make it "minor".

OUR RESPONSE: We have omitted the term 'minor', which we agree is unnecessarily suggestive.

(4) Line 202: Supplemental figure 5 states Kd(NADPH) = 40.5 uM yet here says NADH. Which is it?

OUR RESPONSE: We thank the reviewer for picking up this mistake. NADPH is correct and we have fixed this.

(5) Fig. 2 and supplemental Fig. 6: Do these cells express catalase which detoxifies H2O2 without using NADPH? Experiments should be redone with tert-Butyl hydroperoxide, which is not detoxified by catalase.

OUR RESPONSE: We are grateful for this comment and have performed a set of experiments using *t*-BuOOH (new Supplementary Figure 7, as well as new Supplementary Figure 14), following the suggestion by the reviewer. We observe a similar impact on NADPH/$NADP^+$ at the qualitative level, validating the H_2O_2 treatments. Yet, we agree that at the quantitative level *t*-BuOOH is more informative since it is expected to draw electrons from the NADP pool stoichiometrically, while the amount of H_2O_2 that is detoxified by catalase is not defined.

(6) Discussion: "NAPstars exhibit several other advantages over currently available NADP sensors including pH-resistance". This is not true based on the discussion above. Also, other sensors have shown pH resistance e.g., mTQ2-based Apollo.

OUR RESPONSE: We have now reworded 'pH resistance' to 'pH robustness' and further amended the discussion to highlight more advantages of NAPstars over other sensors, for example 'accessible detection by fluorescence intensity ratio or fluorescence lifetime'.

Those advantages are described in detail in the Results section and there is no other sensor that unifies those. The comparison with mTQ2-based Apollo is not valid since the Apollo-$NADP^+$ sensor family relies on homo-FRET making its application much less accessible. The required fluorescence anisotropy setup is not standard in life science research, which is probably a reason for the low

numbers of studies that have been making use of Apollo sensors since their introduction over eight years ago. Further, the need for dimerization makes the sensor responses of Apollo dependent on expression levels (similar to SoNar and iNap sensors) which is avoided in NAPstars. Finally, Apollo sensors do not allow analysis of NADPH/NADP$^+$ status since they can only report on NADP$^+$ concentration, which carries orthogonal information.

The reviewer's comment prompted us to highlight a recent study more specifically which reports on the fusion of iNap family biosensors with mCherry, to enable an analysis regime in plants that circumvents the pronounced pH sensitivity that affects the original iNaps (Liu et al, Nat. Comm, 2022). In addition, we added a new reference from the same lab that described the implementation of those iNap-fusions more broadly in plants. The article appeared while this manuscript was under revision: Lim et al, Plant J., 2024. Importantly, iNap and mCherry-iNap sensors report on NADPH concentration and not NADPH/NADP$^+$ as we show for the NAPstars. Hence both sensor families have distinct but complementary applications.

(7) Line 276: Do you mean "previously linked" to cell division as I do not see evidence that you measured cell cycle/division?

OUR RESPONSE: Thank you. Now clarified. The link with cell cycle is made via the well-established dynamics of oxygen uptake as well as the dynamics of H_2O_2 as determined by our previous work that we reference (Amponsah et al, Nat. Chem. Biol., 2021).

(8) Line 408: Revise: "thioredoxin reductases ntr a/b or cytosolic glutathione reductase gr1"

OUR RESPONSE: Thank you. Spelling now corrected to match community standard and 'cytosolic' removed to account for dual targeting to the cytosol and the mitochondrial matrix (NTR a/b) and cytosol and peroxisomes (GR1).

Reviewer #1 (Remarks to the Author):

I am happy with the response of the reviewers to my comments and those of the other reviewers. They have made suitable revisions and provided a thoughtful rebuttal. In particular, it was interesting to learn about the unreliability of NADP/H assays of cell and tissue extracts.

Our Response: We thank this reviewer for their supportive statement and positive evaluation of our work.

Reviewer #2 (Remarks to the Author):

The authors have adequately addressed the points raised.

Our Response: We thank this reviewer for their supportive statement and positive evaluation of our work.

Reviewer #3 (Remarks to the Author):

Most of my concerns have been addressed.

However, one major concern remains:

Genetically encoded sensors commonly suffer from some level of pH dependence. It is appreciated that the authors provided an extensive characterization of the pH sensitivity of their sensors. However, from this evidence the NAPStar sensors cannot be described as “pH robust”.

The control sensor (NAPStarC) shows a significant response to physiological pH (e.g., 7-8) (Fig. S3). Since the control sensor shows a limited subsequent response to NADP+, the pH response is likely due to the fluorophores or to some sort of structural change in the sensors. The “buffer” responses of all the sensors show a similar pH-dependent shape to NAPStarC (Fig. S3 b-h). However, the subsequent responses to NADP+ all varied depending on the pH. For example, NADPStar3 at pH 7.5 and going from 8 to 100 uM went from ~1.2 to ~1.05 TS/mC units. In contrast, there is no change in the ratio in response to NADP at pH 8.5 (i.e., it goes from ~1 to ~1). This change is large when considering the maximal response of these sensors is around 0.4-0.6 TS/mC units. These varied responses suggest pH-dependence in the NADP and/or NADPH binding affinity, which cannot be fully controlled using the NADPStarC alone.

As stated, the authors have done an excellent job of documenting the pH dependence of these sensors and most sensors suffer from some level of this artifact.

Thus, to rectify this:
(1) The authors need to replace the use of “robust” when describing the pH dependence. For example, the sensor could be described as showing a "moderate" pH dependence that can be in part controlled for using NAPStarC.
(2) The authors also need to explicitly state that the NAPStarC is a control for the pH dependence

of the fluorophores and does not fully control for the pH-dependent of the affinity in the binding domains.

Our Response:

We now refer to NAPstars as having a ‘limited pH sensitivity’ throughout the manuscript.

We have also added the following sentence to the results section:

‘*Although, as a note of caution, NAPstarC would not allow for control of potential pH-dependent differences in the binding affinity of NAPstars for NADP+ and NADPH.*
